# Structural Modifications Reveal Dual Functions of the C-4 Carbonyl Group in the Fatty Acid Chain of Ipomoeassin F

**DOI:** 10.3390/molecules30020400

**Published:** 2025-01-18

**Authors:** Arman Khosravi, Precious Nnamdi, Alexa May, Kelsey Slattery, Robert E. Sammelson, Wei Q. Shi

**Affiliations:** 1 Department of Chemistry, Ball State University, Muncie, IN 47306, USA; akhosrav@purdue.edu (A.K.); precious.nnamdi@bsu.edu (P.N.); kelsey.slattery@bsu.edu (K.S.); resammelson@bsu.edu (R.E.S.); 2 Chemistry Department, Michigan State University, East Lansing, MI 48824, USA; mayalex2@msu.edu

**Keywords:** ipomoeassin F, resin glycosides, ring-opened analogues, cytotoxicity, Sec61 translocon

## Abstract

Ipomoeassin F (Ipom-F) is a plant-derived macrocyclic resin glycoside that potently inhibits cancer cell growth through blockage of Sec61-mediated protein translocation at the endoplasmic reticulum. Recently, detailed structural information on how Ipom-F binds to Sec61α was obtained using Cryo-EM, which discovered that polar interactions between asparagine-300 (N300) in Sec61α and four oxygens in Ipom-F are crucial. One of the four oxygens is from the carbonyl group at C-4 of the fatty acid chain. In contrast, our previous structure–activity relationship (SAR) studies suggest that the carbonyl group is not essential. To resolve this discrepancy, we designed and synthesized two new open-chain analogues (**10** and **11**); **10** without the C-4 carbonyl had a dramatic activity loss, whereas **11** with an amide functional group was even more potent than Ipom-F. These new SAR data, in conjunction with some previous SAR information, imply two functional roles of the C-4 carbonyl: (1) to form H-bonds with N300; and (2) to regulate interactions of the fatty acid chain with membrane lipids. Impacts of these dual functions on antiproliferation depend on the overall structure of an Ipom-F derivative. Moreover, **11** can serve as a lead compound for developing future amino acid/peptide-modified analogues of Ipom-F with improved therapeutic properties.

## 1. Introduction

**Figure 1:**
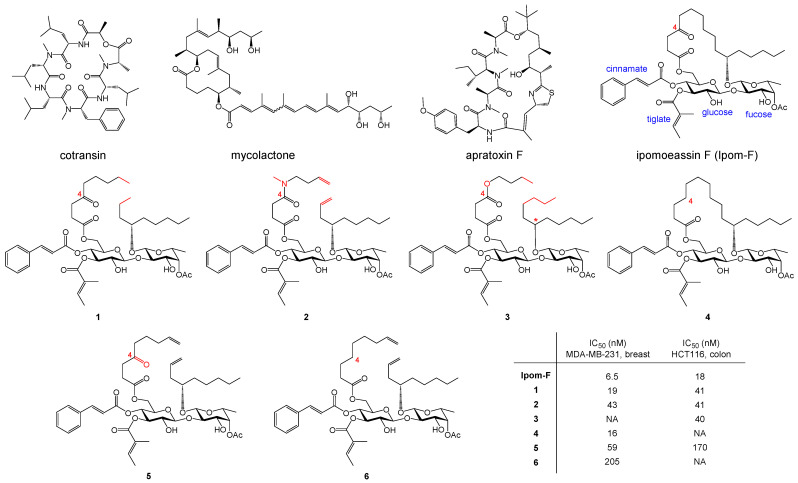
Structures of cotransin, mycolactone A/B, apratoxin F, ipomoeassin F (Ipom-F), and Ipom-F analogues **1**–**6**. Major structural differences among the analogues are highlighted in red. IC_50_s are obtained from refs. [18,19,27]. NA = not available. * indicates the removal of a chirality center.

Natural products provide a rich source of molecular templates for development of novel pharmaceutical agents [1]. To date, six natural products have been discovered to block Sec61-mediated protein translocation [2,3], an essential cellular process for cell survival [4,5,6]. Therefore, they have attracted a great amount of research interest for their promising antibacterial, antiviral, and antitumor activities. Structurally, these natural products can be classified into four categories: depsipeptides (cotransin (Figure 1) [7], coibamide A [8], and decatransin [9]), polyketides (mycolactone A/B (Figure 1) [10,11]), hybrid of depsipeptides and polyketides (apratoxins (Figure 1) [12,13]), and glycolipids (ipomoeassin F (Ipom-F, Figure 1) [14]). Despite distinct differences, all these molecules share a common structural feature, that is, a macrocyclic scaffold with different sizes ranging from 12-membered (mycolactone) to 30-membered ring (decatransin).

Among all the natural Sec61 inhibitors, Ipom-F is unique because it is, to date, the only one isolated from a plant as well as the only one containing carbohydrate fragments (Figure 1). Because of its high potency in inhibiting cancer cell growth in vitro [15], three independent total syntheses have been accomplished [16,17,18] since its discovery. Subsequent medicinal chemistry studies identified the pharmacophore of Ipom-F, that is, two α,β-unsaturated esters (cinnamate and tiglate, Figure 1) [19,20] and the disaccharide core [21]. Thanks to the optimized scalable synthesis [22], further biological and preclinical evaluations confirmed the therapeutic potential of Ipom-F [23,24]. More intriguingly, it was discovered that unlike coibamide A [25], ring-opened analogues of Ipom-F could largely retain biological activities. For example, analogues **1**–**3** (Figure 1) are only 2–4-fold less potent than Ipom-F for inhibiting cancer cell proliferation [19,26].

**Figure 2:**
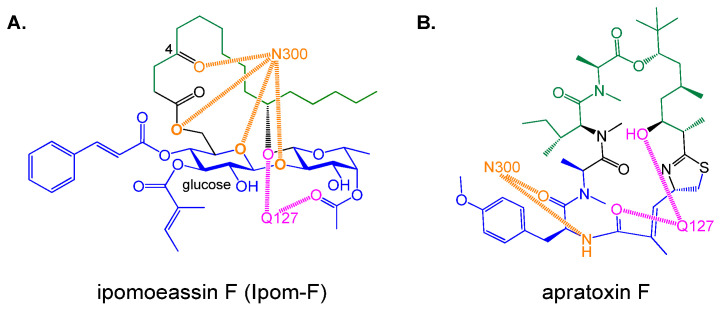
Maps for interactions between Sec61α and Ipom-F (**A**) or apratoxin F (**B**) obtained from cryo-EM. Main lipid-exposed portions are in green whereas channel-facing portions are in blue. Atoms/groups in orange and pink interact with N300 (Asn300) and Q127 (Gln127), respectively.

Very recently, detailed molecular interactions at the atomic level between Sec61α and the aforementioned natural products, except for coibamide A, have been revealed using the Cryo-EM technique [28], which shifts the research paradigm for developing Sec61α inhibitors from phenotype- to structure-based drug design. Among all the interactions, we are particularly attracted by polar interactions between asparagine-300 (N300) in Sec61α and heteroatoms (O/N) in the natural products (Figure 2, in orange). Mutational studies confirmed that substitution of asparagine with alanine (N300A) caused huge antiproliferative activity loss against HEK293 cells (>1000-fold and >100,000-fold for Ipom-F and apratoxin F, respectively) [28], which demonstrates the indispensable role of such polar interactions.

Ipomoeassins belong to a large class of glycolipids called resin glycosides [29]. Compared to other resin glycosides, the ipomoeassins have much higher anticancer activity and comprise two unique structural features: (1) two adjacent α,β-unsaturated esters (cinnamate and tiglate), and (2) the ketone carbonyl group at C-4 of the fatty acid chain. Cryo-EM clearly showed that the carbonyl group at C-4 of the fatty acid chain participated in the critical polar interactions of Ipom-F with N300 in Sec61α (Figure 2A), which suggests that the unique carbonyl group may be a pharmacophore of Ipom-F. However, our structure–activity relationship (SAR) studies delivered contradictory information, that is, removal of the carbonyl group caused only marginal activity losses, no matter in the cyclic (**4** vs. Ipom-F, <3-fold) [19] or acyclic (**5** vs. **6**, <4-fold) [26] scaffold (Figure 1). To rationalize the discrepancy, we propose that the interactions between N300 and three oxygen atoms around the glucose moiety (Figure 2A, in orange and in bold) are sufficiently strong. The interaction between carbonyl and N300 may make only a negligible contribution to the binding of Ipom-F; therefore, we hypothesize that the carbonyl group at C-4 is dispensable. The primary goal of the work reported here is to test this hypothesis. In addition, we hope to further enrich our SAR knowledge on the open-chain system so that we may develop new analogues with better therapeutic properties.

## 2. Results and Discussion

### 2.1. Design of Two New Open-Chain Analogues ***10*** and ***11***

**Figure 3:**
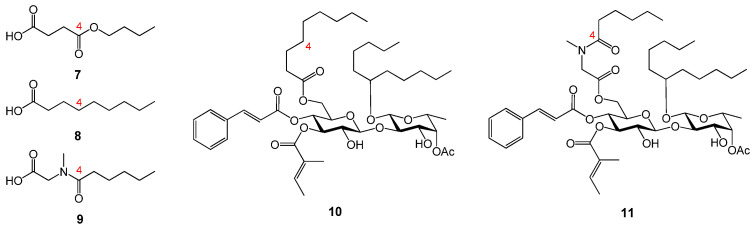
Structures of building blocks **7**–**9** and proposed analogues **10** and **11**.

To date, compounds **1**–**3** (Figure 1) are the most potent open-chain analogues of Ipom-F. Among them, **3** can be more easily adapted to large-scale production [26]. Although mono-butyl succinate **7** (Figure 3), a building block for the synthesis of **3**, could be synthesized efficiently, it has limited stability for long-term storage at room temperature. To further streamline the synthesis of open-chain analogues and simultaneously test whether the carbonyl group at C-4 is essential, we designed analogue **10**, which can be synthesized from commercially available nonanoic acid **8** in replacement of **7**.

Analogue **11** (Figure 3) was also designed for multiple purposes. First, it serves as an extra control compound with a carbonyl group at C-4. Second, the amide analogue **2** (IC_50_: 41 nM) was 4-fold more potent than the ketone analogue **5** (IC_50_: 170 nM) in the viability assay of HCT116 colon cancer cells [26]. More importantly, one of our goals in the future is to incorporate amino acids into the scaffold of Ipom-F because depsipeptides are the largest class of natural Sec61 inhibitors. Additionally, apratoxin F also contains a fragment of tetrapeptide and it is ~100-fold more potent than Ipom-F for inhibiting growth of HEK293 cells [28]. As a starting point, we proposed to replace **7** with *N*-hexanoyl-*N*-methylglycine (**9**) in the synthesis of **11** (Figure 3). Acid **9** could be prepared from commercially available *N*-methylglycine ethyl ester hydrochloride (see Supplementary File S1).

### 2.2. Synthesis of New Open-Chain Analogues ***10*** and ***11***

**Scheme 1:**
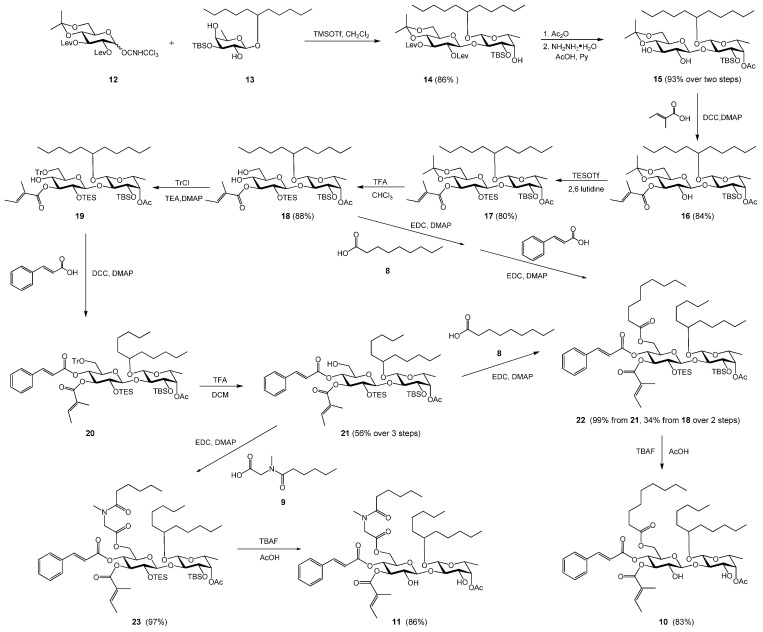
Synthesis of the rationally designed, new open-chain analogues (**10** and **11**) of Ipom-F.

Synthesis of the target molecules started with D-glucose trichloroacetimidate donor **12** [22] and D-fucose acceptor **13** [26] (Scheme 1), which could be prepared by following previously established methods. It was observed that the first glycosylation reaction between **12** and **13** was temperature sensitive. When the temperature was above −30 °C, the acid-labile isopropylidene protecting group tended to break down, leading to a complex mixture of products and poor yield for the desired product [26]. When the temperature was below −65 °C, the reaction became sluggish. Around −50 °C seemed to be optimal for fast coupling of **12** with **13** while minimizing decomposition of isopropylidene. Afterwards, the remaining axial hydroxyl group was reacted with acetic anhydride to give the key disaccharide intermediate **14** in good yield (Scheme 1).

Because the final derivatization would occur at 6″-OH of the glucose moiety, we opted for early removal of the two levulinoyl (Lev) groups by hydrazine under buffered conditions to afford diol **15** in excellent yield (Scheme 1). Next, highly selective esterification of 3″-OH with tiglic acid was achieved to produce **16** efficiently, presumably because 2″-OH was conformationally shielded by the large TBS (*tert*-butyldimethylsilyl) group in the fucose moiety. Based on our previous investigation [22], 2″-OH was then protected by TES using triethylsilyl triflate (TESOTf). At this point, disaccharide **17** was ready for deprotection of isopropylidene by trifluoroacetic acid (TFA) to release 4″-OH and 6″-OH, leading to the formation of diol **18** in very good yield.

Subsequently, we explored selective esterification of 6″-OH in **18** with nonanoic acid **8,** followed by a second esterification with cinnamic acid to deliver the last intermediate **22** (Scheme 1). The advantage of this reaction sequence is that it does not require any protection/deprotection steps. In general, a primary alcohol is expected to be more reactive than a secondary alcohol; therefore, we thought coupling of **18** with **8** would primarily occur at 6″-OH. Unfortunately, we did not observe good selectivity for this particular substrate. In fact, it was not very surprising to us because we observed poor selectivity before on a similar substrate [26]. More importantly, because byproducts generated from the reaction of **18** with **8** had similar polarity to that of the desired product, column purification of the desired product was challenging and the yield over two steps was usually 30–35%.

To improve the overall efficiency, we decided to introduce a bulky protecting group (triphenylmethyl, also known as trityl) to first block 6″-OH in diol **18** (Scheme 1). The yield for the reaction of trityl chloride with **18** was almost quantitative. Next, cinnamic acid reacted with 4″-OH using DCC (*N*,*N′*-dicyclohexylcarbodiimide) as the coupling reagent. Relatively weaker coupling reagents, such as EDC (1-ethyl-3-(3-dimethylaminopropyl)carbodiimide) and CMPI (2-chloro-1-methylpyridinium iodide), could not fully convert **19** to **20**, presumably because of the large steric hindrance caused by the enormous trityl group. After the trityl group was cleaved, the released 6″-OH was then coupled with **8** to give **22**, which could not be purified because it had almost the same R*_f_* as **8**. Extra **8** was removed in the following step. Although this new route added two extra protection and deprotection steps, they were easy to perform and the yield for converting **18** to **22** increased to ~55%. Another advantage to this revised procedure is that different carboxylic acids could be introduced in the penultimate step, which makes future library assembly much more efficient. Finally, both TES and TBS groups were removed by TBAF (tetra-n-butylammonium fluoride) buffered with acetic acid to give the target molecule **10**.

Similarly, target molecule **11** was prepared in two steps from **21** and **9** (see Supplementary File S1 for synthesis and characterization of **9**). Syntheses of the intermediate **23** and the final product **11** were straightforward. However, due to the coexistence of *cis*-*trans* amide bond rotamers, it was difficult to accurately interpret the NMR spectra for both **23** and **11**. Nevertheless, using both ^1^H and ^13^C NMR, we were able to estimate the ratio of the two rotamers in either **23** or **11** was about 3–3.5:1. The identity of the final product **21** was further confirmed by high resolution mass spectrometry (HRMS). The purity of **21** was analyzed by reverse-phase HPLC.

### 2.3. Antiproliferative Activity of New Open-Chain Analogues ***10*** and ***11***

**Figure 4:**
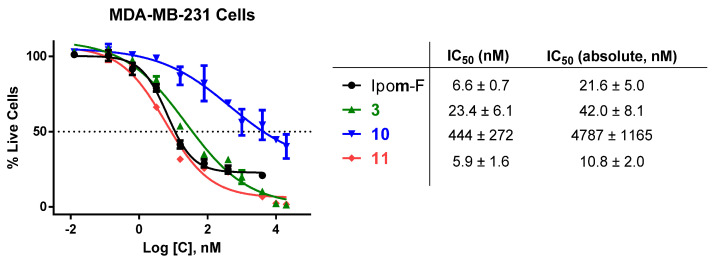
IC_50_s and absolute IC_50_s of Ipom-F and open-chain analogues **3**, **10**, and **11** against MDA-MB-231 breast cancer cells. The data are derived from three independent assay experiments. The cross points of the dashed line with the IC_50_ curves represent absolute IC_50_s.

With the two target molecules in hand, we evaluated their antiproliferative activity quantitatively using alamarBlue assay (ThermoFisher, A50100). MDA-MB-231 cells were used in the assay because this cell line was one of the cell lines that were most sensitive to Ipom-F in the NCI 60-cell line screen [20]. Ipom-F and analogue **3** were selected as positive controls and showed IC_50_s (half-maximal inhibitory concentrations obtained by using Prism 9.5.1, GraphPad) as 6.6 nM and 23.4 nM (Figure 4), respectively, which are consistent with the previously reported values [18,26]. To our surprise, analogue **10** was very inactive. Its IC_50_ was 444 nM, which is more than 65-fold less active than Ipom-F and ~19-fold less potent than 5-oxo analogue **3**. Even at 20 μM, **10** could inhibit only 60–70% cell growth. Therefore, we also used the software to estimate absolute IC_50_s (see Section 3.2.2) as indicated by a dashed line in the dose-response curve (Figure 4), which are 21.6 nM, 42.0 nM, and 4787 nM for Ipom-F, **3**, and **10**, respectively. According to the IC_50_s, **10** is ~230-fold less active than Ipom-F and over 110-fold less potent than analogue **3**. In contrast, the glycine-modified analogue **11** was very active and showed IC_50_ and absolute IC_50_ as 5.9 nM and 10.8 nM, respectively.

**Figure 5:**
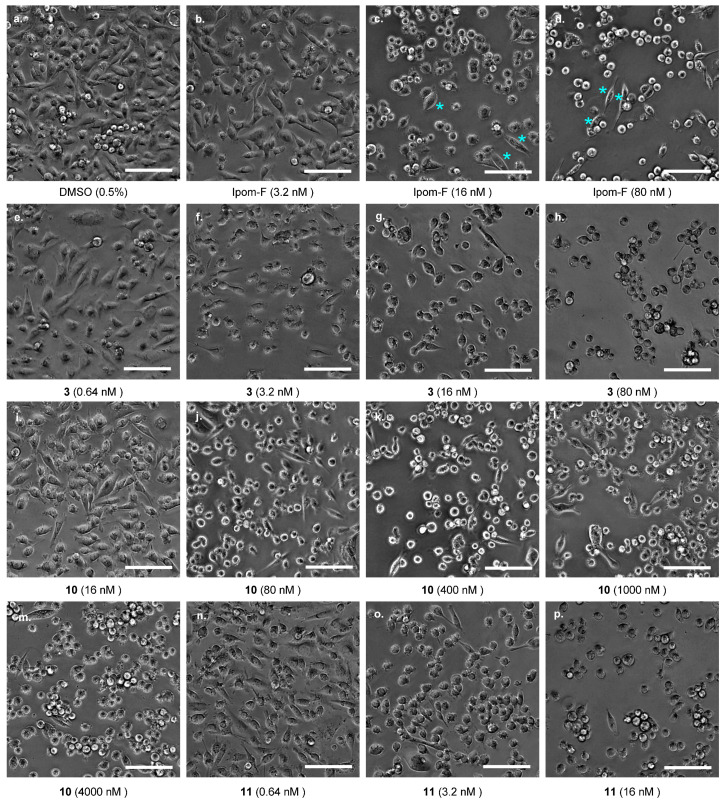
Cell morphology and density changes under treatments of (**a**) DMSO, (**b**–**d**) Ipom-F, or open-chain analogues (**e**–**h**) **3**, (**i**–**m**) **10**, and (**n**–**p**) **11** at different concentrations. Cyan stars in pictures (**c**,**d**) indicate live MDA-MB-231 cells. Scale bar = 100 μm.

To confirm the above results from the quantitative assays, we also monitored changes in cell morphology and density at the end of the drug treatments before adding the alamarBlue dye (Figure 5). Figure 5a shows cell morphology and density for 0.5% DMSO, serving as a negative control. Significant changes in cell morphology and density for Ipom-F occurred at ~16 nM (Figure 5b–d). It was of note that even at higher concentrations (≥80 nM) of Ipom-F, there were always some live cells, as shown by cyan stars in Figure 5c,d, which matches with the IC_50_ curve that flattens out at 75–80% inhibition for Ipom-F (Figure 4). This suggests that the inhibition effect of Ipom-F is primarily cytostatic instead of cytotoxic. For analogue **3**, significant changes in cell morphology and density also occurred at ~16 nM (Figure 5e–h). Unlike Ipom-F, however, very few cells could be seen in the elongated normal morphology at high concentrations (≥80 nM, Figure 5h), which suggests that **3** is likely cytotoxic when concentrations are high.

When the cells were treated with analogue **10**, we appreciated significant cell morphology changes at 80 nM (Figure 5j) when compared to 16 nM (Figure 5i). However, we still saw live cells with the elongated normal morphology even up to 1 μM (Figure 5l). More importantly, compared to Ipom-F and **3**, cell density was significantly higher (Figure 5k–m), even when the concentration of **10** reached its solubility limit, 20 μM. This suggests that cell growth arrest by **10** was temporary. Over time, the cells escaped from the arrest and continued to proliferate. Observations for cell morphology and density changes for analogue **11** largely mirrored what was observed for Ipom-F and **3** but at lower concentrations (0.64–16 nM, Figure 5n–p), which proves that **11** is more potent. To conclude, the results from the qualitative analyses of cell morphology and density support the EC_50_/IC_50_ values from the quantitative assays.

Analogues **1, 3** (Figure 1), and **10** (Figure 3) share great structural similarity. The major difference between them is that **1** and **3** have a carbonyl group at C-4, which is missing in analogue **10**. Given the significant activity loss for analogue **10** when compared to **1** (IC_50_: 18.8 nM against MDA-MB-231 cells [19]) and **3**, it is clear that the carbonyl group at C-4 is essential in this case. Our rationale is that without the carbonyl group, the straight lipid chain in nonanoate can form tight packing with membrane lipids in the lateral gate, which may significantly distort molecular conformation of **10**, therefore weakening its binding to Sec61α. On the other hand, when compared to Ipom-F (Figure 1), analogue **4** (Figure 1) misses the C-4 carbonyl group but is still very active (IC_50_: 16.1 nM against MDA-MB-231 cells) [19]. We think that this can be attributed to the macrocyclic framework of analogue **4** that would restrict any major conformational changes. Moreover, the only structural difference between analogues **5** and **6** (Figure 1) is the presence or absence of the C-4 carbonyl group, but they have rather similar antiproliferative activity (IC_50_: 59.1 nM and 205 nM, respectively, ~3.5-fold) against MDA-MB-231 cells [19,26]. Here, the flat distal alkene may prevent strong interactions between 8-nonenoate and membrane lipids, therefore helping retain the conformation for the most part. Putting all the information together, we believe that the carbonyl group at C-4 has two functional roles. The major role is to achieve balanced hydrophobic interactions with membrane lipids while minimizing conformational distortion. The minor role is to form polar interactions with N300 in Sce61α.

In support of our analyses above, analogue **11** with a carbonyl group at C-4 fully restored the antiproliferative activity. It is even more intriguing that **11** is the first open-chain analogue that showed potency superior to Ipom-F, albeit slightly. Besides the right conformation of **11**, it is likely that through resonance, the electron density on oxygen of the carbonyl group is enhanced, which strengthens its polar interaction (likely hydrogen bonding) with N300. This activity augment was also observed between analogues **2** and **5** (IC_50_: 41 nM and 170 nM, respectively, against HCT116 colon cancer cells, and IC_50_: 43 nM and 59 nM, respectively, against MDA-MB-231 cells) [26], but not in the cyclic system [27], which suggests that the ring-opened system might be better for harvesting polar interactions between N300 and Ipom-F derivatives. More importantly, **11** is the first amino acid-modified analogue of Ipom-F, which warrants further studies of new Ipom-F derivatives containing amino acids/peptides.

## 3. Materials and Methods

### 3.1. Chemical Synthesis General Methods

Unless otherwise stated, all commercially obtained reagents were used without further purification and all reactions were conducted under argon/nitrogen atmosphere. Reaction progress was monitored by TLC using silica gel F254 glass back plates with detection under UV lamp (254 nm) or charring with 5% (*v*/*v*) H_2_SO_4_ (sulfuric acid) in EtOH (ethanol). Column chromatographic purifications were performed using silica gel (70–230 mesh) with a ratio that spanned from 100 to 50: 1 (*w*/*w*) between the silica gel and crude products. All ^1^H NMR spectra were obtained in deuterated chloroform (CDCl_3_), using chloroform (CHCl_3_, *δ* = 7.26) or tetramethylsilane (TMS, *δ* = 0) as an internal reference. All ^13^C NMR spectra were proton decoupled and obtained in CDCl_3_ with CHCl_3_ (*δ* = 77.16) as internal references. NMR data are reported in the form chemical shifts (*δ*) in ppm, multiplicity, coupling constants (*J*) in Hz, and integrations. ^1^H data are reported as though they were first order. Other 1D and 2D NMR spectra like ^135^DEPT, COSY, HMQC, and HMBC were collected in addition to ^1^H and ^13^C for new compounds. High resolution mass spectrometry (HRMS) data were acquired by the Mass Spectrometry Lab at the University of Illinois Urbana-Champaign. Purity was analyzed using a Shimadzu HPLC (Kyoto, Japan) with a dual wavelength UV detector set at 254 nm and 280 nm, a RESTEK Ultra reverse phase column (C18, 5 μm, 4.6 × 200 mm), and an isocratic mobile phase of acetonitrile in water.

#### 3.1.1. Synthesis of Compound **14**

The fucoside diol acceptor **13** (1.10 g, 2.54 mmol), glucoside trichloroacetimidate donor **12** (1.57 g, 2.80 mmol, 1.1 equiv.), and crushed activated 4Å molecular sieves (2.8 g) were suspended in 40 mL anhydrous CH_2_Cl_2_. The mixture was stirred under an argon atmosphere for ~30 min at room temperature and then cooled to −52 °C to −58 °C. To the cold reaction mixture was added TMSOTf (41 μL, 0.229 mmol, 0.09 equiv., Apollo Scientific) via a syringe. The reaction was left to stir for 18 min at this temperature and then quenched by addition of a few drops of TEA. The mixture was filtered through a pad of celite, and the filtrate was then concentrated. The resulting residue was purified by column chromatography (10:1→2:1 hexanes–EtOAc) to afford the product **14** as a yellow oil sticky oil (1.82 g, 86%). Its NMR data match with the literature-reported data [26].

#### 3.1.2. Synthesis of Compound **15**

To an ice-cold solution of **14** (1.82 g, 2.19 mmol), TEA (2.44 mL, 17.52 mmol, 8 equiv.), and DMAP (26.8 mg, 0.219 mmol, 0.1 equiv.) in DCM (14 mL) was added Ac_2_O (1.76 mL, 18.61 mmol, 8.5 equiv.) dropwise and the reaction was warmed to room temperature overnight. At this point, TLC (Hex: EtOAc 3:1) showed the reaction was complete. Methanol was added to quench the reaction. DCM was added and the mixture was washed with 1N HCl (2×) followed by saturated NaHCO_3_ wash. The aqueous layers were extracted with DCM twice and the combined organic layers were dried over Na_2_SO_4_. Rotary evaporation of the solvents yielded the crude product as a pale-yellow syrup (1.82 g 95%), which was moved to the next step without further purification.

To an ice-cold solution of the syrup obtained above (350.0 mg, 0.40 mmol) in DCM (5 mL) was added a buffer solution of hydrazine monohydrate (110 mg, 2.20 mmol, 5.5 equiv.) in acetic acid (1.47 mL, 25.6 mmol, 64 equiv.) and pyridine (2.20 mL, 68 equiv.). The mixture was stirred at 0 °C for 30 min and then at room temperature for 1 h. At this point, TLC showed the reaction was complete. The mixture was washed with 1M HCl (3×), followed by brine wash. All the aqueous layers were extracted twice by DCM and the combined organic layers were dried over Na_2_SO_4_. After solvent evaporation, the residue was purified by column chromatography (2:1, hexanes–EtOAc) to afford **15** as a white foam (265.0 mg, 98%): ^1^H NMR (400 MHz, CDCl_3_, δ_H_) 4.98 (d, *J* = 3.3 Hz, 1H), 4.54 (d, *J* = 7.7 Hz, 1H), 4.29 (d, *J* = 7.4 Hz, 1H), 3.65–3.88 (m, 4H), 3.44–3.62 (m, 5H), 3.31 (t, *J* = 8.4 Hz, 1H), 3.20 (td, *J* = 9.9, 5.2 Hz, 1H), 3.01 (br s, 1H), 2.04 (s, 3H), 1.32–1.56 (m, 10H), 1.12–1.31 (m, 12H),1.05 (d, *J* = 6.4 Hz, 3H), 0.74–0.87 (m, 15H), 0.09 (s, 3H), 0.07 (s, 3H); ^13^C NMR (100 MHz, CDCl_3_, δ_C_) 170.7 (C=O), 104.2 (O_2_C), 101.1 (O_2_CH), 99.7 (O_2_CH), 79.8 (OCH), 79.0 (OCH), 76.5 (OCH), 73.3 (OCH), 72.9 (OCH), 72.6 (OCH), 72.5 (OCH), 68.6 (OCH), 68.2 (OCH), 62.2 (OCH_2_), 34.0 (CH_2_), 33.4 (CH_2_), 32.1 (CH_2_), 31.9 (CH_2_), 29.0 (CH_3_), 25.8 (C(CH_3_)_3_), 24.8 (CH_2_), 24.2 (CH_2_), 22.5(9) (CH_2_), 22.5(7) (CH_2_), 20.8 (CH_3_), 19.0 (CH_3_), 17.8 (SiC(CH_3_)_3_), 16.4 (CH_3_), 14.1 (2×CH_3_), −4.5 (SiCH_3_), −4.7 (SiCH_3_).

#### 3.1.3. Synthesis of Compound **16**

To an ice-cold solution of **15** (230.0 mg, 0.334 mmol), DMAP (8.3 mg, 0.2 equiv.) and tiglic acid (40.8 mg, 1.2 equiv., Aaron Chemicals) in DCM (5 mL) was added DCC (140.0 mg, 2.0 equiv.). The reaction mixture was stirred overnight while warming up to room temperature. TLC showed the reaction was complete. Hexanes (5 mL) and ether (10 mL) were added, and the mixture was stirred for 20 more minutes. The resulting suspension was then filtered through a pad of celite. After solvent evaporation, the residue was purified by column chromatography (30:1→20:1, hexanes–EtOAc) to afford **16** as a white foam (217.0 mg, 84%): ^1^H NMR (400 MHz, CDCl_3_, δ_H_) 6.80 (q, *J* = 7.1 Hz, 1H), 5.09 (t, *J* = 9.5 Hz, 1H), 5.00 (d, *J* = 3.3 Hz, 1H), 4.66 (d, *J* = 7.5 Hz, 1H), 4.30 (d, *J* = 7.5 Hz, 1H), 3.78–3.94 (m, 2H), 3.68–3.77 (m, 2H), 3.50–3.67 (m, 3H), 3.40–3.49 (m, 2H), 3.30 (td, *J* = 9.9, 5.1 Hz, 1H), 2.04 (s, 3H), 1.72–1.80 (m, 6H), 1.12–1.59 (m, 22H), 1.07 (d, *J* = 6.3 Hz, 3H), 0.74–0.91 (d, *J* = 11.2 Hz, 15H), 0.11 (s, 3H), 0.08 (s, 3H); ^13^C NMR (100 MHz, CDCl_3_, δ_C_) 170.8 (C=O), 167.5 (C=O), 137.3 (=CH), 128.4 (=C), 104.4 (O_2_C), 101.3 (O_2_CH), 99.6 (O_2_CH), 79.8 (OCH), 78.8 (OCH), 75.3 (OCH), 72.9 (OCH), 72.8 (OCH), 72.7 (OCH), 71.7 (OCH), 68.6 (OCH), 68.3 (OCH), 62.3 (OCH_2_), 34.0 (CH_2_), 33.4 (CH_2_), 32.2 (CH_2_), 31.9 (CH_2_), 28.9 (CH_3_), 25.8 (C(CH_3_)_3_), 25.0 (CH_2_), 24.8 (CH_2_), 24.2 (CH_2_), 22.6(5) (CH_2_), 22.6(1) (CH_2_), 20.9 (CH_3_), 18.9 (CH_3_), 17.8 (SiC(CH_3_)_3_), 16.4 (CH_3_), 14.3 (CH_3_), 14.1 (CH_3_), 12.1 (CH_3_), −4.5(2) (SiCH_3_), −4.5(8) (SiCH_3_).

#### 3.1.4. Synthesis of Compound **17**

To a solution of **16** (217.0 mg, 0.286 mmol) and 2,6 lutidine (153.0 mg, 5 equiv.) in DCM (3.0 mL) at room temperature was added TESOTf (151.0 mg, 2.0 equiv., Apollo Scientific). The mixture was stirred for one hour. At this point, TLC showed the reaction was complete. The mixture was washed with 1M HCl (2×), followed by brine wash. All the aqueous layers were extracted by DCM twice and the combined organic layers were dried over Na_2_SO_4_. After the solvents were removed by rotary evaporation, the residue was purified by column chromatography (30:1→20:1, hexanes–EtOAc) to afford **17** as a yellow sticky oil (200.1 mg, 80%): H NMR (400 MHz, CDCl_3_, δ_H_) 6.79 (qd, *J* = 7.0, 1.6 Hz, 1H), 5.00 (t, *J* = 9.3 Hz, 1H), 4.88–4.96 (m, 2H), 4.20 (d, *J* = 7.7 Hz, 1H), 3.87–4.01 (m, 2H), 3.81 (dd, *J* = 9.2, 3.6 Hz, 1H), 3.64 (t, *J* = 10.5 Hz, 1H), 3.57 (q, *J* = 6.4 Hz, 1H), 3.41–3.52 (m, 3H), 3.20 (td, *J* = 9.9, 5.2 Hz, 1H), 2.06 (s, 3H), 1.72–1.82 (m, 6H), 1.14–1.66 (m, 22H), 1.07 (d, *J* = 6.4 Hz, 3H), 0.78–0.96 (m, 24H), 0.40–0.63 (m, 6H), 0.11 (s, 3H), 0.05 (s, 3H); ^13^C NMR (100 MHz, CDCl_3_, δ_C_) 170.9 (C=O), 166.8 (C=O), 137.0 (=CH), 128.7 (=C), 101.6 (O_2_CH), 101.1 (O_2_C), 99.3 (O_2_CH), 82.0 (OCH), 74.9 (OCH), 74.5 (OCH), 74.4 (OCH), 74.0 (OCH), 73.4 (OCH), 72.5 (OCH), 68.8 (OCH), 67.2 (OCH), 62.6 (OCH_2_), 34.6 (CH_2_), 34.2 (CH_2_), 32.4 (CH_2_), 32.0 (CH_2_), 29.0 (CH_3_), 25.9 (C(CH_3_)_3_), 25.2 (CH_2_), 24.6 (CH_2_), 23.1 (CH_2_), 22.7 (CH_2_), 21.0 (CH_3_), 18.8 (CH_3_), 17.7 (SiC(CH_3_)_3_), 16.8 (CH_3_), 14.4 (CH_3_), 14.3 (CH_3_), 14.1 (CH_3_), 12.1 (CH_3_), 6.9 (3×SiCH_2_CH_3_), 5.0 (3×SiCH_2_CH_3_), −4.1 (SiCH_3_), −4.3 (SiCH_3_).

#### 3.1.5. Synthesis of Compound **18**

To an ice-cold solution of **17** (168.0 mg, 0.229 mmol) in CHCl_3_ (3.00 mL) was added TFA (130 mg, 5 equiv.). The mixture was stirred at 0 °C for two hours. At this point, TLC showed the reaction was complete. Water was added. The mixture was then extracted with DCM and washed with NaHCO_3._ All the aqueous layers were extracted with DCM twice and the combined organic layers were dried over Na_2_SO_4._ After the solvents were removed by rotary evaporation, the residue was purified by column chromatography (6:1→4:1, hexanes–EtOAc) to afford **18** as a white foam (168.0 mg, 88%): ^1^H NMR (400 MHz, CDCl_3_, δ_H_) 6.88 (q, *J* = 7.0 Hz, 1H), 4.85–4.99 (m, 3H), 4.21 (dd, *J* = 7.8, 1.9 Hz, 1H), 3.94 (td, *J* = 8.3, 1.9 Hz, 1H), 3.78–3.90 (m, 2H), 3.71 (dd, *J* = 12.2, 4.3 Hz, 1H), 3.41–3.63 (m, 4H), 3.25–3.35 (m, 1H), 2.93 (d, *J* = 5.4 Hz, 1H), 2.60 (s, 1H), 2.09 (s, 3H), 1.77–1.85 (m, 6H), 1.38–1.53 (m, 4H), 1.17–1.38 (m, 12H), 1.08 (d, *J* = 6.4 Hz, 3H), 0.76–0.95 (m, 24H), 0.45–0.63 (m, 6H), 0.11 (s, 3H), 0.07 (s, 3H); ^13^C NMR (100 MHz, CDCl_3_, δ_C_) 171.1 (C=O), 168.8 (C=O), 138.4 (=CH), 128.3 (=C), 101.6 (O_2_CH), 100.5 (O_2_CH), 81.9 (OCH), 79.0 (OCH), 75.3 (OCH), 74.5 (OCH), 74.0 (OCH), 73.7 (OCH), 73.4 (OCH), 70.7 (OCH), 68.9 (OCH), 62.3 (OCH_2_), 34.6 (CH_2_), 34.2 (CH_2_), 32.4 (CH_2_), 32.0 (CH_2_), 25.9 (C(CH_3_)_3_), 25.2 (CH_2_), 24.7 (CH_2_), 22.9 (CH_2_), 22.7 (CH_2_), 21.1 (CH_3_), 17.7 (SiC(CH_3_)_3_), 16.8 (CH_3_), 14.5 (CH_3_), 14.3 (CH_3_), 14.1 (CH_3_), 12.1 (CH_3_), 6.9 (3×SiCH_2_CH_3_), 5.1 (3×SiCH_2_CH_3_), −4.1 (SiCH_3_), −4.3 (SiCH_3_).

#### 3.1.6. Synthesis of Compound **19**

To a solution of diol 18 (168.0 mg, 0.201 mmol), TEA (40.8 mg, 2.0 equiv.), and DMAP (2.5 mg, 0.1 equiv.) at room temperature was added trityl chloride (78.7 mg, 1.40 equiv.). After the reaction mixture was stirred at room temperature overnight, TLC showed the reaction was complete. After solvent evaporation, the residue was purified by column chromatography (40:1→20:1, hexanes–EtOAc) to afford 19 as a white foam (214.0 mg, 99%). The successful installation of the trityl group was confirmed by a quick NMR check. No full NMR characterization was conducted.

#### 3.1.7. Synthesis of Compound **20**

To an ice-cold solution of **19** (214.0 mg, 0.199 mmol), DMAP (4.9 mg, 0.2 equiv.), and cinnamic acid (41 mg, 1.40 equiv.) in DCM (4 mL) was added DCC (164.0 mg, 4.0 equiv.). The mixture was stirred while warming up to room temperature overnight. At this point, TLC showed the reaction was complete. Hexanes (5 mL) and ether (10 mL) was added. The reaction mixture was stirred for another 20 min, and then filtered through a pad of celite. After the solvents were removed by rotary evaporation, the residue was purified by column chromatography (50:1→30:1, hexanes–EtOAc) to afford **20** as a white foam (189 mg, 79%). The presence of the cinnamate group was confirmed by a quick NMR check, but full NMR characterization was not conducted.

#### 3.1.8. Synthesis of Compound **21**

To an ice-cold solution of **20** (189 mg, 0.156 mmol) in DCM (2.00 mL) was added TFA (89.0 mg, 5 equiv.). The mixture was stirred at 0 °C for 30 min. At this point, TLC showed the reaction was complete. The mixture was washed with saturated NaHCO_3_, followed by brine wash. All the aqueous layers were extracted twice with DCM and the combined organic layers were dried over Na_2_SO_4._ After solvents were evaporated, the residue was purified by column chromatography (30:1→5:1, hexanes–EtOAc) to afford **21** as a white foam (107 mg, 71%): ^1^H NMR (400 MHz, CDCl_3_, δ_H_) 7.60 (d, *J* = 16.0 Hz, 1H), 7.42–7.52 (m, 2H), 7.29–7.40 (m, 3H), 6.79 (qd, *J* = 7.0, 1.6 Hz, 1H), 6.31 (d, *J* = 16.0Hz, 1H), 5.26 (t, *J* = 9.5 Hz, 1H), 4.96–5.09 (m, 3H), 4.26 (d, *J* = 7.6 Hz, 1H), 3.99 (dd, *J* = 9.1, 7.6 Hz, 1H), 3.87 (dd, *J* = 9.1, 3.7 Hz, 1H), 3.67–3.77 (m, 1H), 3.49–3.66 (m, 5H), 2.12 (s, 3H), 1.68–1.77 (m, 6H), 1.19–1.64 (m, 16H), 1.13 (d, *J* = 6.4 Hz, 3H), 0.80–1.00 (m, 24H), 0.46–0.66 (m, 6H), 0.18 (s, 3H), 0.11 (s, 3H); ^13^C NMR (100 MHz, CDCl_3_, δ_C_) 171.0 (C=O), 167.0 (C=O), 166.0 (C=O), 146.2 (=CH), 138.2 (=CH), 134.2 (=C), 130.6 (=CH), 129.0 (2×=CH), 128.3 (2×=CH), 128.2 (=C), 116.9 (=CH), 101.4 (O_2_CH), 100.8 (O_2_CH), 81.8 (OCH), 75.0 (OCH), 74.5 (OCH), 74.4 (OCH), 74.0(8) (OCH), 74.0(4) (OCH), 73.5 (OCH), 69.8 (OCH), 68.9 (OCH), 61.9 (OCH_2_), 34.6 (CH_2_), 34.1 (CH_2_), 32.4 (CH_2_), 32.1 (CH_2_), 26.0 (C(CH_3_)_3_), 25.2 (CH_2_), 24.8 (CH_2_), 23.0 (CH_2_), 22.7 (CH_2_), 21.1 (CH_3_), 17.8 (SiC(CH_3_)_3_), 16.8 (CH_3_), 14.5 (CH_3_), 14.3 (CH_3_), 14.2 (CH_3_), 12.1 (CH_3_), 6.9 (3×SiCH_2_CH_3_), 5.0 (3×SiCH_2_CH_3_), −4.0 (SiCH_3_), −4.2 (SiCH_3_).

#### 3.1.9. General Procedure for Steglich Esterification with EDC

At 0 °C, EDC (5 equiv.) was added in one portion to a CH_2_Cl_2_ (4 mL) solution of alcohol (1 equiv.), acid (~1.2 eq) and DMAP (4-dimethylaminopyridine, 2 equiv.). The reaction was stirred overnight while warming to ambient temperature. At this point, TLC (silica, EtOAc–hexanes) showed the reaction was complete. The reaction mixture was quenched with a few drops of methanol and washed sequentially with 1M HCl and saturated aqueous NaHCO_3_. The aqueous layers were back extracted with CH_2_Cl_2_. The combined organic layer was dried over Na_2_SO_4_ and concentrated under reduced pressure. The residue was purified by column chromatography (silica, EtOAc–hexanes) to give the desired compounds.

Analogue **22**: Alcohol **21** (39.6 mg, 0.0411 mmol) was reacted with nonanoic acid **8** (8.0 mg, 0.0506 mmol) to make ester **22**. The crude product was purified by column chromatography (100:1→15:1, hexanes–EtOAc) to afford **22** as a colorless syrup (45.4 mg, contaminated by **8** that could not be separated): R*_f_* 0.70 (6:1 hexanes–EtOAc); ^1^H NMR (400 MHz, CDCl_3_, δ_H_) 7.59 (d, *J* = 16.0 Hz, 1H), 7.43–7.52 (m, 2H), 7.32–7.40 (m, 3H), 6.79 (br q, *J* = 6.0 Hz, 1H), 6.31 (d, *J* = 16.0 Hz, 1H), 5.23 (t, *J* = 8.9 Hz, 1H), 5.09 (t, *J* = 9.8 Hz, 1H), 4.96–5.03 (m, 2H), 4.31 (d, *J* = 7.7 Hz, 1H), 4.17 (d, *J* = 4.0 Hz, 2H), 4.04 (d, *J* = 8.4 Hz, 1H), 3.89 (dd, *J* = 9.2, 3.6 Hz, 1H), 3.50–3.70 (m, 4H), 2.33 (t, *J* = 7.4 Hz, 2H), 2.11 (s, 3H), 1.68–1.78 (m, 6H), 1.17–1.66 (m, 28H), 1.13 (d, *J* = 6.4 Hz, 3H), 0.80–1.01 (m, 27H), 0.46–0.66 (m, 6H), 0.18 (s, 3H), 0.12 (s, 3H); ^13^C NMR (100 MHz, CDCl_3_, δ_C_) 173.5 (C=O), 171.0 (C=O), 167.0 (C=O), 165.6 (C=O), 146.0 (=CH), 138.1 (=CH), 134.2 (=C), 130.6 (=CH), 128.9 (2×=CH), 128.3 (2×=CH), 128.2 (=C), 117.0 (=CH), 101.3 (O_2_CH), 100.6 (O_2_CH), 81.7 (OCH), 75.3 (OCH), 74.5 (OCH), 74.3 (OCH), 74.1 (OCH), 73.6 (OCH), 71.8 (OCH), 69.6 (OCH), 68.8 (OCH), 62.9(OCH_2_), 34.7 (CH_2_), 34.2 (CH_2_), 34.1 (CH_2_), 32.5 (CH_2_), 32.1 (CH_2_), 31.9 (CH_2_), 26.0 (C(CH_3_)_3_), 29.3 (CH_2_), 29.2 (CH_2_), 29.1 (CH_2_), 25.3 (CH_2_), 24.8(1) (CH_2_), 24.7(7) (CH_2_), 23.0 (CH_2_), 22.7(5) (CH_2_), 22.7(2) (CH_2_), 21.1 (CH_3_), 17.9 (SiC(CH_3_)_3_), 16.8 (CH_3_), 14.5 (CH_3_), 14.4 (CH_3_), 14.2 (2×CH_3_), 12.1 (CH_3_), 6.9 (3×SiCH_2_CH_3_), 5.0 (3×SiCH_2_CH_3_), −4.1 (SiCH_3_), −4.2 (SiCH_3_).

Analogue **23**: Alcohol **21** (39.1 mg, 0.0406 mmol) was reacted with acid **9** (10.6 mg, 0.0566 mmol) to make ester **23**. The crude product was purified by column chromatography (50:1→10:1, hexanes–EtOAc) to afford **23** as a colorless syrup (46.0 mg, quantitative). The ratio of the two rotamers (~3:1) was estimated from the ^13^C NMR spectrum. ^1^H NMR and ^13^C NMR are reported for the major rotamer: R*_f_* 0.36 (3:1 hexanes–EtOAc); ^1^H NMR (400 MHz, CDCl_3_, δ_H_) 7.57 (d, *J* = 16.0 Hz, 1H), 7.42–7.51 (m, 2H), 7.31–7.39 (m, 3H), 6.77 (q, *J* = 7.0 Hz, 1H), 6.29 (d, *J* = 16.0 Hz, 1H), 4.95–5.30 (m, 4H), 4.22–4.43 (m, 3H), 4.07–4.19 (m, 1H), 3.82–4.06 (m, 3H), 3.45–3.72 (m, 4H), 3.04 (s, 3H), 2.33 (t, *J* = 7.5 Hz, 2H), 2.10 (s, 3H), 1.66–1.76 (m, 6H), 1.17–1.66 (m, 22H), 1.11 (d, *J* = 6.4 Hz, 3H), 0.80–1.01 (m, 27H), 0.45–0.64 (m, 6H), 0.17 (s, 3H), 0.10 (s, 3H); ^13^C NMR (100 MHz, CDCl_3_, δ_C_) 174.0 (C=O), 170.9 (C=O), 169.1 (C=O), 166.9 (C=O), 165.7 (C=O), 146.2 (=CH), 138.2 (=CH), 134.2 (=C), 130.6 (=CH), 129.0 (2×=CH), 128.4 (2x=CH), 128.2 (=C), 116.9 (=CH), 101.4 (O_2_CH), 100.6 (O_2_CH), 81.8 (OCH), 75.1 (OCH), 74.5 (OCH), 74.4 (OCH), 74.0 (OCH), 73.6 (OCH), 71.7 (OCH), 69.6 (OCH), 68.8 (OCH), 61.5(OCH_2_), 48.9 (NCH_2_), 36.5 (CH_3_), 34.7 (CH_2_), 34.2 (CH_2_), 33.2 (CH_2_), 32.5 (CH_2_), 32.1 (CH_2_), 31.6 (CH_2_), 26.0 (C(CH_3_)_3_), 25.2 (CH_2_), 24.8 (CH_2_), 24.6 (CH_2_), 23.0 (CH_2_), 22.8 (CH_2_), 22.6 (CH_2_), 21.1 (CH_3_), 17.9 (SiC(CH_3_)_3_), 16.8 (CH_3_), 14.5 (CH_3_), 14.4 (CH_3_), 14.2 (CH_3_), 14.1 (CH_3_), 12.1 (CH_3_), 6.9 (3×SiCH_2_CH_3_), 6.9 (3×SiCH_2_CH_3_), −4.0 (SiCH_3_), −4.2 (SiCH_3_).

#### 3.1.10. General Procedure for TES and TBS Removal Using TBAF/AcOH

To a solution of TES and TBS protected compounds (1 equiv.) in THF (2 mL) was added AcOH (37 equiv.) and TBAF (1M solution in THF, 22 equiv., Oakwood) at room temperature. The reaction was then stirred at room temperature for 8 h. At this point, TLC (silica, EtOAc–hexanes) showed the reaction was complete. The reaction mixture was diluted with CH_2_Cl_2_ and washed sequentially with 1M HCl, saturated aqueous NaHCO_3_, and brine. The aqueous layers were back extracted with CH_2_Cl_2_. The combined organic layer was dried over Na_2_SO_4_ and concentrated under reduced pressure. The residue was purified by column chromatography (silica, EtOAc–hexanes) to give desired analogues.

Analogue **10**: The TES and TBS groups in **22** (45.4 mg, 0.0411 mmol) were removed by TBAF/AcOH. The crude product was purified by column chromatography (15:1→2:1, hexanes–EtOAc) to afford **11** as a colorless to pale yellow syrup (29.8 mg, 83%): R*_f_* 0.55 (1:1 hexanes–EtOAc); ^1^H NMR (400 MHz, CDCl_3_, δ_H_) 7.57 (d, *J* = 16.0 Hz, 1H), 7.38–7.48 (m, 2H), 7.26–7.38 (m, 3H), 6.75–6.87 (m, 1H), 6.27 (d, *J* = 16.0 Hz, 1H), 5.10–5.26 (m, 3H), 4.65 (d, *J* = 8.2 Hz, 1H), 4.38 (d, *J* = 7.3 Hz, 1H), 4.24 (br s, 1H), 4.13 (d, *J* = 3.8 Hz, 2H), 3.55–3.84 (m, 7H), 2.24 (td, *J* = 7.6, 3.2 Hz, 2H), 2.14 (s, 3H), 1.63–1.76 (m, 6H), 1.36–1.55 (m, 6H), 1.14–1.35 (m, 22H), 1.12 (d, *J* = 6.4 Hz, 3H), 0.73–0.88 (m, 9H); ^13^C NMR (100 MHz, CDCl_3_, δ_C_) 173.6 (C=O), 171.4 (C=O), 168.1 (C=O), 165.6 (C=O), 146.6 (=CH), 139.2 (=CH), 134.1 (=C), 130.8 (=CH), 129.0 (2×=CH), 128.4 (2×=CH), 127.8 (=C), 116.6 (=CH), 102.8 (O_2_CH), 99.6 (O_2_CH), 79.3 (OCH), 77.7 (OCH), 74.4 (OCH), 72.8 (OCH), 72.2 (OCH), 71.8 (OCH), 70.8 (OCH), 69.3 (OCH), 68.3 (OCH), 62.3 (OCH_2_), 34.4 (CH_2_), 34.0 (CH_2_), 33.4 (CH_2_), 32.1 (CH_2_), 31.9 (2×CH_2_), 29.3 (CH_2_), 29.2(4) (CH_2_), 29.2(2) (CH_2_), 24.7(9) (CH_2_), 24.7(7) (2×CH_2_), 24.7(4) (CH_2_), 22.7(1) (CH_2_), 22.6(8) (CH_2_), 21.1 (CH_3_), 16.4 (CH_3_), 14.6 (CH_3_), 14.2(0) (2×CH_3_), 14.1(7) (CH_3_), 12.1 (CH_3_). HRMS *m*/*z* calcd for C_48_H_74_NaO_14_ [M+Na]^+^ 897.4969, found: 897.4976. Purity: 95.4% (MeCN/H_2_O 97:3; 1.5 mL/min, *t*_R_ = 14.336 min).

Analogue **11**: The TES and TBS groups in **23** (46.0 mg, 0.0406 mmol) were removed by TBAF/AcOH. The crude product was purified by column chromatography (10:1→1:1, hexanes–EtOAc) to afford **11** as a colorless to pale yellow syrup (31.6 mg, 86%). The ratio of the two rotamers (~4:1) was estimated from the ^13^C NMR spectrum. ^1^H NMR and ^13^C NMR are reported for the major rotamer: R*_f_* 0.57 (1:2 hexanes–EtOAc); ^1^H NMR (400 MHz, CDCl_3_, δ_H_) 7.61 (d, *J* = 16.0 Hz, 1H), 7.44–7.52 (m, 2H), 7.32–7.41 (m, 3H), 6.78–6.88 (m, 1H), 6.31 (d, *J* = 16.0 Hz, 1H), 5.11–5.31 (m, 3H), 4.70 (d, *J* = 8.2 Hz, 1H), 4.38 (d, *J* = 6.7 Hz, 1H), 4.22–4.31 (m, 3H), 3.98 (d, *J* = 17.4 Hz, 1H), 3.61–3.90 (m, 8H), 3.03 (s, 3H), 2.33 (t, *J* = 7.4 Hz, 2H), 2.18 (s, 3H), 1.66–1.78 (m, 6H), 1.40–1.66 (m, 6H), 1.18–1.36 (m, 16H), 1.16 (d, *J* = 6.4 Hz, 3H), 0.78–0.90 (m, 9H); ^13^C NMR (100 MHz, CDCl_3_, δ_C_) 174.1 (C=O), 171.4 (C=O), 169.3 (C=O), 167.9 (C=O), 165.7 (C=O), 146.7 (=CH), 139.0 (=CH), 134.0 (=C), 130.8 (=CH), 129.1 (2×=CH), 128.4 (2×=CH), 127.9 (=C), 116.5 (=CH), 102.5 (O_2_CH), 99.6 (O_2_CH), 79.3 (OCH), 77.3 (OCH), 74.2 (OCH), 72.7 (OCH), 72.2 (OCH), 71.8 (OCH), 70.7 (OCH), 69.3 (OCH), 68.3 (OCH), 62.8 (OCH_2_), 49.2 (NCH_2_), 36.5 (CH_3_), 34.4 (CH_2_), 33.5 (CH_2_), 33.2 (CH_2_), 32.0 (CH_2_), 31.9 (CH_2_), 31.6 (CH_2_), 24.8 (2×CH_2_), 24.6 (CH_2_), 22.7 (2×CH_2_), 22.6 (CH_2_), 21.1 (CH_3_), 16.4 (CH_3_), 14.6 (CH_3_), 14.1(8) (CH_3_), 14.1(5) (CH_3_), 14.0(6) (CH_3_), 12.1 (CH_3_). HRMS *m*/*z* calcd for C_48_H_73_NNaO_15_ [M+Na]^+^ 926.4871, found: 926.4878. Purity: 95.1% (MeCN/H_2_O 87:13; 1.0 mL/min, *t*_R_ = 17.144 min).

### 3.2. Biological Analysis

#### 3.2.1. Cell Culture

The MDA-MB-231 breast cancer cell line purchased from ATCC was maintained in a DMEM/HIGH culture medium (Cytiva, SH3028502) supplemented with 10% fetal bovine serum (FBS, Cytiva, SH3039603) and 2 mM L-glutamine (Cytiva, SH3003402), so-called complete medium. Cell cultures were grown in monolayers in a humidified atmosphere of 5% CO_2_ and 95% air at 37 °C (BINDER CB 56 CO2 incubator, Tuttlingen, Germany). The culture medium was changed every 2–4 days based on confluency. Once confluent, cell cultures were passaged once or twice a week using trypsin-EDTA (0.05–0.1%, Cytiva, SH3004202) to detach the cells from their culture dishes. Cell images were taken using an Invitrogen EVOS XL Core Imaging System (AMEX 1000, ThermoFisher, Bothell, WA, USA).

#### 3.2.2. AlamarBlue Viability Assays

Counting of viable cells was performed before each experiment. Experiments were conducted in duplicate or triplicate. First, 75 μL of cell suspension at the density of 40,000 cells/mL was seeded in a 96-well plate (3000 cells/well), which was incubated at 37 °C in 5% CO_2_ for 24 h. The compounds were dissolved in DMSO (dimethyl sulfoxide) to make drug stocks (10 mM). The stock solutions were diluted with the complete DMEM/HIGH medium supplemented with penicillin and streptomycin (Cytiva, SV30010RR2) to make a series of gradient fresh working solutions right before each test. The highest amount of DMSO was controlled to be lower than 0.5%. Subsequently, the cells were treated with 75 μL of the freshly made gradient working solution in the total volume of 150 μL/well for 72 h. After that, 15 μL of alamarBlue stock solution (ThermoFisher, A50100) was added to each well. The plate was then incubated at 37 °C in 5% CO_2_ atmosphere for another 1–3 h and the emission of each well at 590 nm was detected using a Synergy H1 Hybrid multi-mode plate reader (BioTek, Agilent, Santa Clara, CA, USA) at excitation 560 nm.

The percentage viability compared to the negative control (DMSO-treated cells as the 100% scale) and the blank control (medium only without cells as the 0% scale) was determined and Prism 9.5.1 (GraphPad) used to make a plot of viability (%) versus sample concentration and to calculate the concentration at which each compound exhibited 50% inhibition. IC_50_ value estimates were determined using nonlinear regression to fit data between the top and bottom plateaus to a curve of variable slope (four parameters) using the least-squares fitting method. Absolute IC_50_ value estimates were determined using nonlinear regression to fit normalized data between 100% and 0% to a curve of variable slope using the least-squares fitting method.

## 4. Conclusions

Inspired by the lately discovered structural information on small molecule-bound Sec61α [28], we designed two new open-chain analogues (**10** and **11**) of Ipom-F to explore the role of the carbonyl group at C-4 of the fatty acid chain. Both molecules were synthesized successfully and assayed for their ability to inhibit growth of MDA-MB-231 breast cancer cells. After scrutinizing the SAR information derived from the new as well as some related previous analogues (**1** and **3**–**6**), we conclude that the carbonyl group possesses two functional roles. As revealed by the Cryo-EM structure, the carbonyl oxygen forms polar interactions (most likely hydrogen bonding) with N300 in Sec61α. More importantly, we believe that this polar functional group with a *sp*^2^ hybridized carbon would disfavor unnecessarily strong interactions with membrane lipids in the lateral gate that may alter overall molecular conformation, therefore reducing binding affinity to the protein. Contributions of these two functional roles to the overall binding of an Ipom-F derivative to Sec61α vary depending on other parts of the molecule, e.g., cyclic vs. acyclic. Moreover, the work presented here opens a new avenue for future exploration of novel ring-opened Ipom-F analogues containing amino acids/peptides.

## Data Availability

The original contributions presented in this study are included in the article/Supplementary Materials. Further inquiries can be directed to the corresponding author.

## Supplementary Materials

The following supporting information files can be downloaded at: https://www.mdpi.com/article/10.3390/molecules30020400/s1, Supplementary File S1: Chemical syntheses and NMR characterization of carboxylic acid building block **9**; Supplementary File S2: 1D (^1^H and ^13^C) and/or 2D (COSY, HMQC, and/or HMBC) NMR spectra for compounds **10**, **11**, **15**, **16**, **17**, **18**, **21**, **22**, and **23** [30].

## Abbreviations

AcOH: acetic acid; CMPI, 2-chloro-1-methylpyridinium iodide; cryo-EM, cryogenic electron microscopy; DCC, *N*,*N′*-dicyclohexylcarbodiimide; DCM, dichloromethane; DMAP, 4-dimethylaminopyridine; DMSO, dimethyl sulfoxide; EC_50_, half-maximal effective concentration; EDC, 1-ethyl-3-(3-dimethylaminopropyl)carbodiimide); equiv., equiv.alent; ER, endoplasmic reticulum; EtOAc, ethyl acetate; HCT-116, human colorectal cancer cells; HRMS, High resolution mass spectrometry; IC_50_, half-maximal inhibitory concentration; Ipom-F, ipomoeassin F; MDA-MB-231, human epithelial breast cancer cells; Lev, levulinoyl; N300, asparagine-300; NCI, National Cancer Institute; SAR, structure–activity relationship; Sec61α, alpha subunit of the Sec61 complex; TBAF, tetra-n-butylammonium fluoride; TBS, *tert*-butyldimethylsilyl; TEA, triethylamine; TES, triethylsilyl; TESOTf, triethylsilyl triflate; TFA, trifluoroacetic acid; TLC, thin-layer chromatography; TMSOTf, trimethylsilyl triflate.
