# Supplementary material for: Structural Modifications Reveal Dual Functions of the C-4 Carbonyl Group in the Fatty Acid Chain of Ipomoeassin F
Source: Molecules. 2025 Jan 18;30(2):400. doi: 10.3390/molecules30020400 (PMC11767275; doi:10.3390/molecules30020400)
Supplement: Supplementary file 1 [file molecules-30-00400-s001.zip › Supplementary File S1.pdf]

Supplementary Information 1:

Structural Modifications Reveal Dual Functions of the C-4 Carbonyl Group in the Fatty Acid Chain of Ipomoeassin F

Arman Khosravi, Precious Nnamdi, Alexa May, Kelsey Slattery, Robert E. Sammelson and Wei Q. Shi

Table of Contents

Chemistry Part I:<br>Synthesis of carboxylic acid intermediate **9**	S2-S5
References	S6
NMR spectra of **S3** and **9**	S7-S18

Part I: Synthesis of carboxylic acid 9

General methods: Reactions were carried out in oven-dried glassware. Unless otherwise stated, all commercially obtained reagents were used without further purification and all reactions were conducted under argon/nitrogen atmosphere. Reaction progress was monitored by TLC using silica gel F254 glass back plates with detection under UV lamp (254 nm) or charring with 5 % (v/v) H_2SO_4 (sulfuric acid) in EtOH (ethanol). Column chromatographic purifications were performed using silica gel (70 – 230 mesh) with a ratio that spanned from 100 to 50: 1 (w/w) between the silica gel and crude products. All ^{1}H NMR spectra were obtained in deuterated chloroform ($CDCl_3$), using chloroform ($CHCl_3$, δ = 7.26) or tetramethylsilane (TMS, δ = 0) as an internal reference. All ^{13}C NMR spectra were proton decoupled and obtained in $CDCl_3$ with $CHCl_3$ (δ = 77.16) as internal references. NMR data are reported in the form: chemical shifts (δ) in ppm, multiplicity, coupling constants (J) in Hz, and integrations. ^{1}H data are reported as though they were first order. Other 1D and 2D NMR spectra like ^{135}DEPT, COSY, HMQC, and HMBC were collected in addition to ^{1}H and ^{13}C for new compounds. High resolution mass spectrometry (HRMS) data were acquired by the Mass Spectrometry Lab at the University of Illinois Urbana-Champaign. Purity was analyzed using a Shimadzu HPLC with a dual wavelength UV detector set at 254 nm and 280 nm, a RESTEK Ultra reverse phase column (C18, 5 μm, 4.6x200 mm) and an isocratic mobile phase of acetonitrile in water.

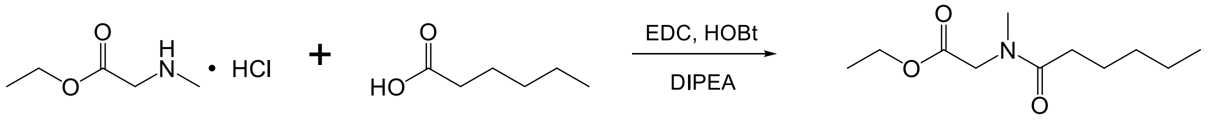

S1 **S2** **S3** (92%)

Synthesis of ethyl *N*-hexanoyl-*N*-methylglycinate S3 [1]: To an ice-cold solution of hexanoic acid **S2** (272.0 mg, 2.34 mmol, 1.2 equiv) in DCM (6 ml) was added EDC (561.0 mg, 2.93 mmol, 1.5 equiv) and HOBt (449.0 mg, 2.93 mmol, 1.5 equiv). After 10 minutes, sarcosine ethyl ester hydrochloride **S1** (300 mg, 1.95 mmol) was added, followed by addition of DIPEA (757 mg, 5.86 mmol, 3 equiv) dropwise. The mixture was stirred overnight while warming up to room temperature. At this point, TLC (Hex: EtOAc 3:1) showed the reaction was complete. The reaction was then quenched by $NaHCO_3$ and extracted by DCM three times. The combined organic layers were dried over Na_2SO_4. After the solvents were removed under reduced pressure, the resulting

residue was purified by column chromatography (15:1→3:1, hexanes–EtOAc) to afford the product **S3** as a colorless oil (387.0 mg, 92%). The ratio of the two rotamers (~3.75:1) was estimated from the ^{1}H NMR spectrum.

Major rotamer: ^{1}H NMR (400 MHz, $CDCl_3$) δ 4.19 (q, *J* = 7.0 Hz, 2H), 4.12 (s, 2H), 3.08 (s, 3H), 2.38 (t, *J* = 7.6 Hz, 2H), 1.57–1.74 (m, 2H), 1.22–1.40 (m, 7H), 0.83–0.97 (m, 3H); ^{13}C NMR (100 MHz, $CDCl_3$) δ 174.0 (C=O), 169.6 (C=O), 61.2 (OCH_2), 49.5 (CH_2), 36.6 (CH_3), 33.2 (CH_2), 31.6 (CH_2), 24.7 (CH_2), 22.6 (CH_2), 14.2 (CH_3), 14.0 (CH_3).

Minor rotamer: ^{1}H NMR (400 MHz, $CDCl_3$) δ 4.23 (q, *J* = 7.0 Hz, 2H), 4.04 (s, 2H), 2.98 (s, 3H), 2.23 (t, *J* = 7.7 Hz, 2H), 1.57–1.74 (m, 2H), 1.22–1.40 (m, 7H), 0.83–0.97 (m, 3H); ^{13}C NMR (100 MHz, $CDCl_3$) δ 173.7 (C=O), 169.2 (C=O), 61.7 (OCH_2), 51.8 (CH_2), 34.9 (CH_3), 33.0 (CH_2), 31.6 (CH_2), 24.8 (CH_2), 22.5 (CH_2), 14.2 (CH_3), 14.0 (CH_3).

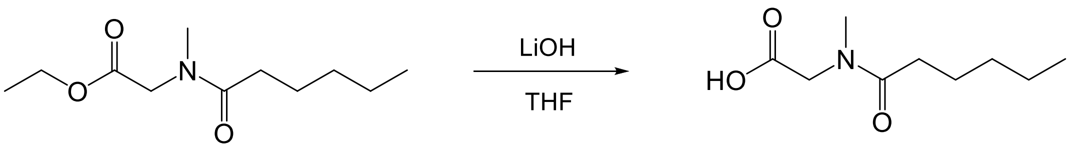

S3 **9** (99%)

Synthesis of *N*-hexanoyl-*N*-methylglycine 9 [1]: To a solution of ethyl *N*-hexanoyl-*N*-methylglycinate **S3** (387.0 mg, 1.8 mmol) in THF (12 ml) was added an aqueous solution of LiOH (0.2 M, 12 ml, 2.4 mmol, 1.33 equiv). The reaction mixture was stirred at room temperature for 1 hour. TLC (Hex: EtOAc 3:1) showed the reaction was complete. The mixture was acidified by 1N HCl at 0 °C and extracted with DCM. The aqueous layer was extracted with DCM twice and the combined organic layers were dried over Na_2SO_4. After filtration, the solvents were evaporated under reduced pressure to afford the product as a colorless oil (333.0 mg, 99%). The ratio of the two rotamers (~3.5:1) was estimated from the ^{1}H NMR spectrum.

Major rotamer: ^{1}H NMR (400 MHz, $CDCl_3$) δ 9.57 (br s, 1H), 4.15 (s, 2H), 3.10 (s, 3H), 2.40 (t, *J* = 7.7 Hz, 2H), 1.53–1.66 (m, 2H), 1.22–1.41 (m, 4H), 0.82–0.98 (m, 3H); ^{13}C NMR (100 MHz, $CDCl_3$) δ 175.2 (C=O), 172.8 (C=O), 49.6 (CH_2), 36.9 (CH_3), 33.2 (CH_2), 31.5 (CH_2), 24.7 (CH_2), 22.5 (CH_2), 14.0 (CH_3).

Minor rotamer: ^{1}H NMR (400 MHz, $CDCl_3$) δ 9.57 (br s, 1H), 4.02 (s, 2H), 2.99 (s, 3H), 2.27 (t, J = 7.7 Hz, 2H), 1.53–1.66 (m, 2H), 1.22–1.41 (m, 4H), 0.82–0.98 (m, 3H); ^{13}C NMR (100 MHz, $CDCl_3$) δ 174.8 (C=O), 171.9 (C=O), 51.7 (CH_2), 35.2 (CH_3), 33.0 (CH_2), 31.5 (CH_2), 24.9 (CH_2), 22.5 (CH_2), 14.0 (CH_3).

References

1. Steinmetz, H.; Li, J.; Fu, C.; Zaburannyi, N.; Kunze, B.; Harmrolfs, K.; Schmitt, V.; Herrmann, J.; Reichenbach, H.; Höfle, G.; et al. Isolation, Structure Elucidation, and (Bio)Synthesis of Haprolid, a Cell-Type-Specific Myxobacterial Cytotoxin. *Angew. Chem. Int. Ed.* **2016**, *55*, 10113-10117, doi:10.1002/anie.201603288.

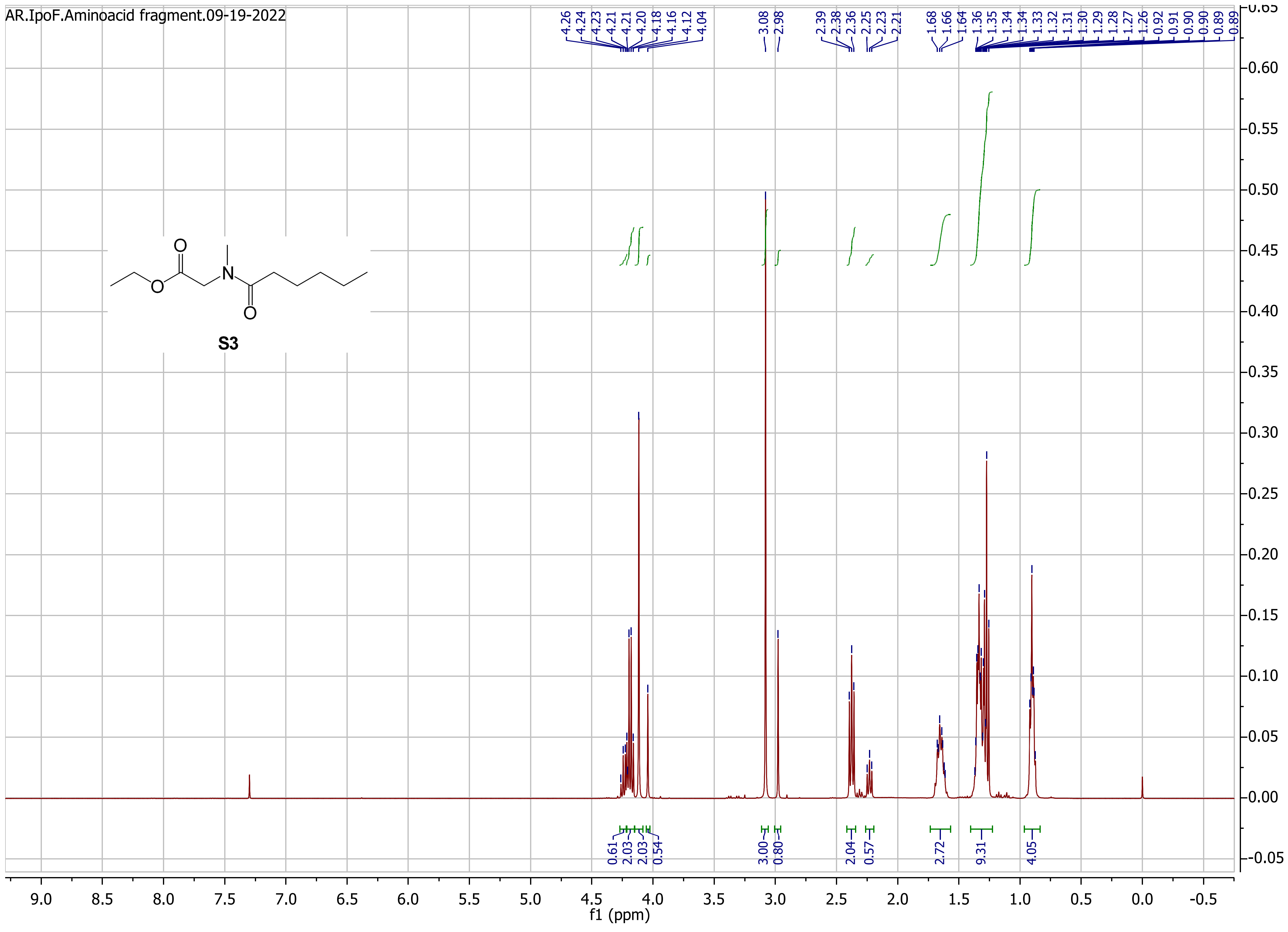

AR.IpoF.Aminoacid fragment.09-19-2022
4.26 4.24 4.23 4.21 4.21 4.20 4.18 4.16 4.12 4.04
3.08 2.98
2.39 2.38 2.36 2.25 2.23 2.21
1.68 1.66 1.64 1.36 1.35 1.34 1.34 1.33 1.32 1.31 1.30 1.29 1.28 1.27 1.26 0.92 0.91 0.90 0.90 0.89 0.89
S3
0.61 2.03 2.03 0.54 3.00 0.80 2.04 0.57 2.72 9.31 4.05
f1 (ppm)

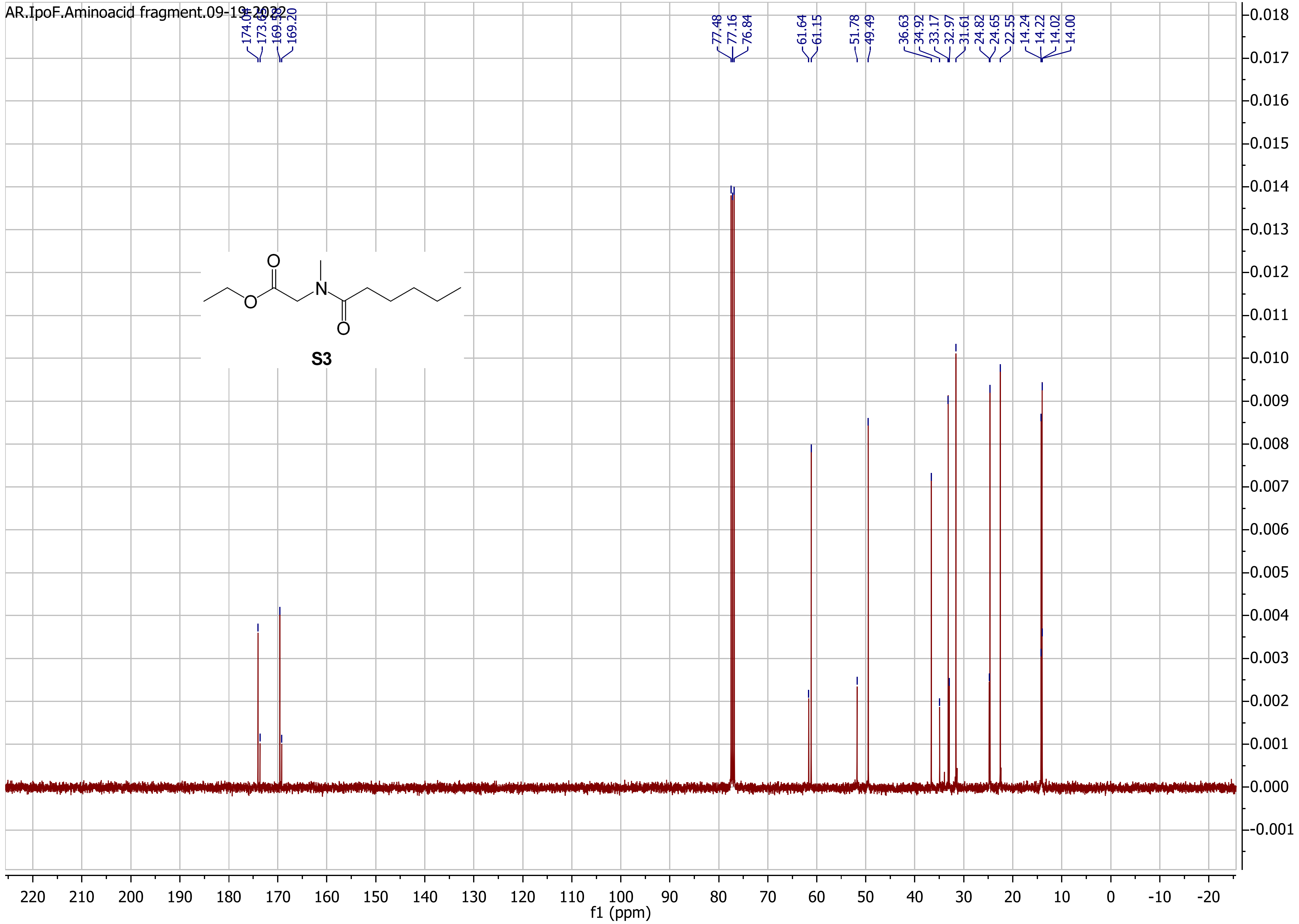

AR.IpoF.Aminoacid fragment.09-19-2022
169.20
77.48
77.16
76.84
61.64
61.15
51.78
49.49
36.63
34.92
33.17
32.97
31.61
24.82
24.65
22.55
14.24
14.22
14.02
14.00
O
N
O
O
S3
220 210 200 190 180 170 160 150 140 130 120 110 100 90 80 70 60 50 40 30 20 10 0 -10 -20
f1 (ppm)
0.018 0.017 0.016 0.015 0.014 0.013 0.012 0.011 0.010 0.009 0.008 0.007 0.006 0.005 0.004 0.003 0.002 0.001 0.000 -0.001

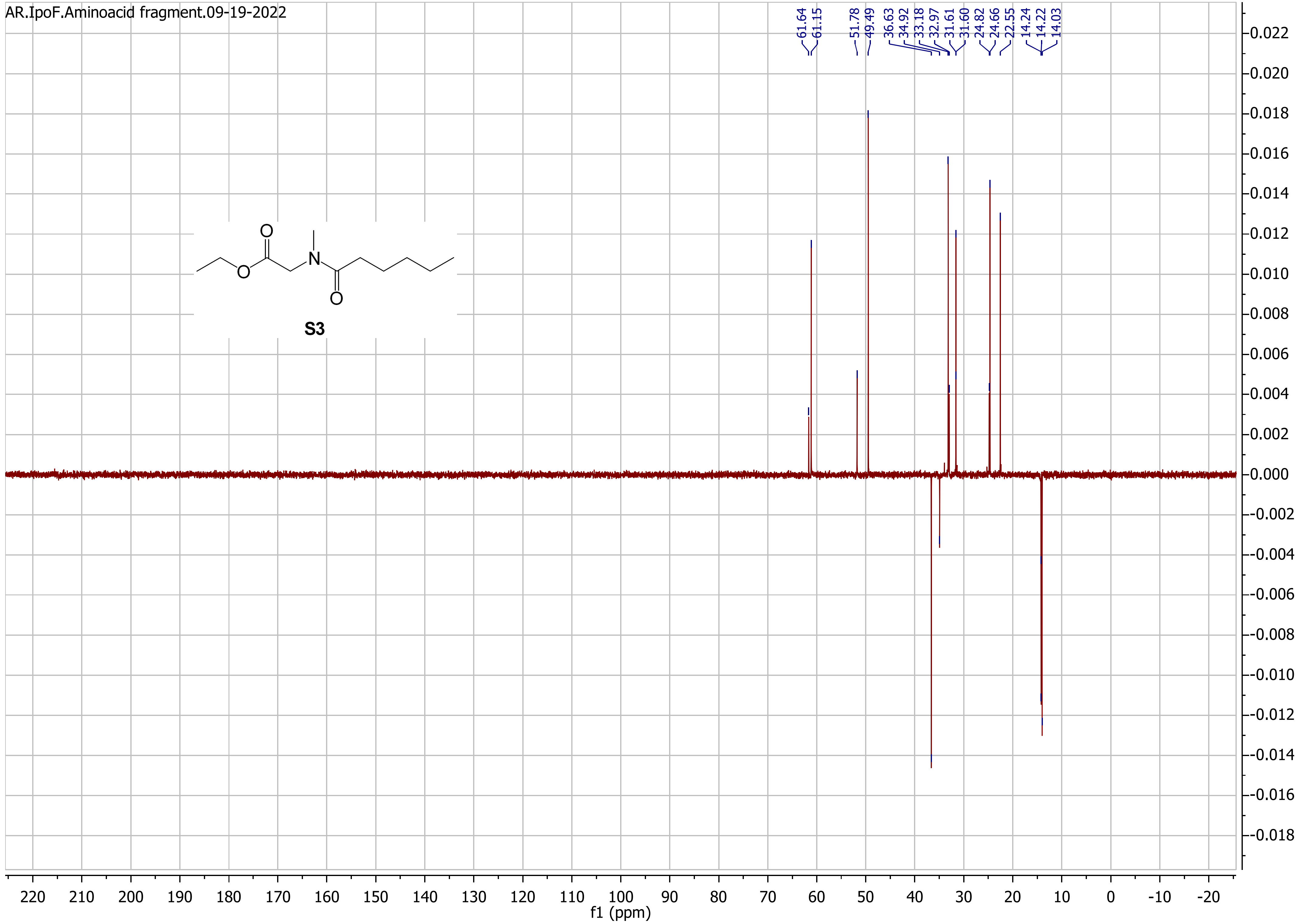
AR.IpoF.Aminoacid fragment.09-19-2022
61.64
61.15
51.78
49.49
36.63
34.92
33.18
32.97
31.61
31.60
24.82
24.66
22.55
14.24
14.22
14.03
S3
f1 (ppm)

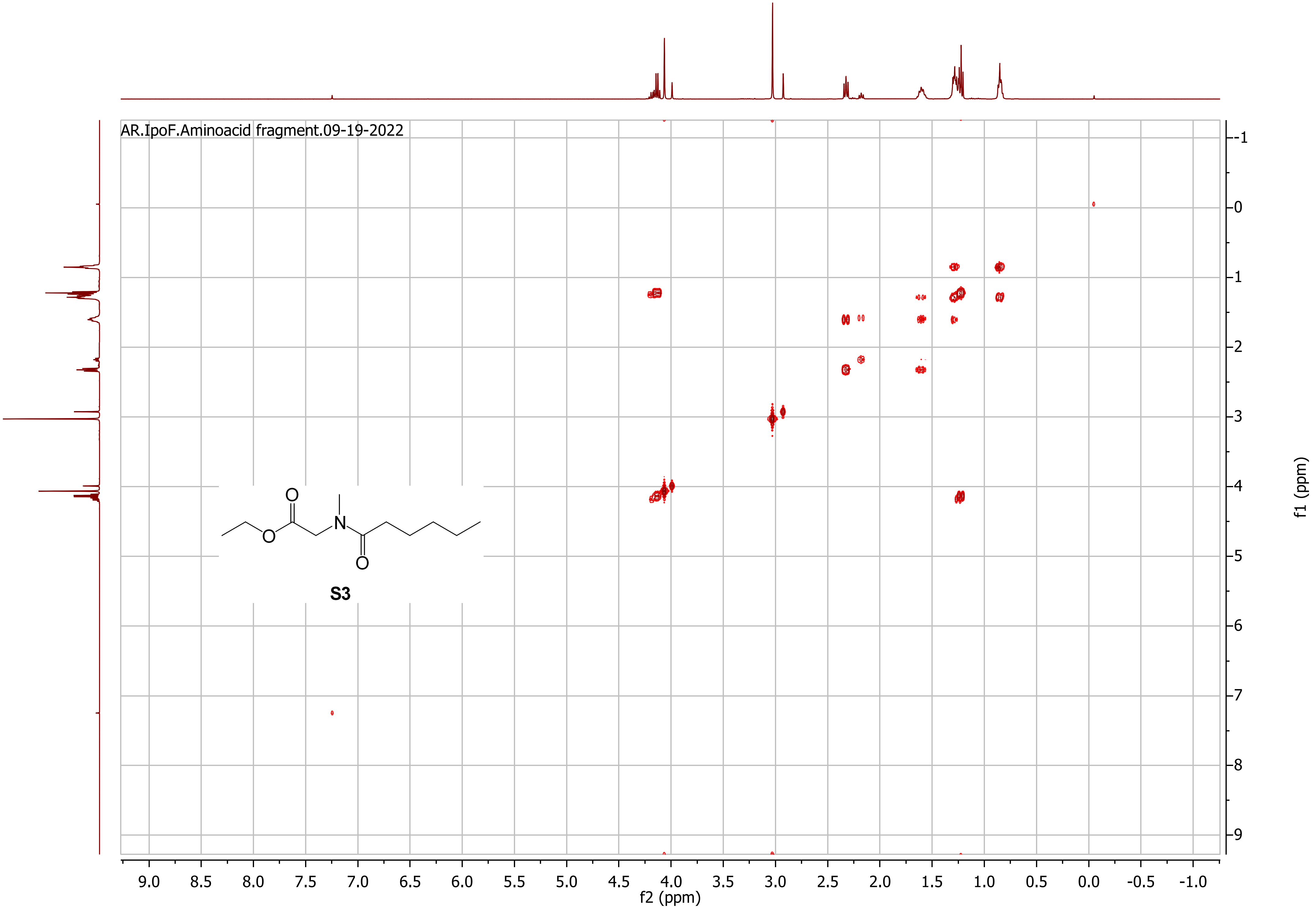
AR.IpoF.Aminoacid fragment.09-19-2022
O
N
O
O
S3
9.0
8.5
8.0
7.5
7.0
6.5
6.0
5.5
5.0
4.5
4.0
3.5
3.0
2.5
2.0
1.5
1.0
0.5
0.0
-0.5
-1.0
f2 (ppm)
-1
0
1
2
3
4
5
6
7
8
9
f1 (ppm)

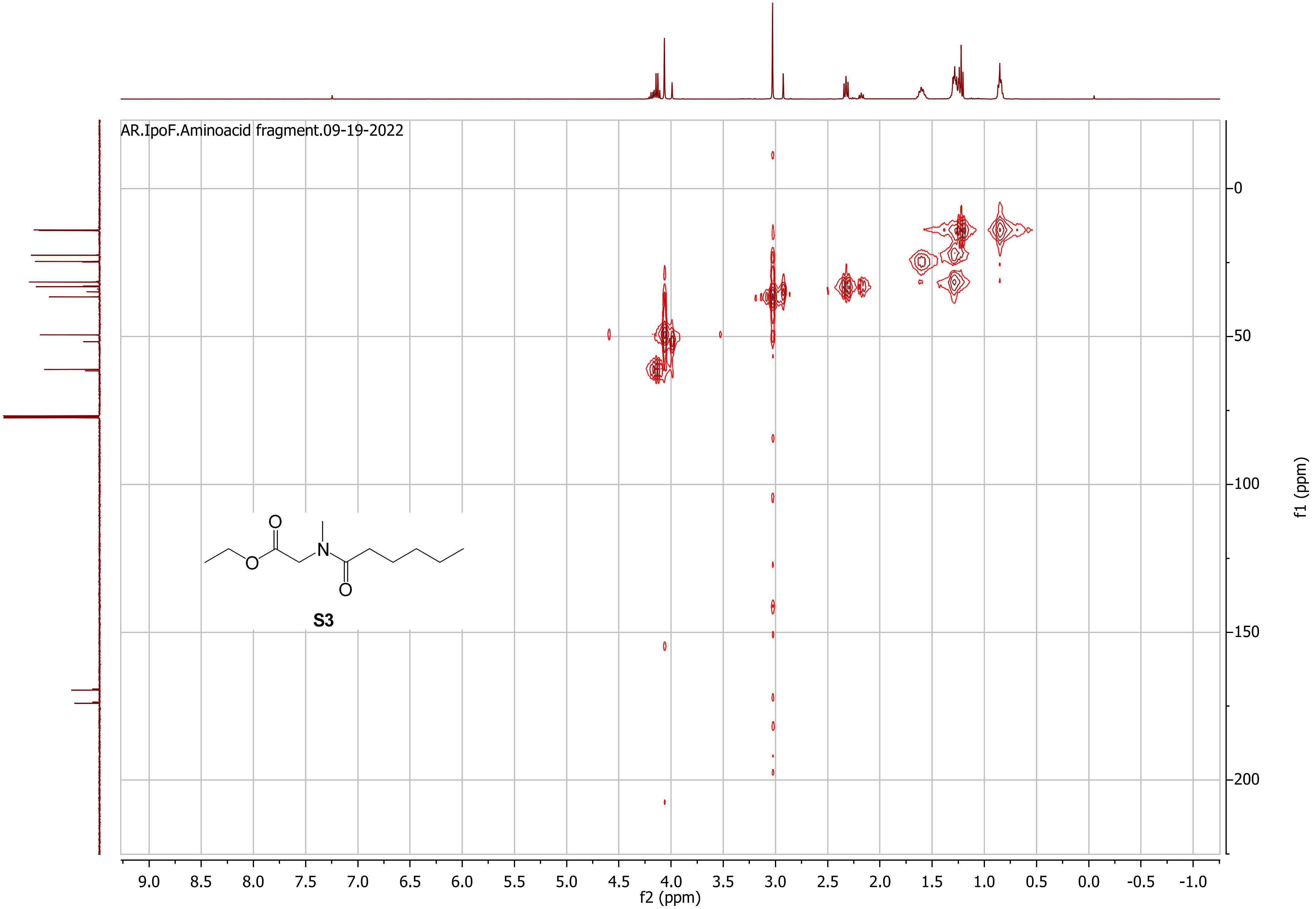
AR.IpoF.Aminoacid fragment.09-19-2022
S3
f2 (ppm)
f1 (ppm)

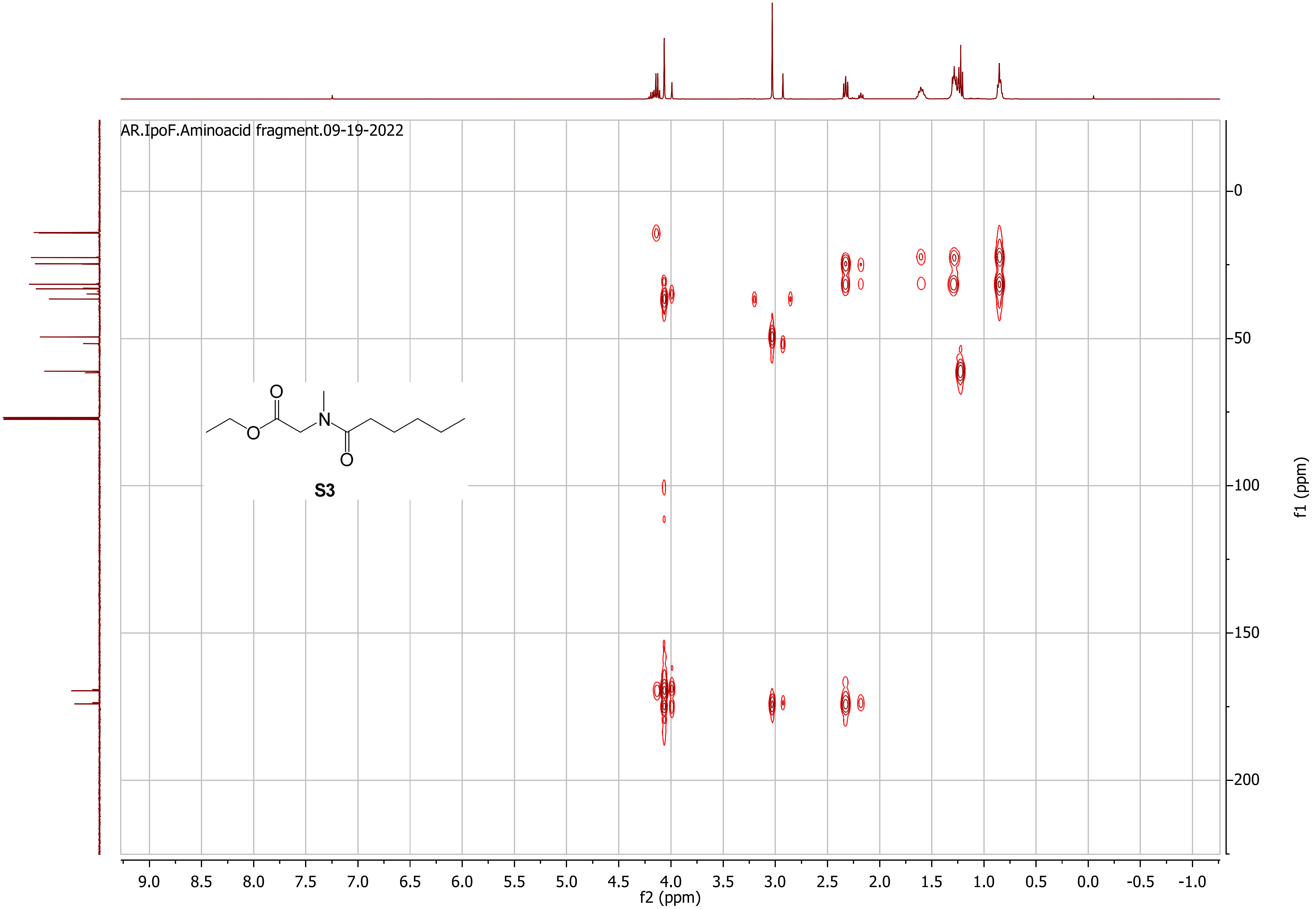

AR.IpoF.Aminoacid fragment.09-19-2022
O
N
O
O
S3
9.0
8.5
8.0
7.5
7.0
6.5
6.0
5.5
5.0
4.5
4.0
3.5
3.0
2.5
2.0
1.5
1.0
0.5
0.0
-0.5
-1.0
f2 (ppm)
0
50
100
150
200
f1 (ppm)

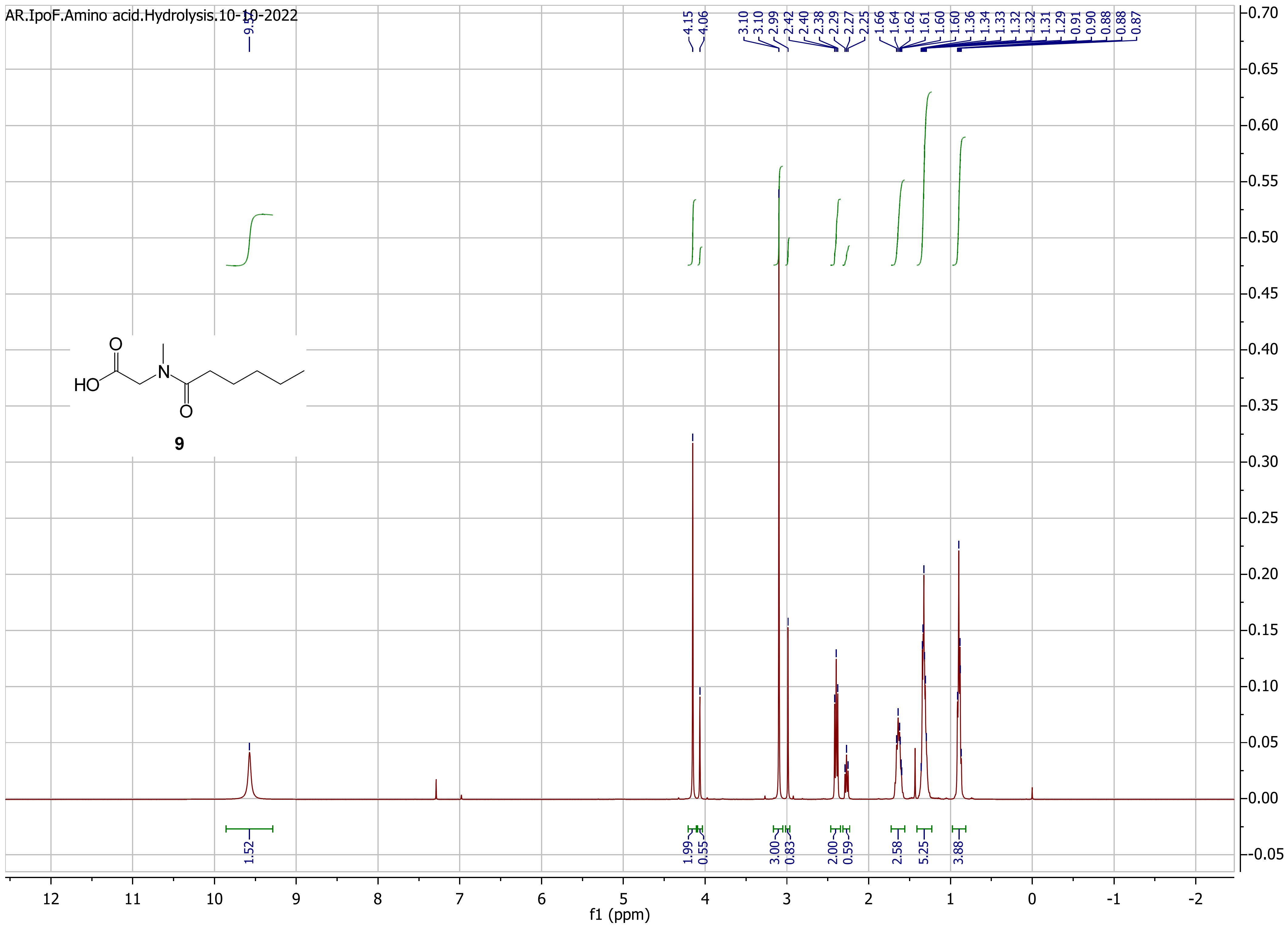

AR.IpoF.Amino acid.Hydrolysis.10-10-2022
9.57
4.15
4.06
3.10
3.10
2.99
2.42
2.40
2.38
2.29
2.27
2.25
1.66
1.64
1.62
1.61
1.60
1.60
1.36
1.34
1.33
1.32
1.32
1.31
1.29
0.91
0.90
0.88
0.88
0.87
HO
O
N
O
9
1.52
1.99
0.55
3.00
0.83
2.00
0.59
2.58
5.25
3.88
f1 (ppm)

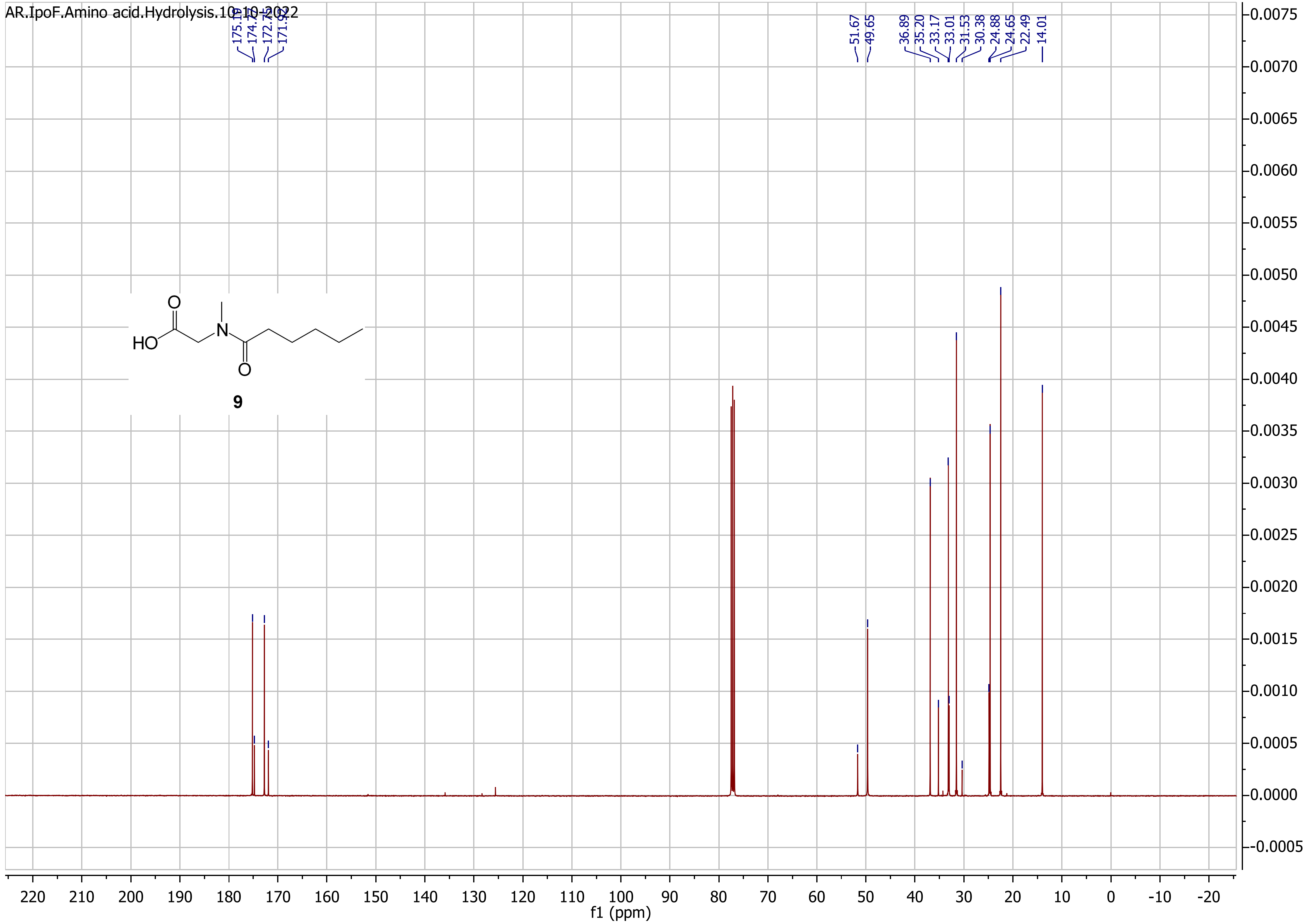

AR.IpoF.Amino acid.Hydrolysis.10-10-2022
175.19
174.77
172.75
171.92
51.67
49.65
36.89
35.20
33.17
33.01
31.53
30.38
24.88
24.65
22.49
14.01
HO
O
N
O
9
0.0075
0.0070
0.0065
0.0060
0.0055
0.0050
0.0045
0.0040
0.0035
0.0030
0.0025
0.0020
0.0015
0.0010
0.0005
0.0000
-0.0005
220 210 200 190 180 170 160 150 140 130 120 110 100 90 80 70 60 50 40 30 20 10 0 -10 -20
f1 (ppm)

AR.IpoF.Amino acid.Hydrolysis.10-10-2022

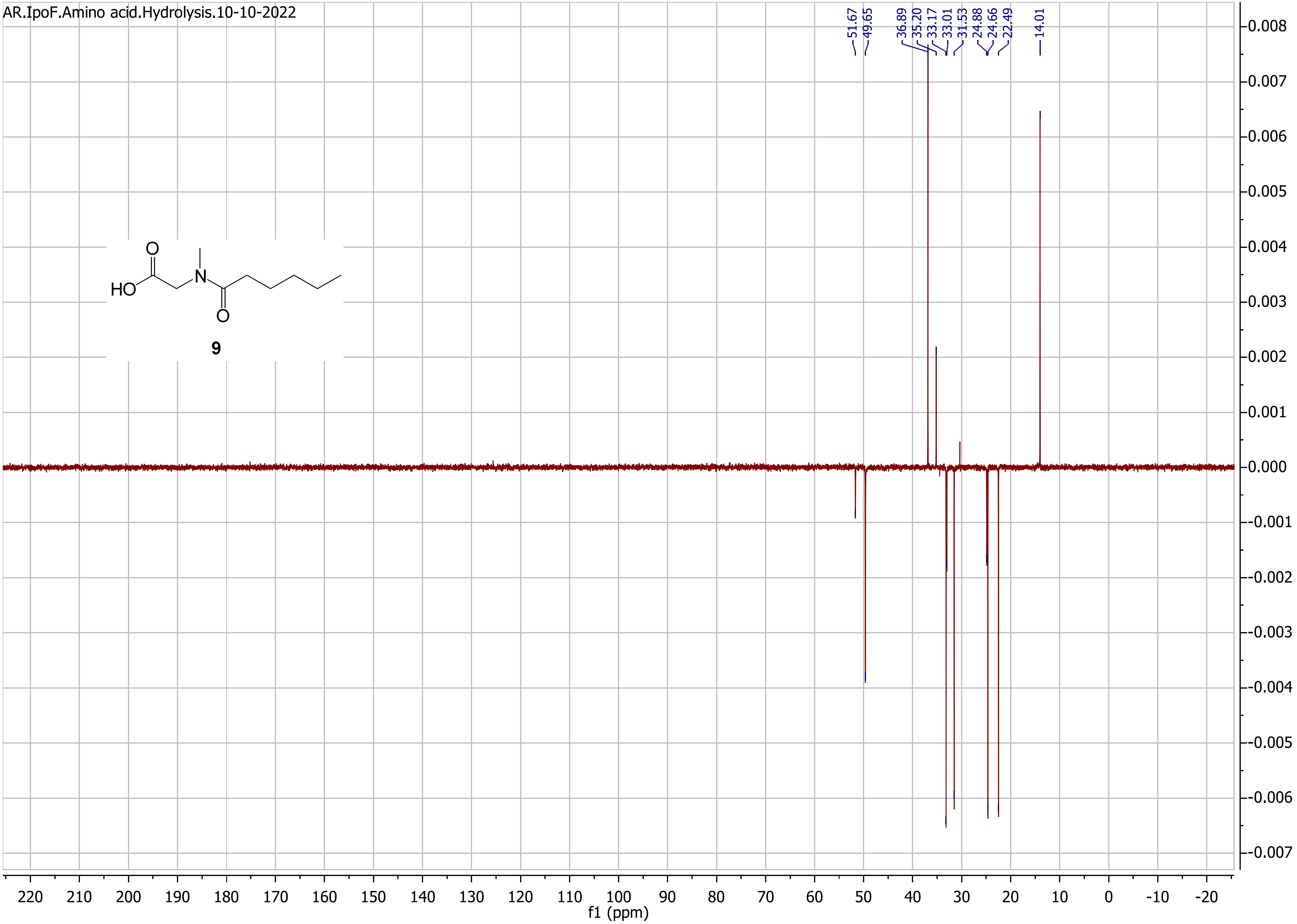

9

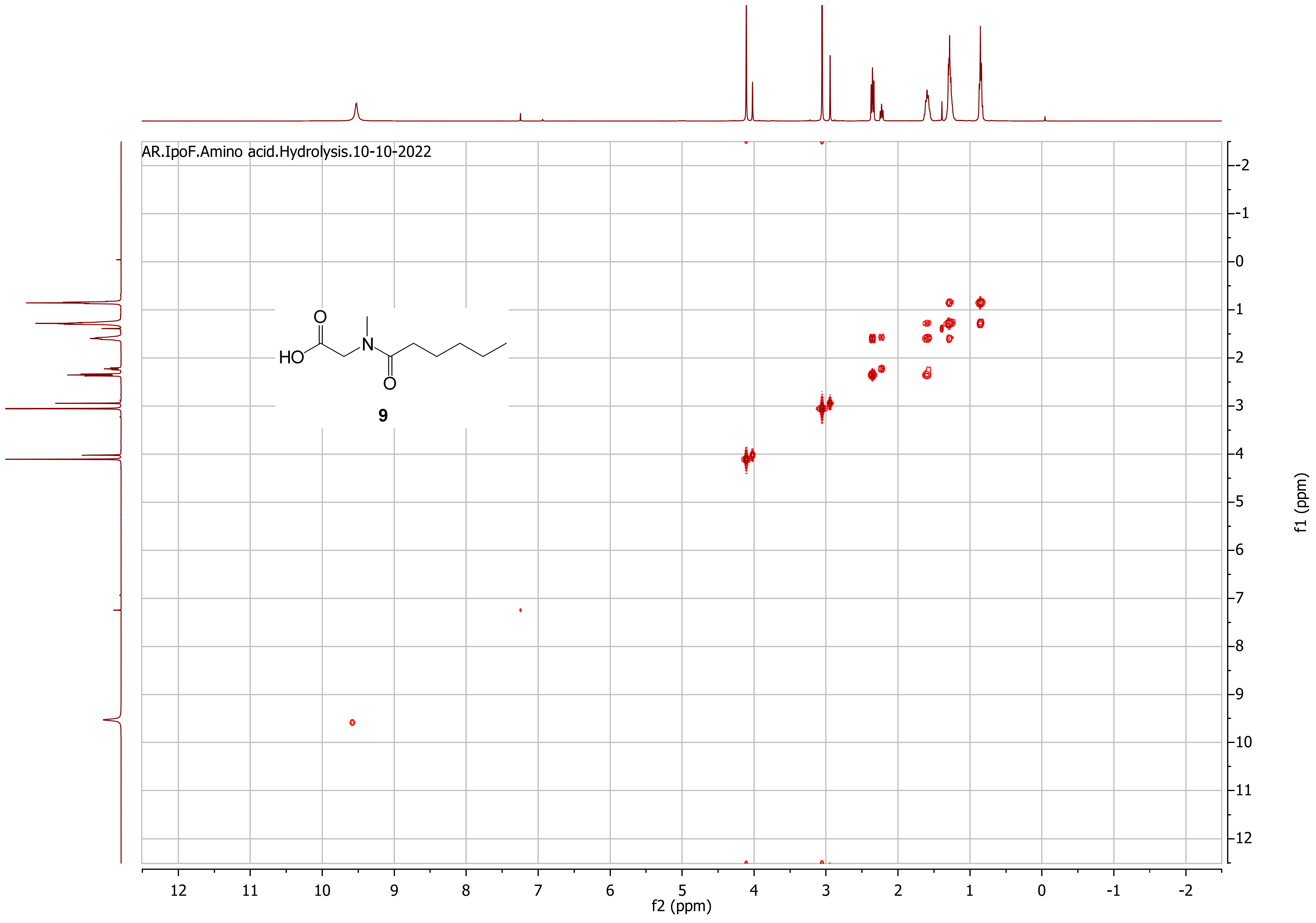
AR.IpoF.Amino acid.Hydrolysis.10-10-2022
HO
O
N
O
9
f2 (ppm)
f1 (ppm)

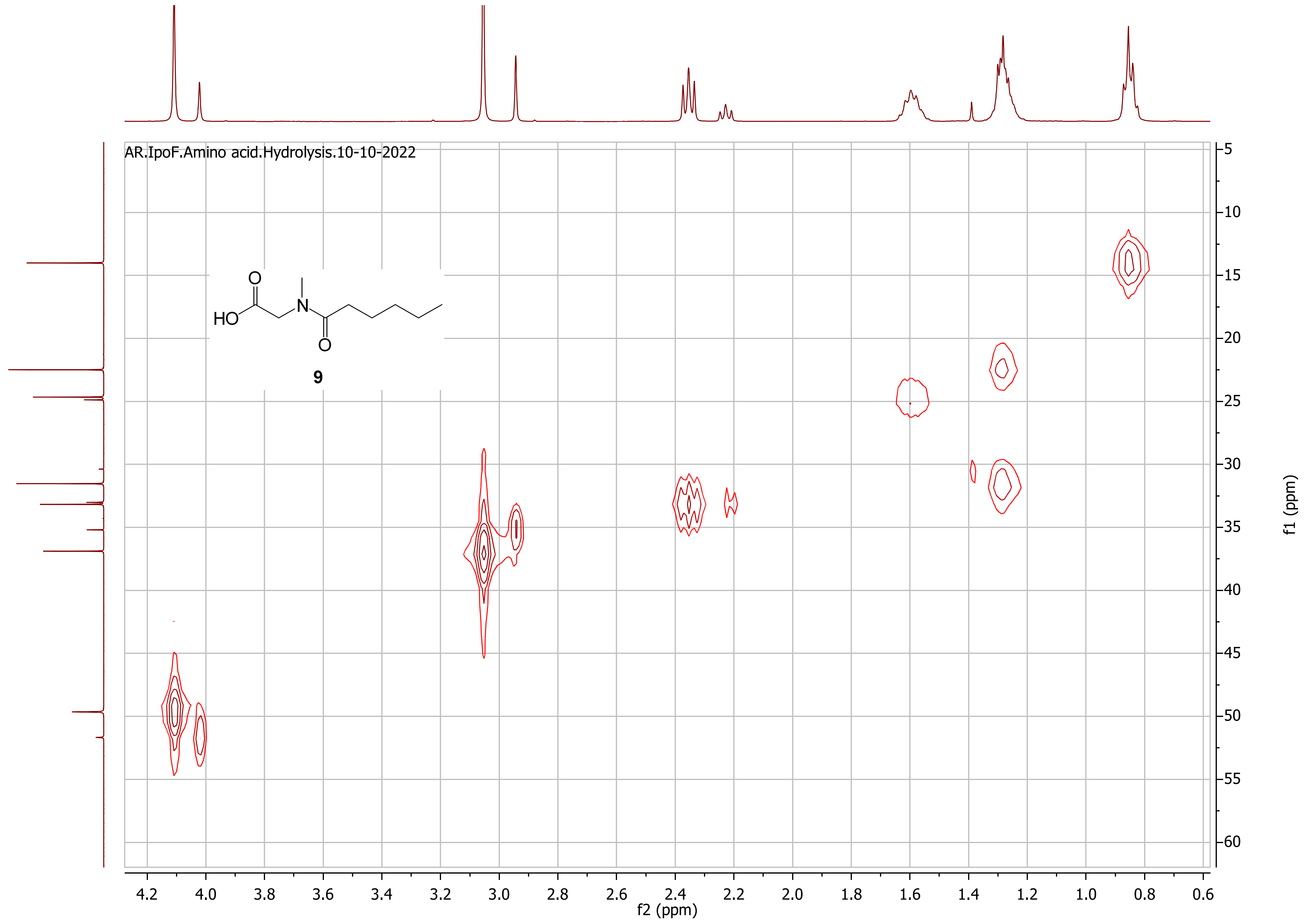

AR.IpoF.Amino acid.Hydrolysis.10-10-2022
HO
O
N
O
9
f2 (ppm)
f1 (ppm)

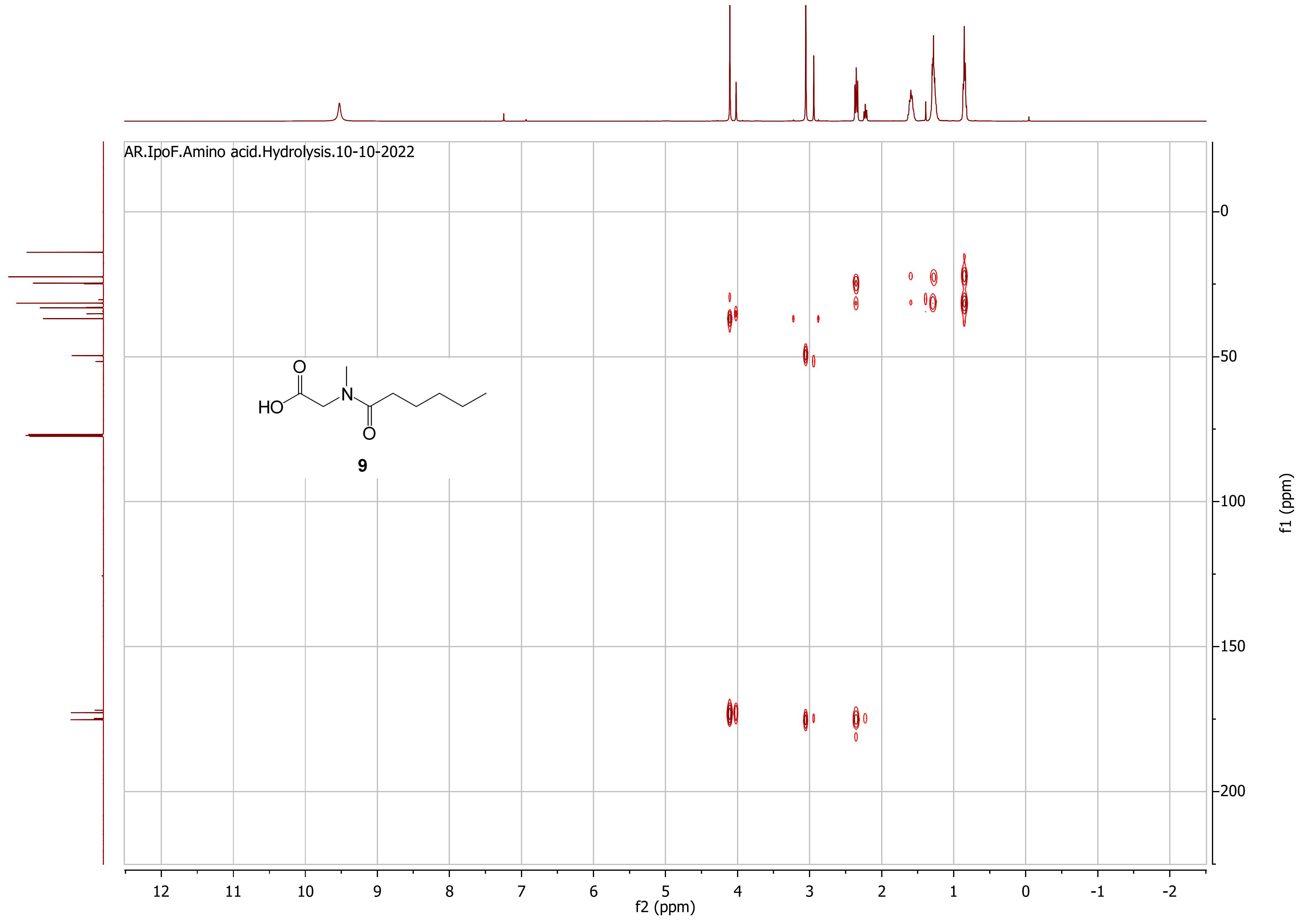
AR.IpoF.Amino acid.Hydrolysis.10-10-2022
HO
O
N
O
9
12
11
10
9
8
7
6
5
4
3
2
1
0
-1
-2
f2 (ppm)
0
50
100
150
200
f1 (ppm)
