# Supplementary material for: Structural Modifications Reveal Dual Functions of the C-4 Carbonyl Group in the Fatty Acid Chain of Ipomoeassin F
Source: Molecules. 2025 Jan 18;30(2):400. doi: 10.3390/molecules30020400 (PMC11767275; doi:10.3390/molecules30020400)
Supplement: Supplementary file 1 [file molecules-30-00400-s001.zip › Supplementary File S2.pdf]

Supplementary Information 2:

Structural Modifications Reveal Dual Functions of the C-4 Carbonyl Group in the Fatty Acid Chain of Ipomoeassin F

Arman Khosravi, Precious Nnamdi, Alexa May, Kelsey Slattery, Robert E. Sammelson and Wei Q. Shi

Table of Contents

#	Item	Pg
1	^{1}H NMR spectrum of **15**	S3
2	^{13}C NMR spectrum of **15**	S4
3	COSY NMR spectrum of **15**	S5
4	HMQC NMR spectrum of **15**	S6
5	HMBC NMR spectrum of **15**	S7
6	^{1}H NMR spectrum of **16**	S8
7	^{13}C NMR spectrum of **16**	S9
8	COSY NMR spectrum of **16**	S10
9	HMQC NMR spectrum of **16**	S11
10	HMBC NMR spectrum of **16**	S12
11	^{1}H NMR spectrum of **17**	S13
12	^{13}C NMR spectrum of **17**	S14
13	COSY NMR spectrum of **17**	S15
14	HMQC NMR spectrum of **17**	S16
15	HMBC NMR spectrum of **17**	S17
16	^{1}H NMR spectrum of **18**	S18
17	^{13}C NMR spectrum of **18**	S19
18	COSY NMR spectrum of **18**	S20
19	HMQC NMR spectrum of **18**	S21
20	HMBC NMR spectrum of **18**	S22
21	^{1}H NMR spectrum of **21**	S23
22	^{13}C NMR spectrum of **21**	S24
23	DEPT 135 NMR spectrum of **21**	S25
24	COSY NMR spectrum of **21**	S26
25	HMQC NMR spectrum of **21**	S27
26	HMBC NMR spectrum of **21**	S28

#	Item	Pg
27	^{1}H NMR spectrum of **22**	S29
28	^{13}C NMR spectrum of **22**	S30
29	DEPT 135 NMR spectrum of **22**	S31
30	COSY NMR spectrum of **22**	S32
31	HMQC NMR spectrum of **22**	S33
32	HMBC NMR spectrum of **22**	S34
33	^{1}H NMR spectrum of **23**	S35
34	^{13}C NMR spectrum of **23**	S36
35	DEPT 135 NMR spectrum of **23**	S37
36	COSY NMR spectrum of **23**	S38
37	HMQC NMR spectrum of **23**	S39
38	HMBC NMR spectrum of **23**	S40
39	^{1}H NMR spectrum of **10**	S41
40	^{13}C NMR spectrum of **10**	S42
41	DEPT 135 NMR spectrum of **10**	S43
42	COSY NMR spectrum of **10**	S44
43	HMQC NMR spectrum of **10**	S45
44	HMBC NMR spectrum of **10**	S46
45	^{1}H NMR spectrum of **11**	S47
46	^{13}C NMR spectrum of **11**	S48
47	DEPT 135 NMR spectrum of **11**	S49
48	COSY NMR spectrum of **11**	S50
49	HMQC NMR spectrum of **11**	S51
50	HMBC NMR spectrum of **11**	S52

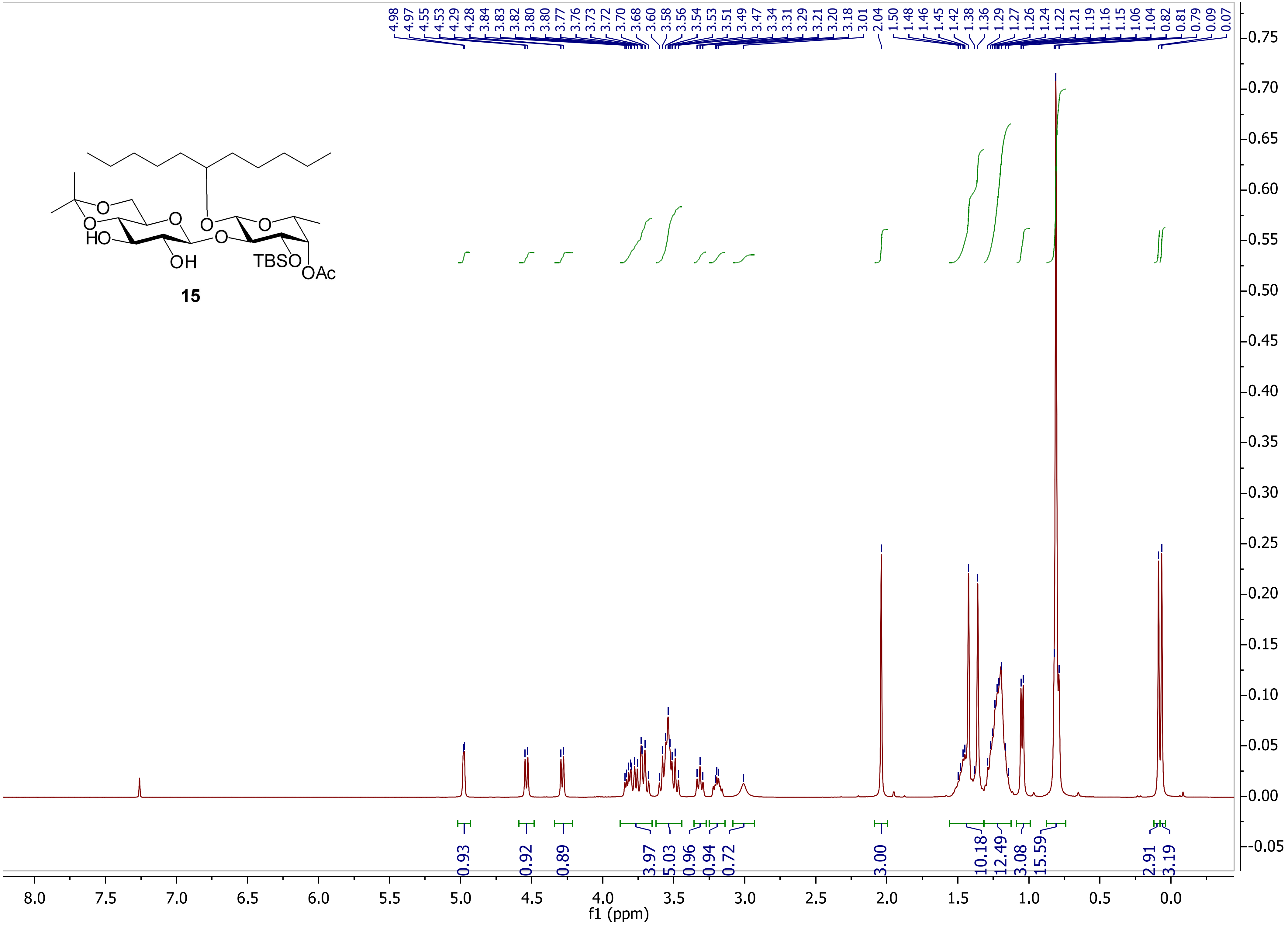
HO
OH
TBSO
OAc
15
f1 (ppm)

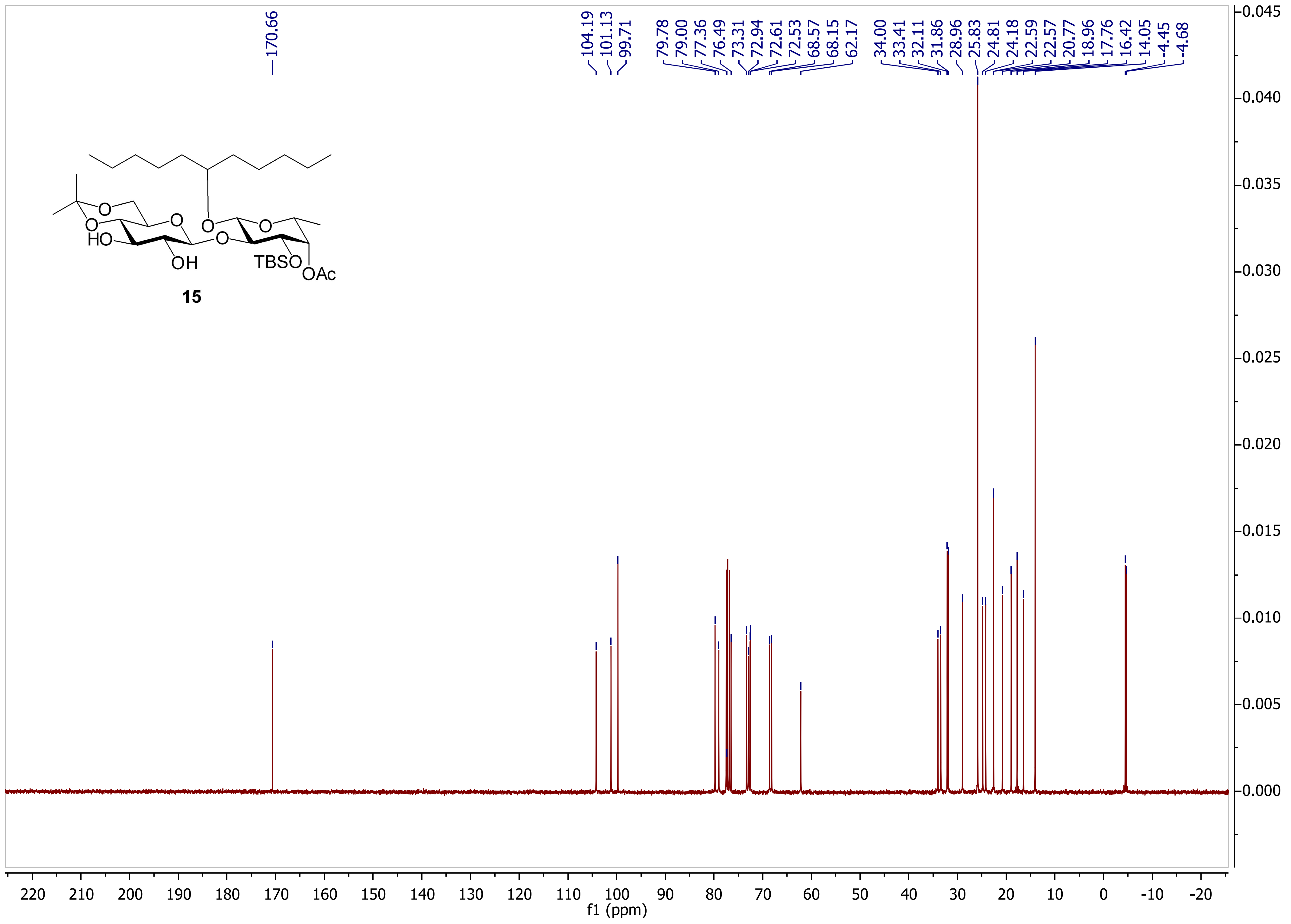
170.66
104.19
101.13
99.71
79.78
79.00
77.36
76.49
73.31
72.94
72.61
72.53
68.57
68.15
62.17
34.00
33.41
32.11
31.86
28.96
25.83
24.81
24.18
22.59
22.57
20.77
18.96
17.76
16.42
14.05
-4.45
-4.68
HO
OH
O
TBSO
OAc
15
0.045
0.040
0.035
0.030
0.025
0.020
0.015
0.010
0.005
0.000
220 210 200 190 180 170 160 150 140 130 120 110 100 90 80 70 60 50 40 30 20 10 0 -10 -20
f1 (ppm)

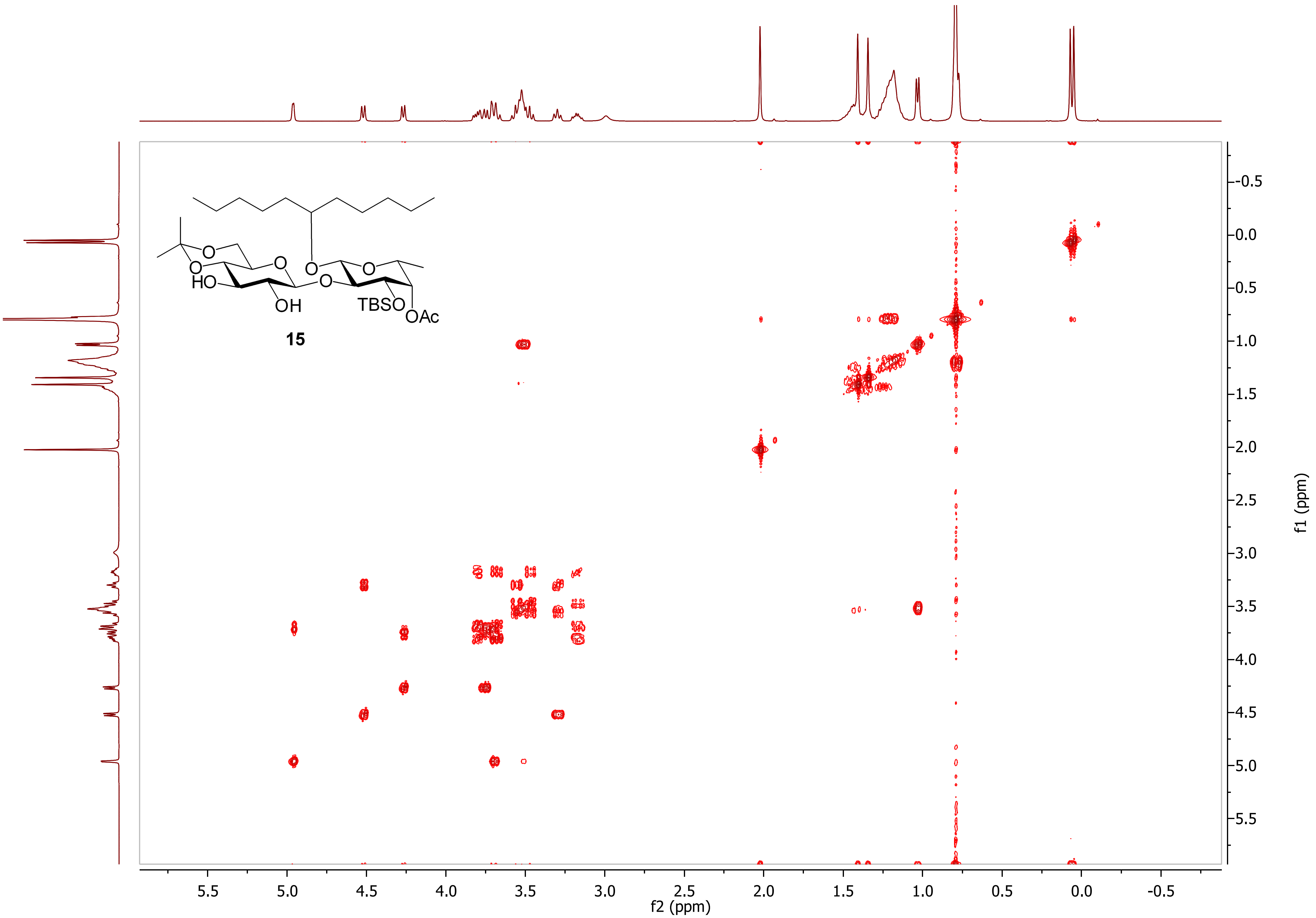

HO
OH
TBSO
OAc
15
f2 (ppm)
f1 (ppm)

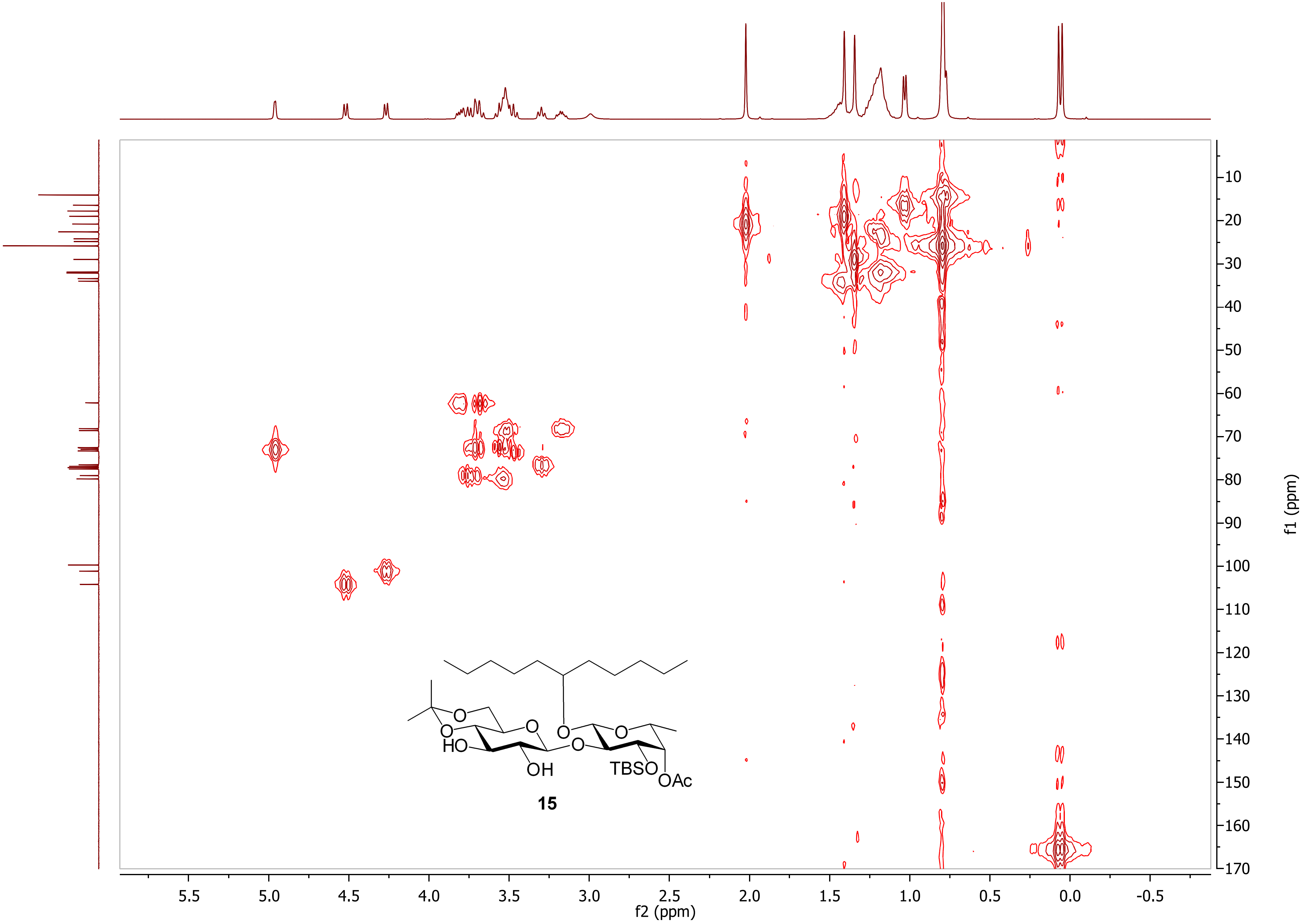

f2 (ppm)
f1 (ppm)
HO
OH
TBSO
OAc
15

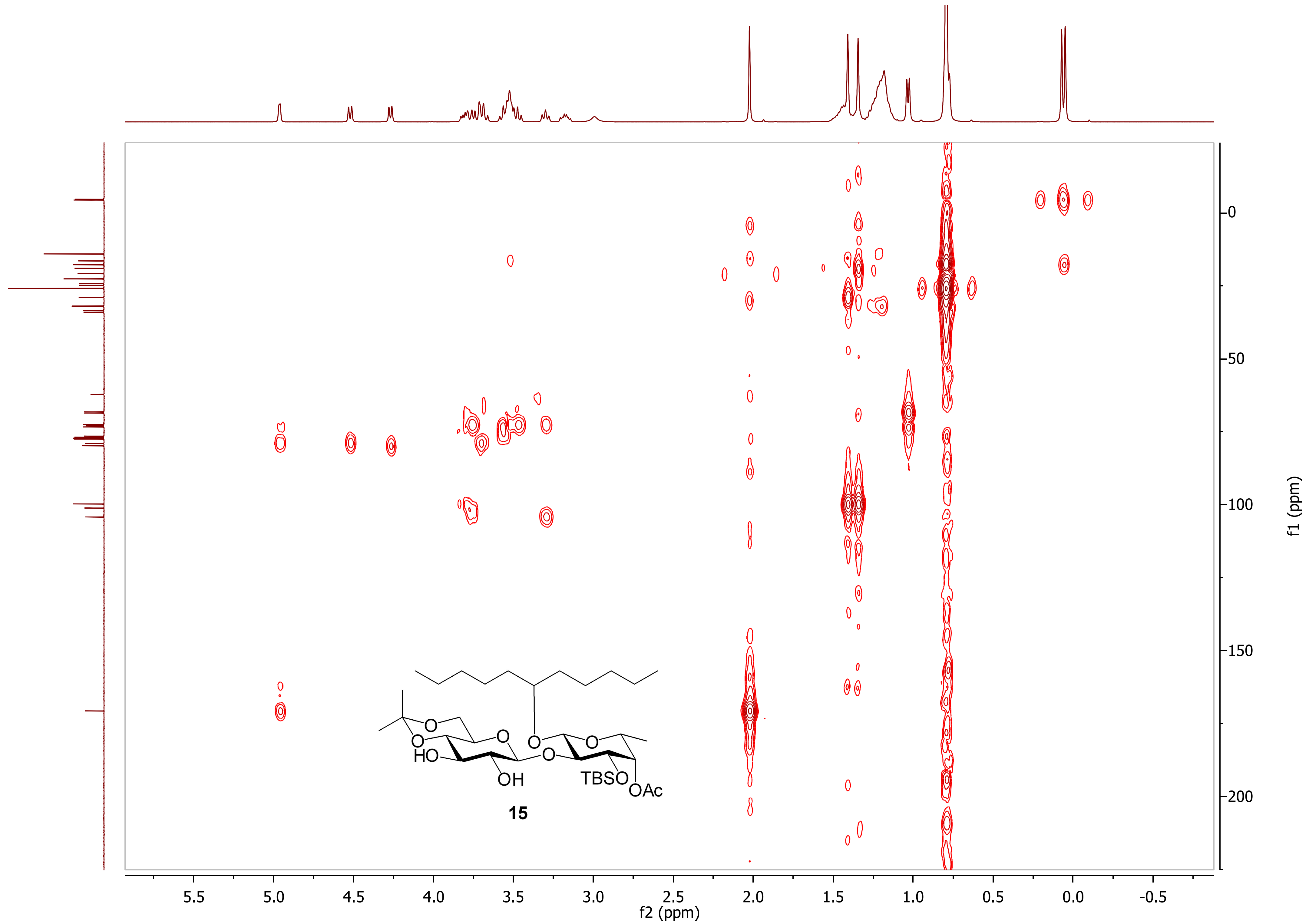

5.5
5.0
4.5
4.0
3.5
3.0
2.5
2.0
1.5
1.0
0.5
0.0
-0.5
f2 (ppm)
0
50
100
150
200
f1 (ppm)
O
O
HO
O
OH
O
O
O
TBSO
OAc
15

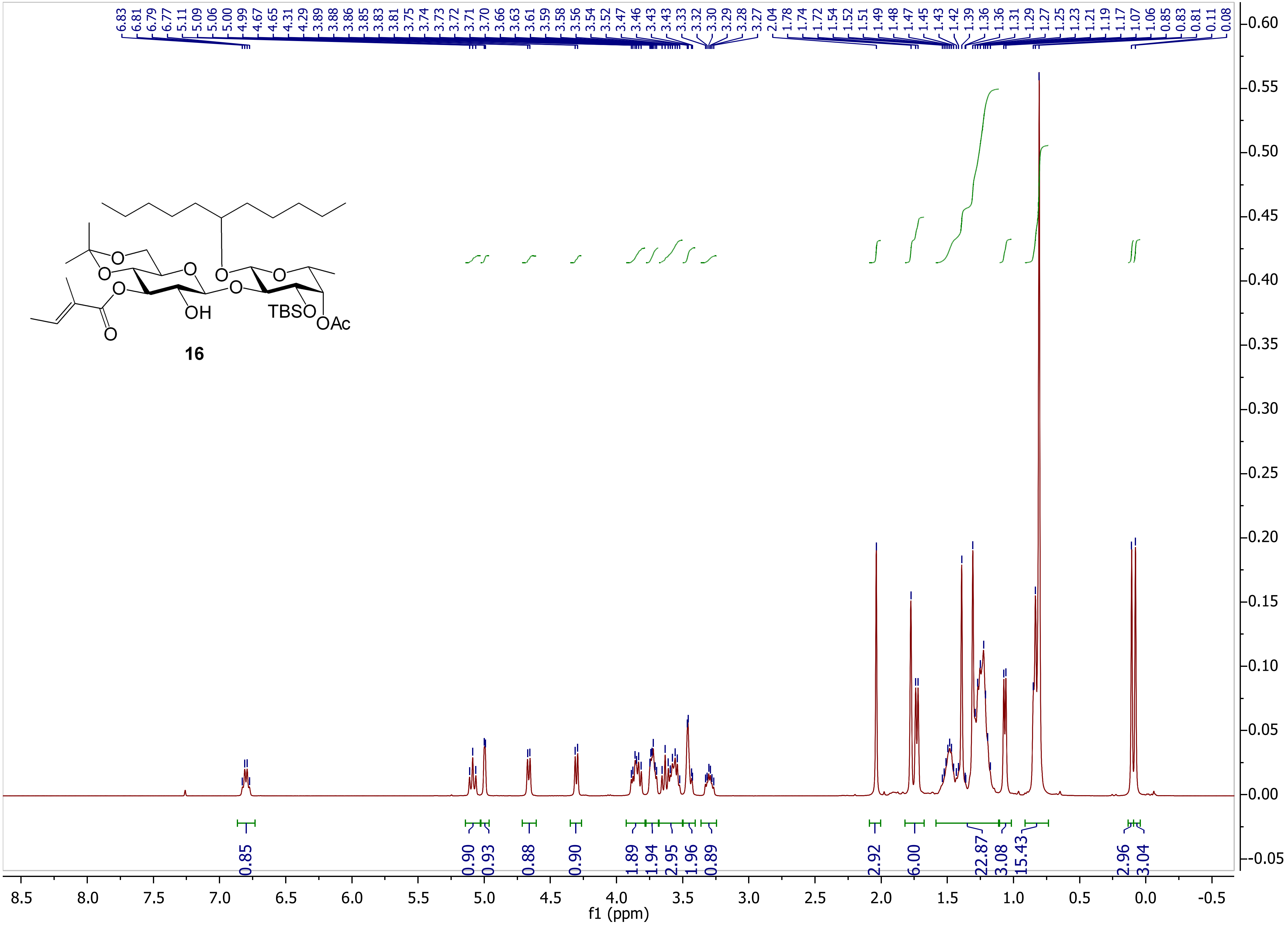

6.83 6.81 6.79 6.77 5.11 5.09 5.06 5.00 4.99 4.67 4.65 4.31 4.29 3.89 3.88 3.86 3.85 3.83 3.81 3.75 3.74 3.73 3.72 3.71 3.70 3.66 3.63 3.61 3.59 3.58 3.56 3.54 3.52 3.47 3.46 3.43 3.43 3.33 3.32 3.30 3.29 3.28 3.27 2.04 1.78 1.74 1.72 1.54 1.52 1.51 1.49 1.48 1.47 1.45 1.43 1.42 1.39 1.36 1.36 1.31 1.29 1.27 1.25 1.23 1.21 1.19 1.17 1.07 1.06 0.85 0.83 0.81 0.11 0.08
OH
TBSO
OAc
16
0.85 0.90 0.93 0.88 0.90 1.89 1.94 2.95 1.96 0.89 2.92 6.00 22.87 3.08 15.43 2.96 3.04
f1 (ppm)

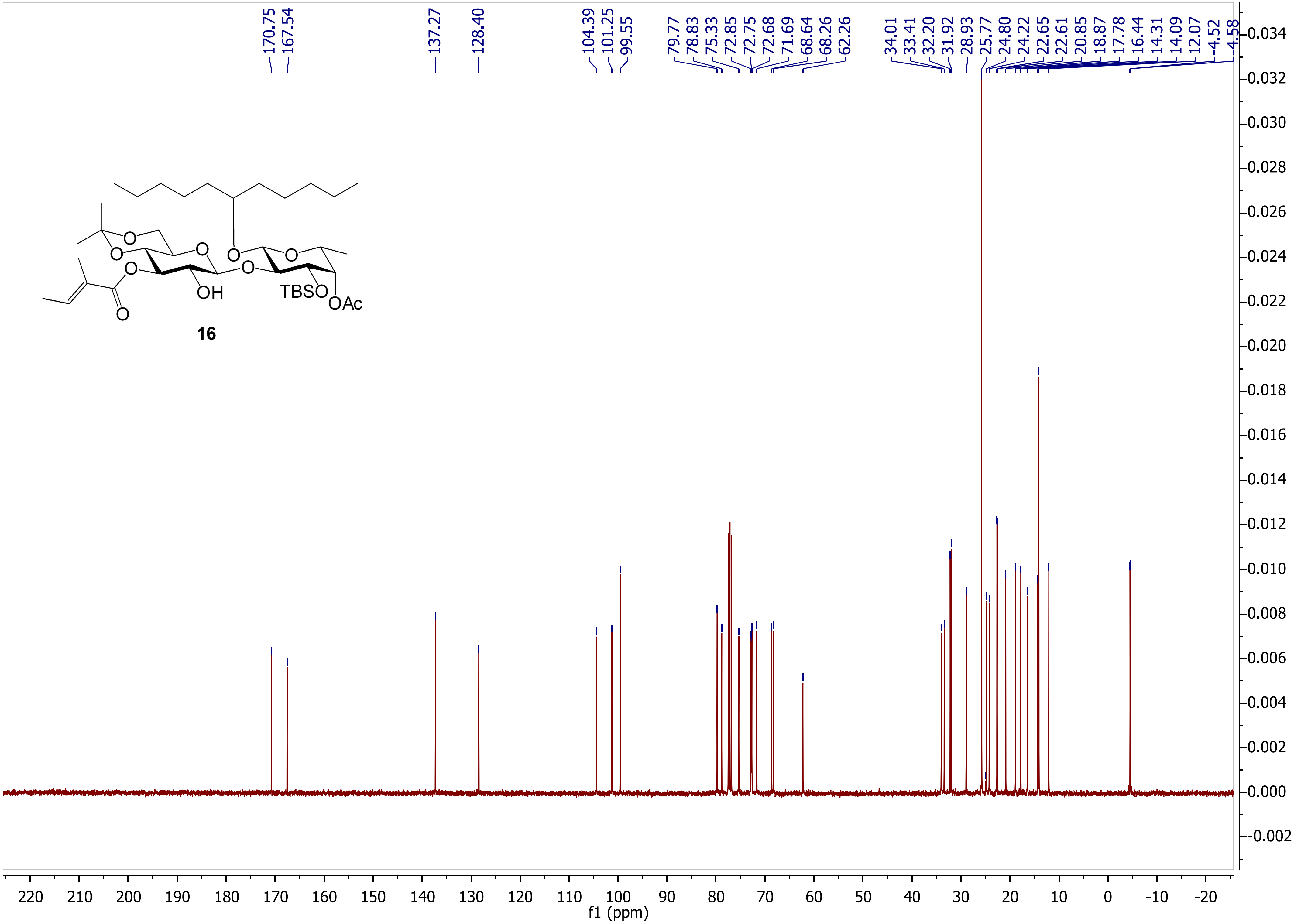
170.75
167.54
137.27
128.40
104.39
101.25
99.55
79.77
78.83
75.33
72.85
72.75
72.68
71.69
68.64
68.26
62.26
34.01
33.41
32.20
31.92
28.93
25.77
24.80
24.22
22.65
22.61
20.85
18.87
17.78
16.44
14.31
14.09
12.07
-4.52
-4.58
OH
TBSO
OAc
16
f1 (ppm)

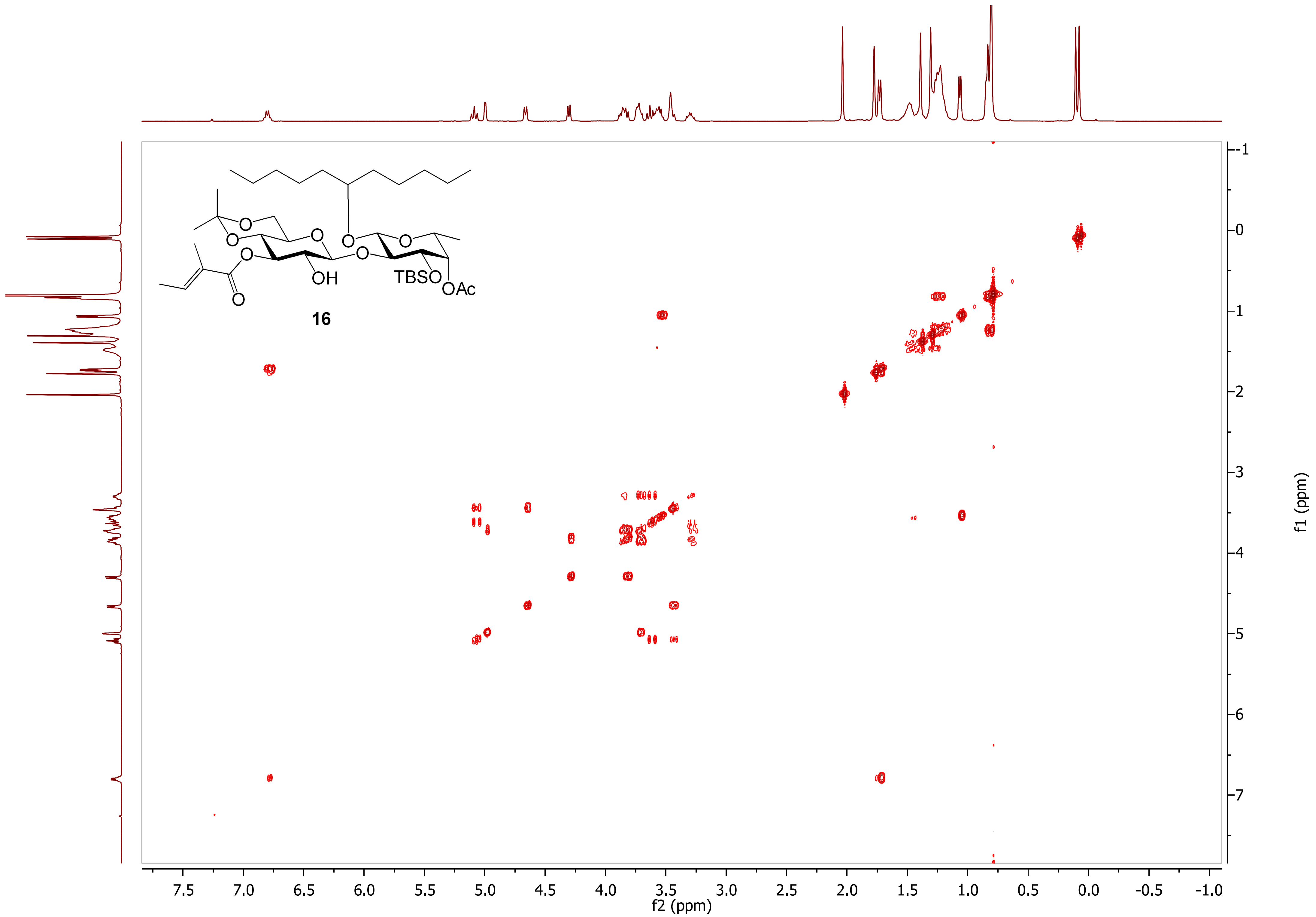

O
O
O
O
O
O
O
OH
TBSO
OAc
O
16
7.5
7.0
6.5
6.0
5.5
5.0
4.5
4.0
3.5
3.0
2.5
2.0
1.5
1.0
0.5
0.0
-0.5
-1.0
f2 (ppm)
-1
0
1
2
3
4
5
6
7
f1 (ppm)

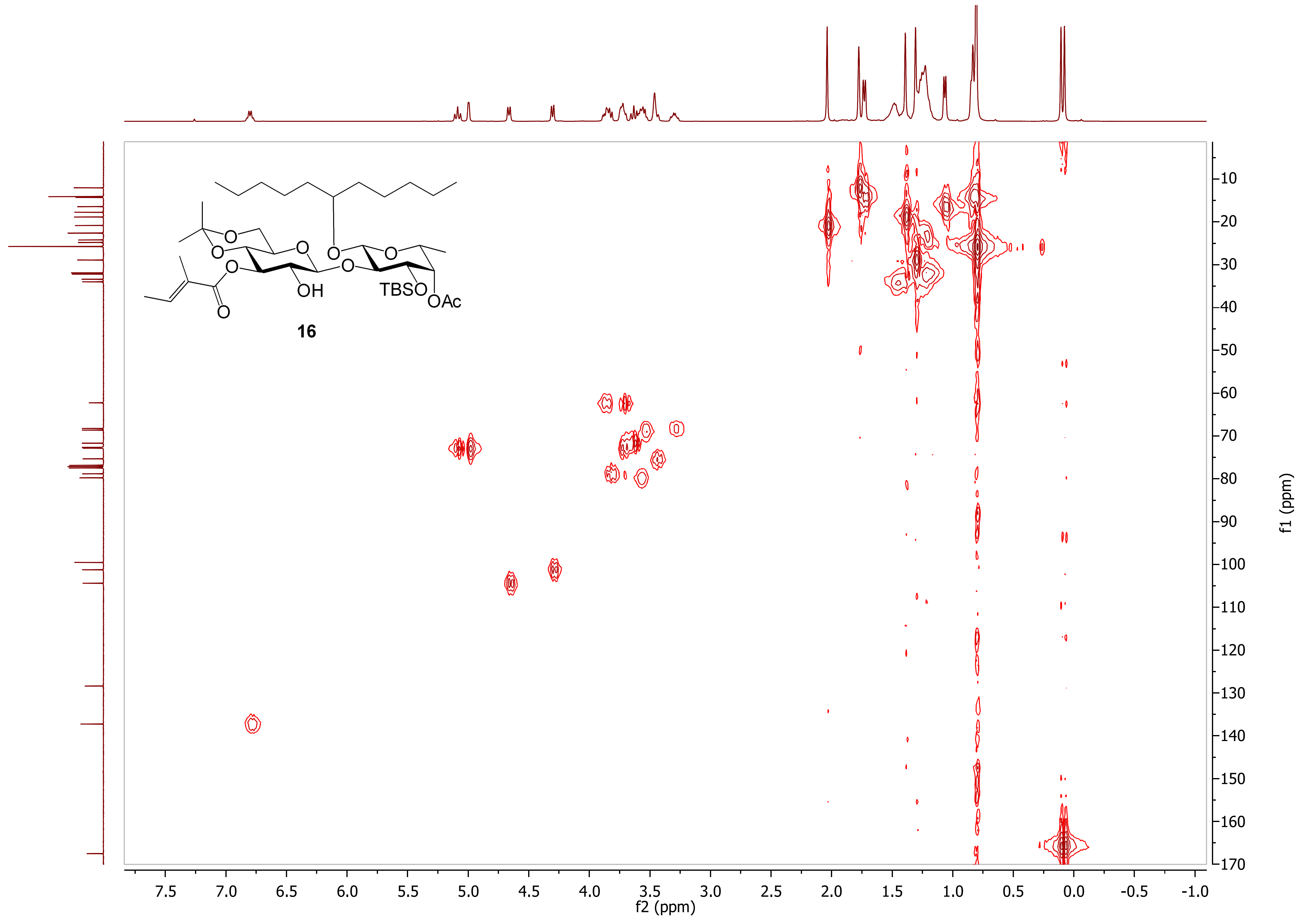

TBSO
OH
OAc
16
f2 (ppm)
f1 (ppm)

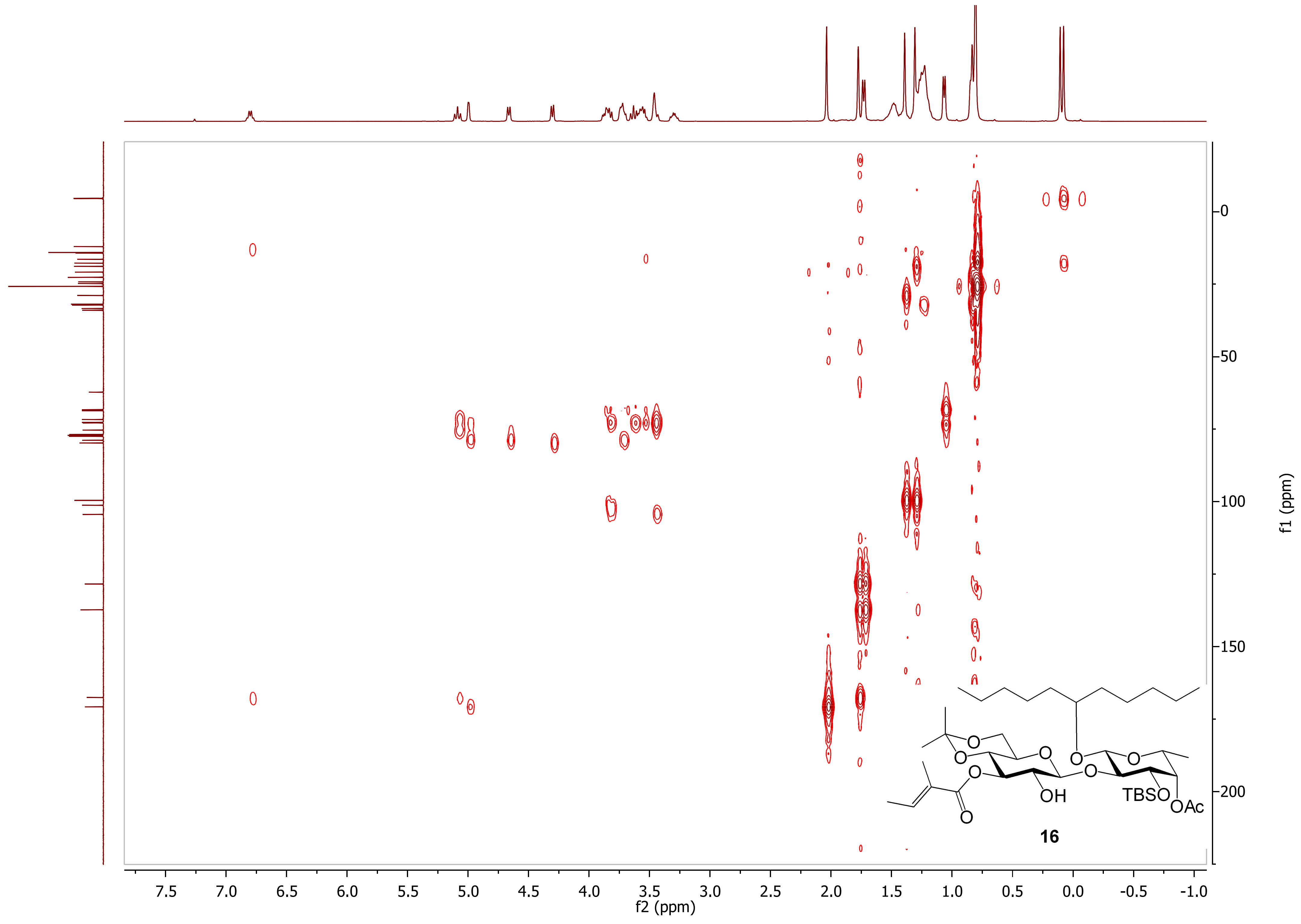

f2 (ppm)
f1 (ppm)
TBSO
OAc
OH
16

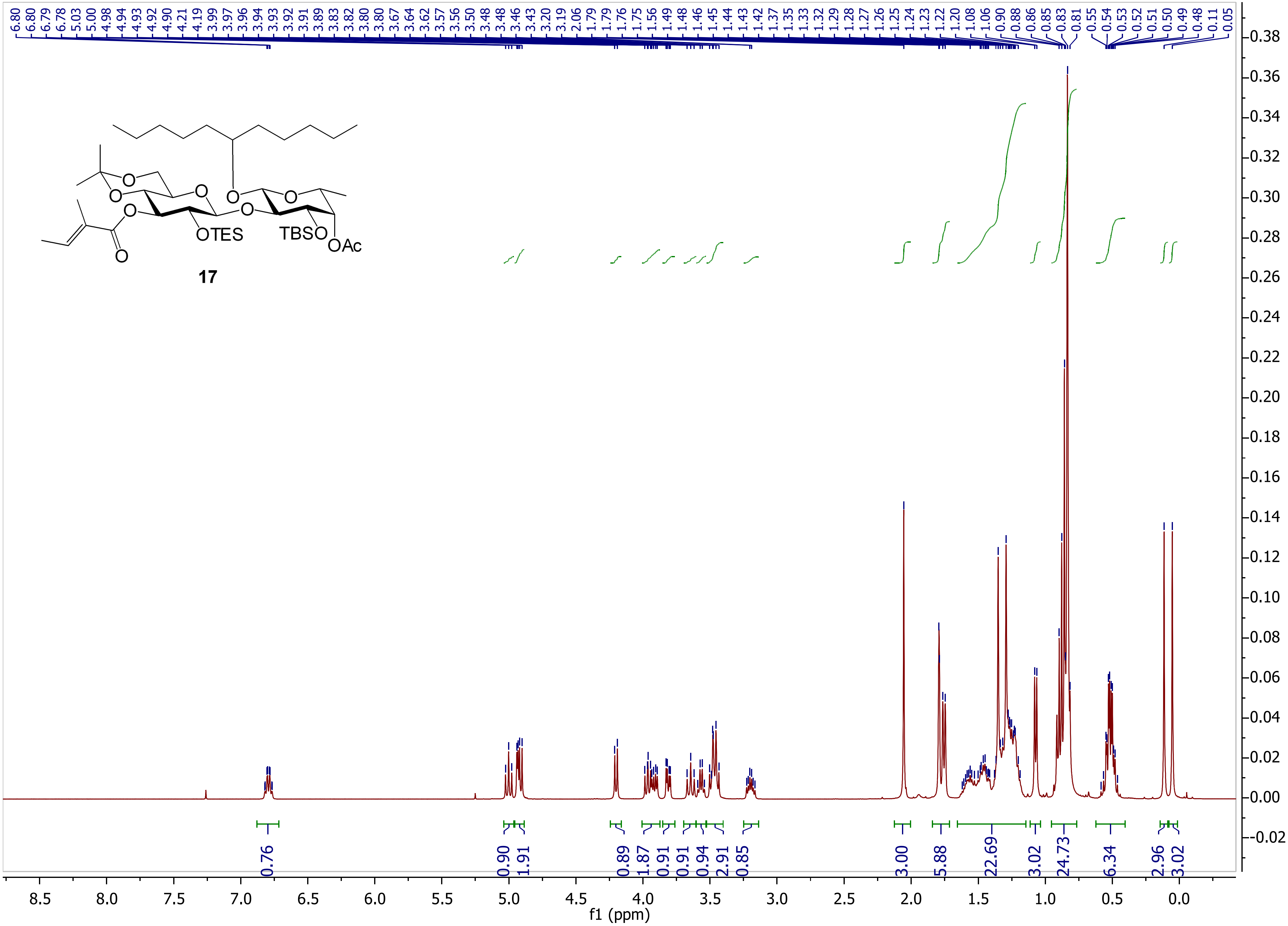
6.80 6.80 6.79 6.78 5.03 5.00 4.98 4.94 4.93 4.92 4.90 4.21 4.19 3.99 3.97 3.96 3.94 3.93 3.92 3.91 3.89 3.83 3.82 3.80 3.80 3.67 3.64 3.62 3.57 3.56 3.50 3.48 3.48 3.46 3.43 3.20 3.19 2.06 1.79 1.79 1.76 1.75 1.56 1.49 1.48 1.46 1.45 1.44 1.43 1.42 1.37 1.35 1.33 1.32 1.29 1.28 1.27 1.26 1.25 1.24 1.23 1.22 1.20 1.08 1.06 0.90 0.88 0.86 0.85 0.83 0.81 0.55 0.54 0.53 0.52 0.51 0.50 0.49 0.48 0.11 0.05
OTES
TBSO
OAc
17
0.76 0.90 1.91 0.89 1.87 0.91 0.91 0.94 2.91 0.85 3.00 5.88 22.69 3.02 24.73 6.34 2.96 3.02
8.5 8.0 7.5 7.0 6.5 6.0 5.5 5.0 4.5 4.0 3.5 3.0 2.5 2.0 1.5 1.0 0.5 0.0
f1 (ppm)
0.38 0.36 0.34 0.32 0.30 0.28 0.26 0.24 0.22 0.20 0.18 0.16 0.14 0.12 0.10 0.08 0.06 0.04 0.02 0.00 -0.02

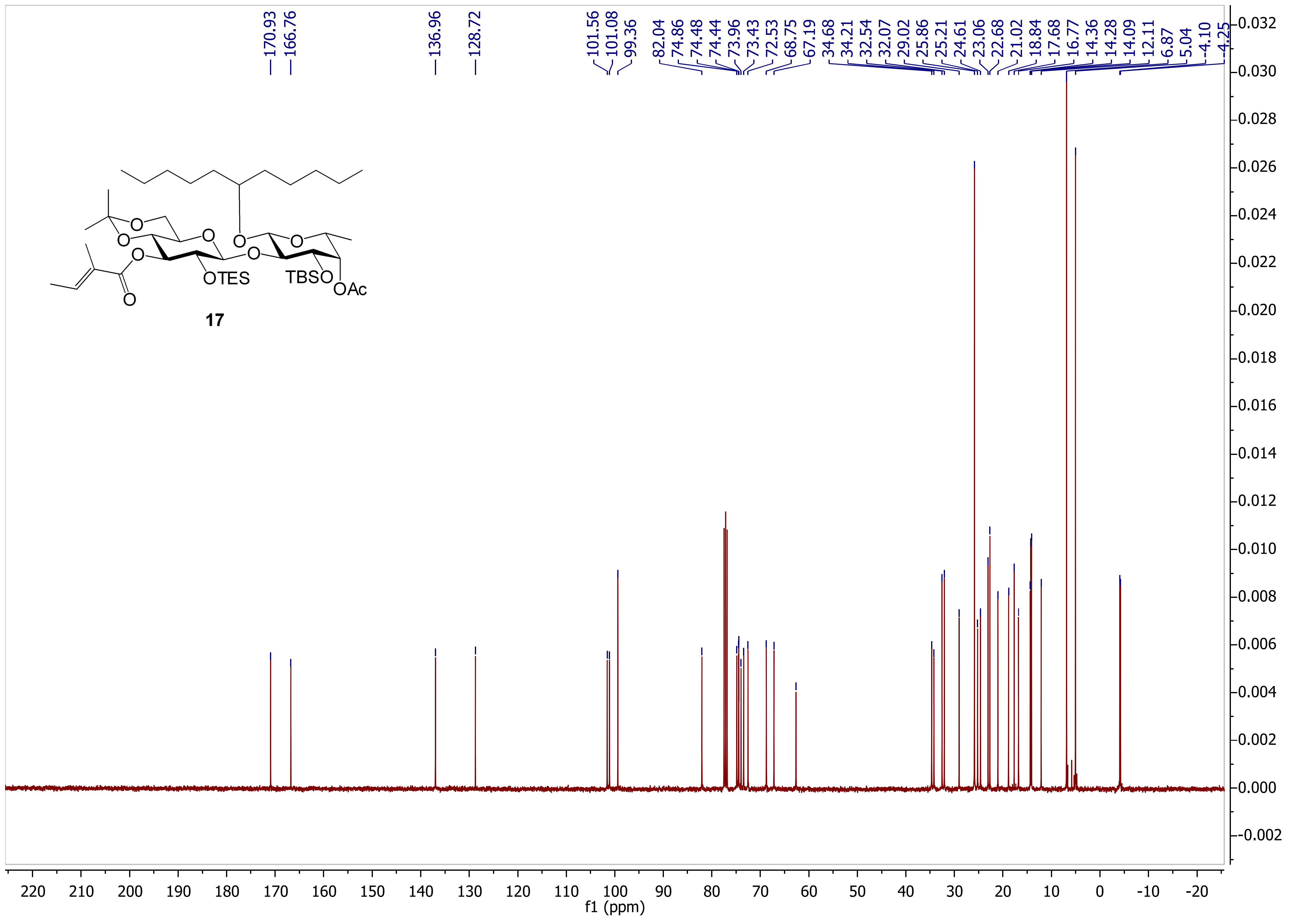
170.93
166.76
136.96
128.72
101.56
101.08
99.36
82.04
74.86
74.48
74.44
73.96
73.43
72.53
68.75
67.19
34.68
34.21
32.54
32.07
29.02
25.86
25.21
24.61
23.06
22.68
21.02
18.84
17.68
16.77
14.36
14.28
14.09
12.11
6.87
5.04
-4.10
-4.25
O
O
O
O
O
O
OTES
TBSO
OAc
O
17
f1 (ppm)

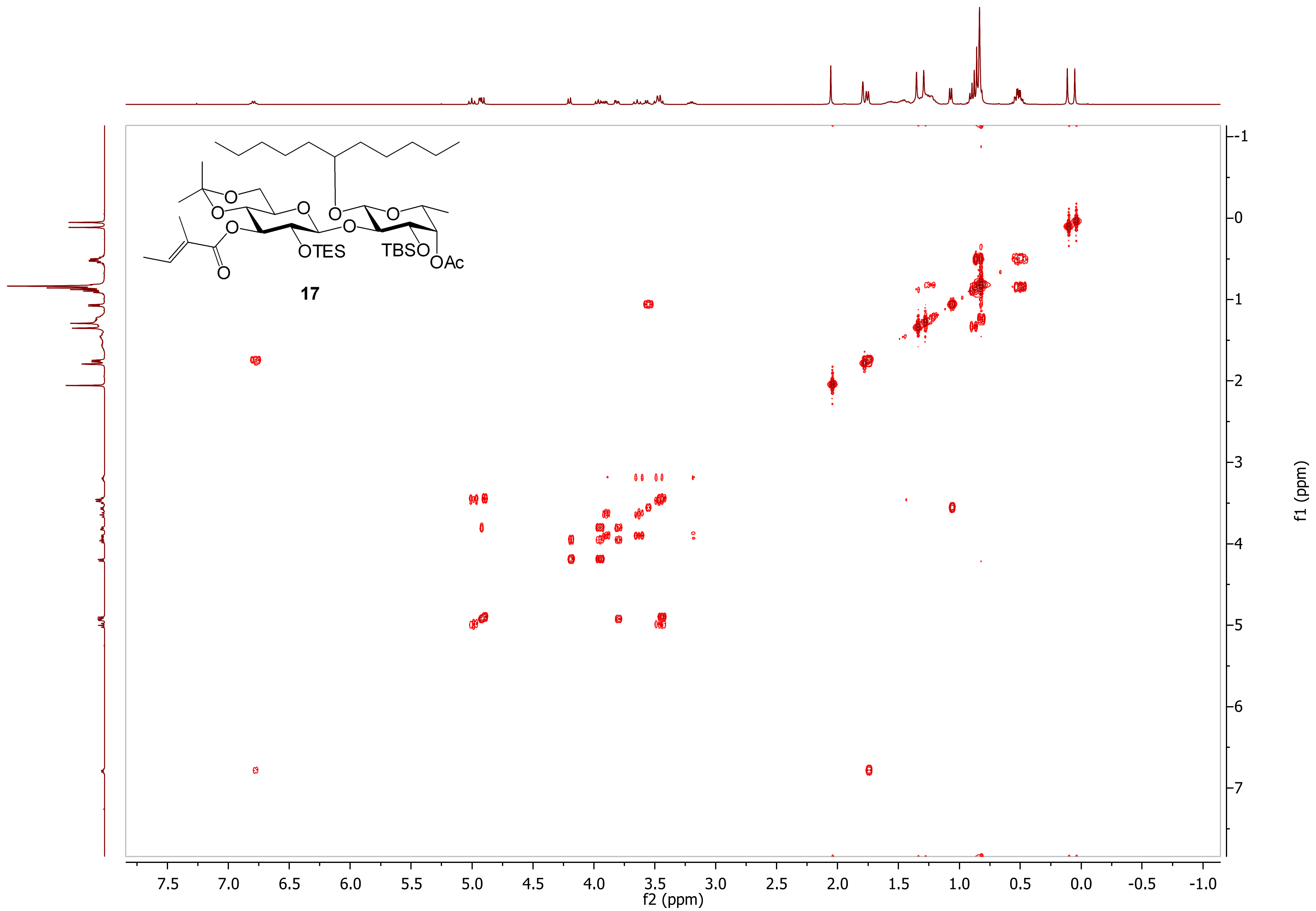

OTES
TBSO
OAc
17
f2 (ppm)
f1 (ppm)

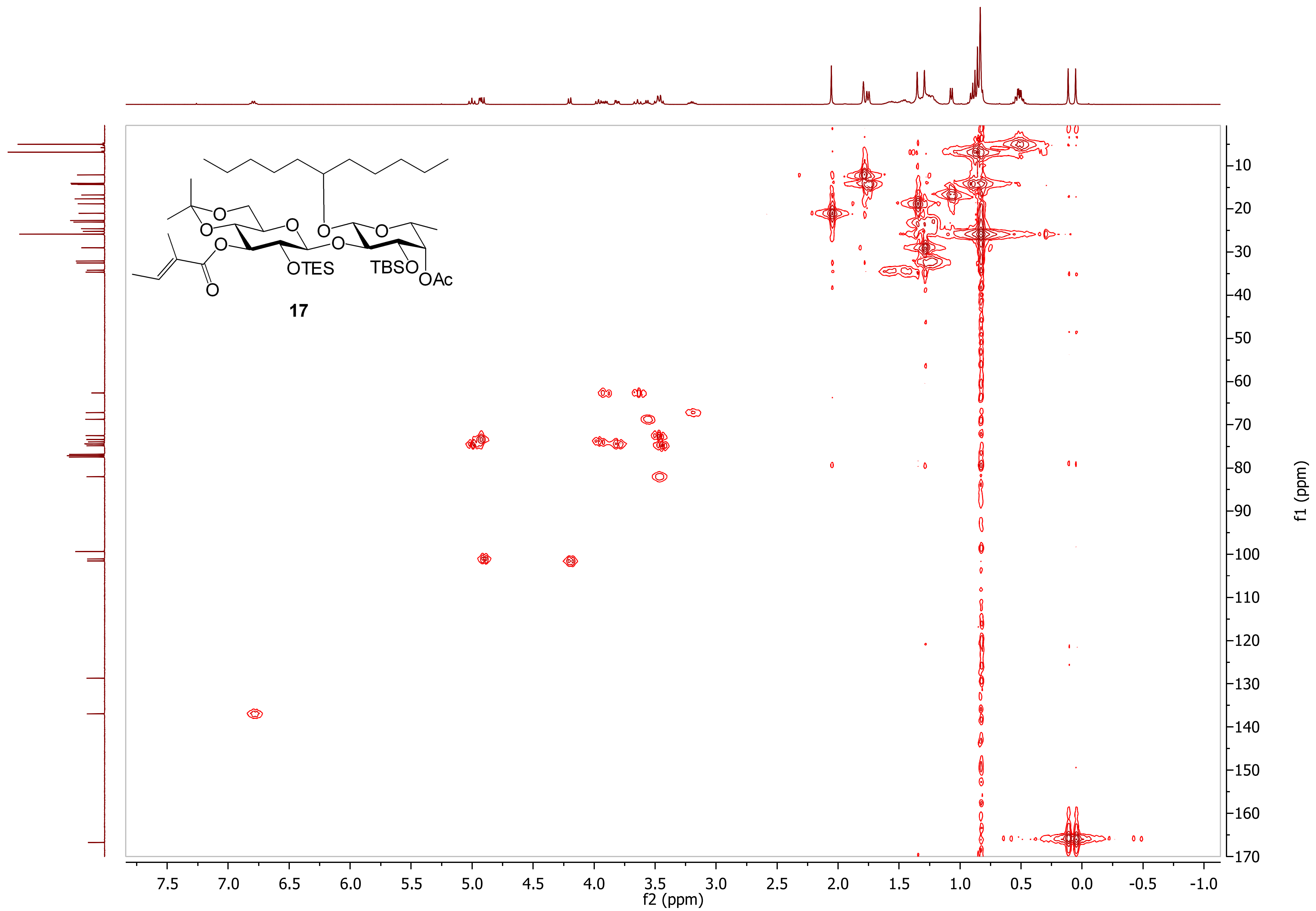

OTES
TBSO
OAc
17
f2 (ppm)
f1 (ppm)

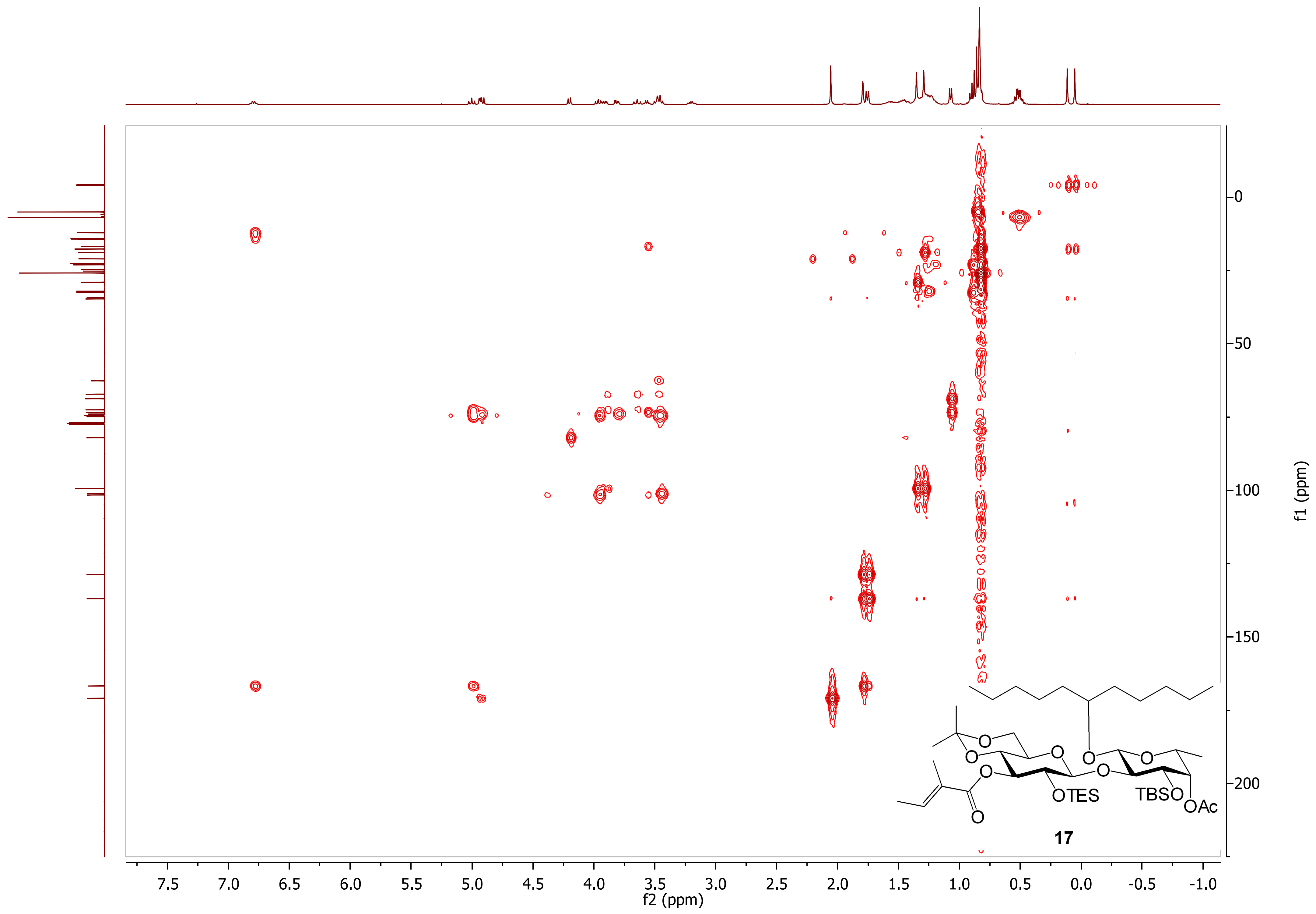

7.5
7.0
6.5
6.0
5.5
5.0
4.5
4.0
3.5
3.0
2.5
2.0
1.5
1.0
0.5
0.0
-0.5
-1.0
f2 (ppm)
0
50
100
150
200
f1 (ppm)
O
O
O
O
O
O
O
OTES
TBSO
OAc
O
17

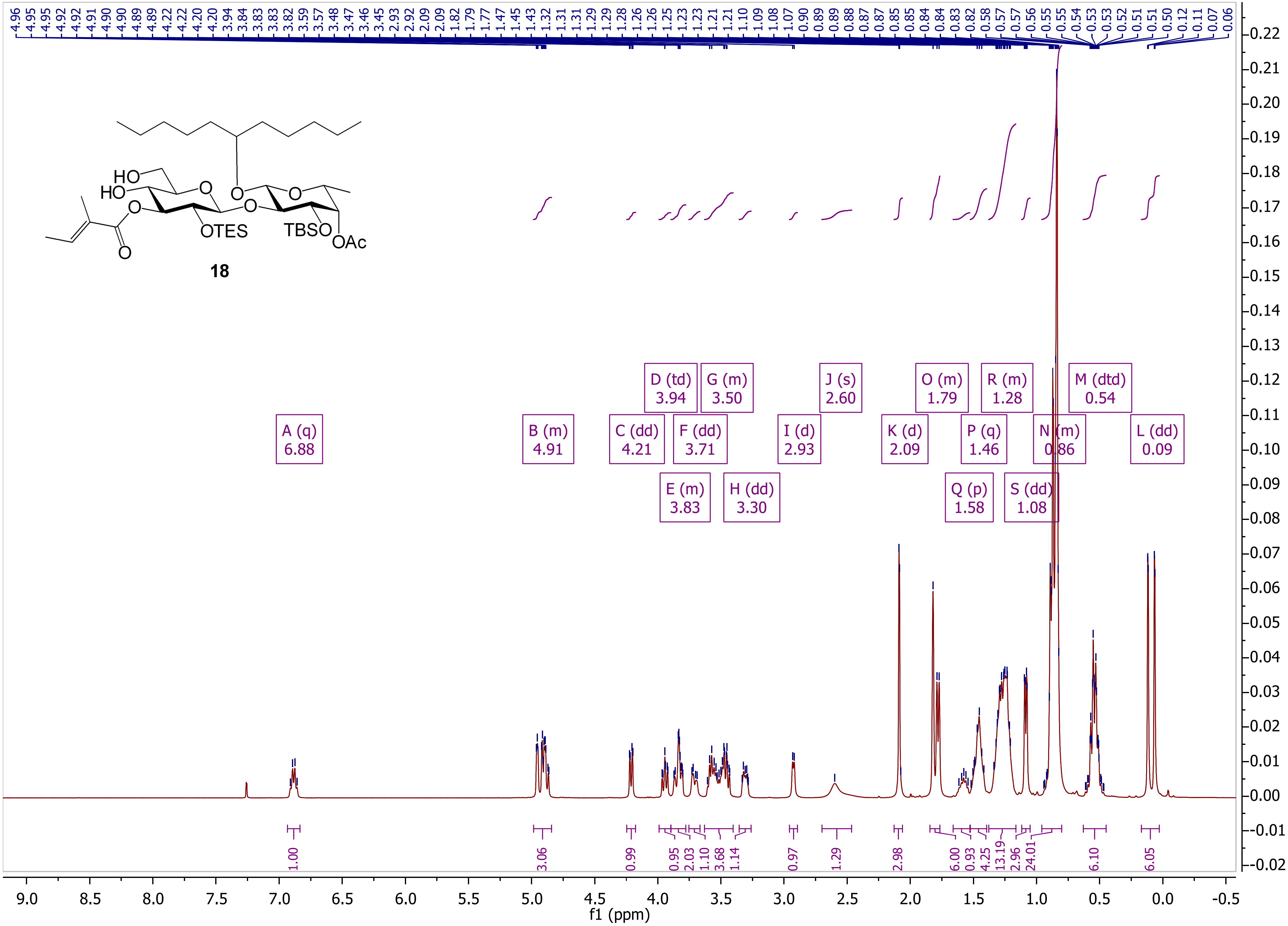

18
A (q) 6.88
B (m) 4.91
C (dd) 4.21
D (td) 3.94
E (m) 3.83
F (dd) 3.71
G (m) 3.50
H (dd) 3.30
I (d) 2.93
J (s) 2.60
K (d) 2.09
O (m) 1.79
P (q) 1.46
Q (p) 1.58
R (m) 1.28
S (dd) 1.08
N (m) 0.86
M (dtd) 0.54
L (dd) 0.09
1.00
3.06
0.99
0.95
2.03
1.10
3.68
1.14
0.97
1.29
2.98
6.00
0.93
4.25
13.19
2.96
24.01
6.10
6.05
f1 (ppm)

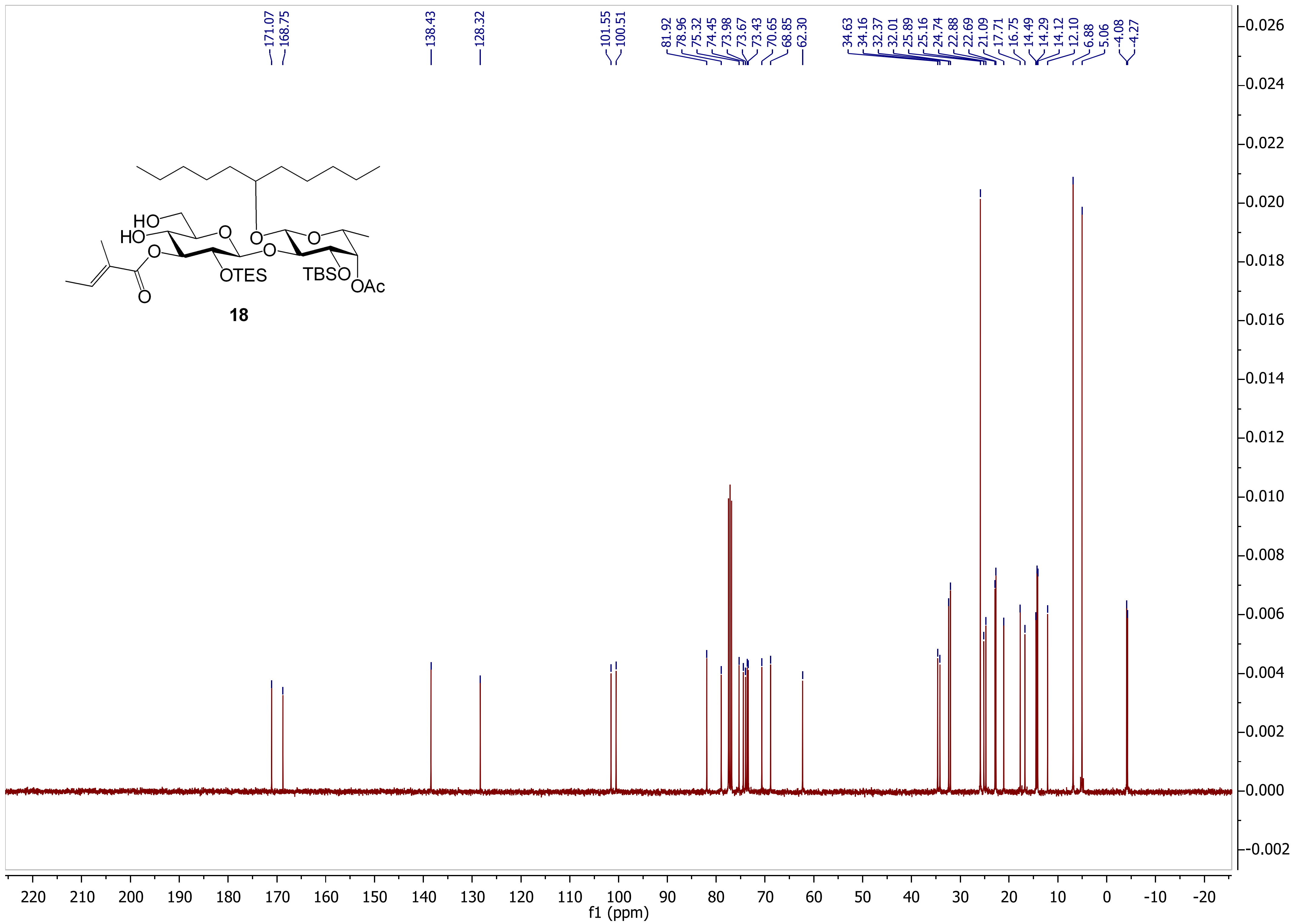

171.07
168.75
138.43
128.32
101.55
100.51
81.92
78.96
75.32
74.45
73.98
73.67
73.43
70.65
68.85
62.30
34.63
34.16
32.37
32.01
25.89
25.16
24.74
22.88
22.69
21.09
17.71
16.75
14.49
14.29
14.12
12.10
6.88
5.06
-4.08
-4.27
HO
HO
O
OTES
TBSO
OAc
18
f1 (ppm)

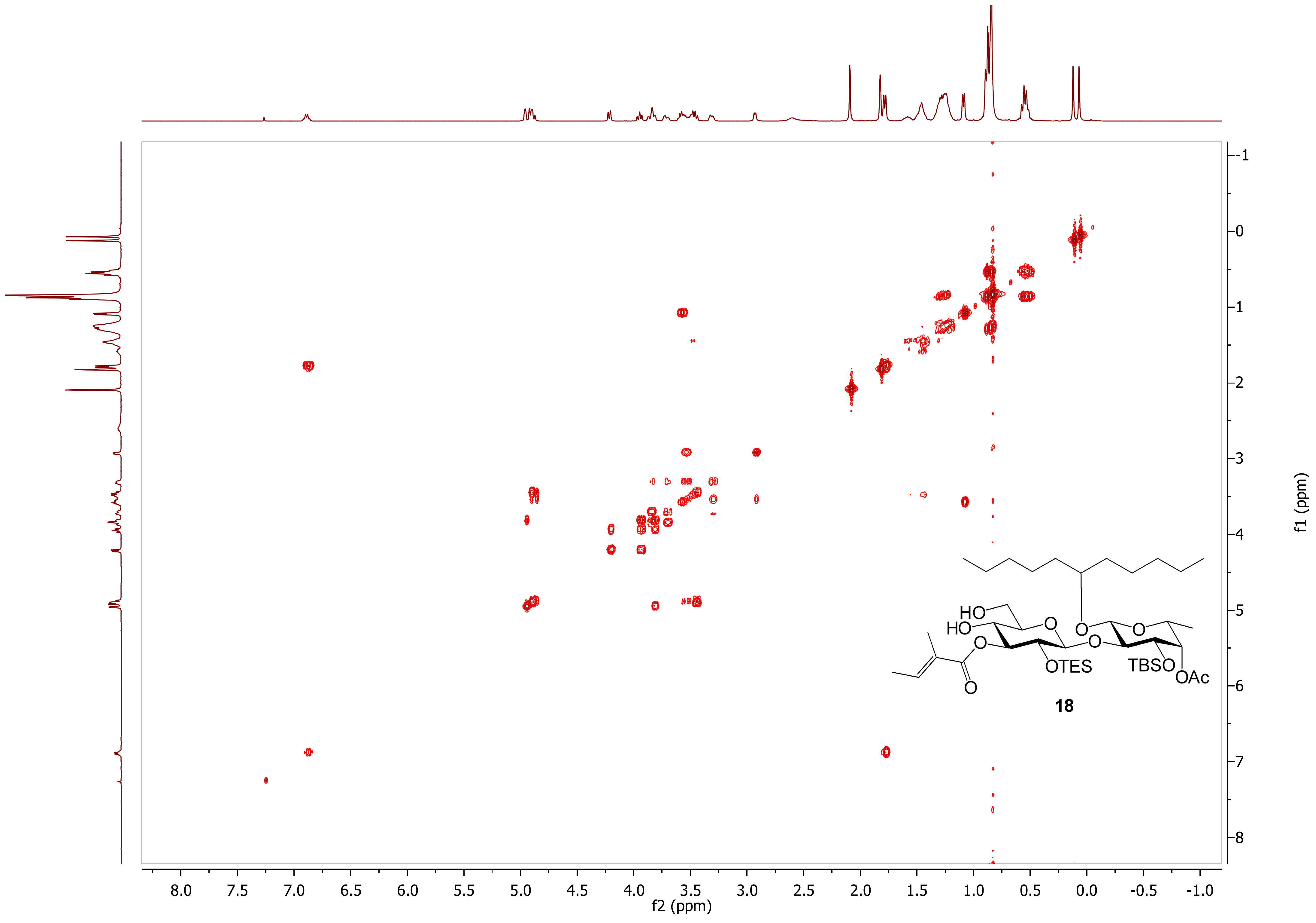

HO
HO
O
O
O
O
O
OTES
TBSO
OAc
O
18
f2 (ppm)
f1 (ppm)

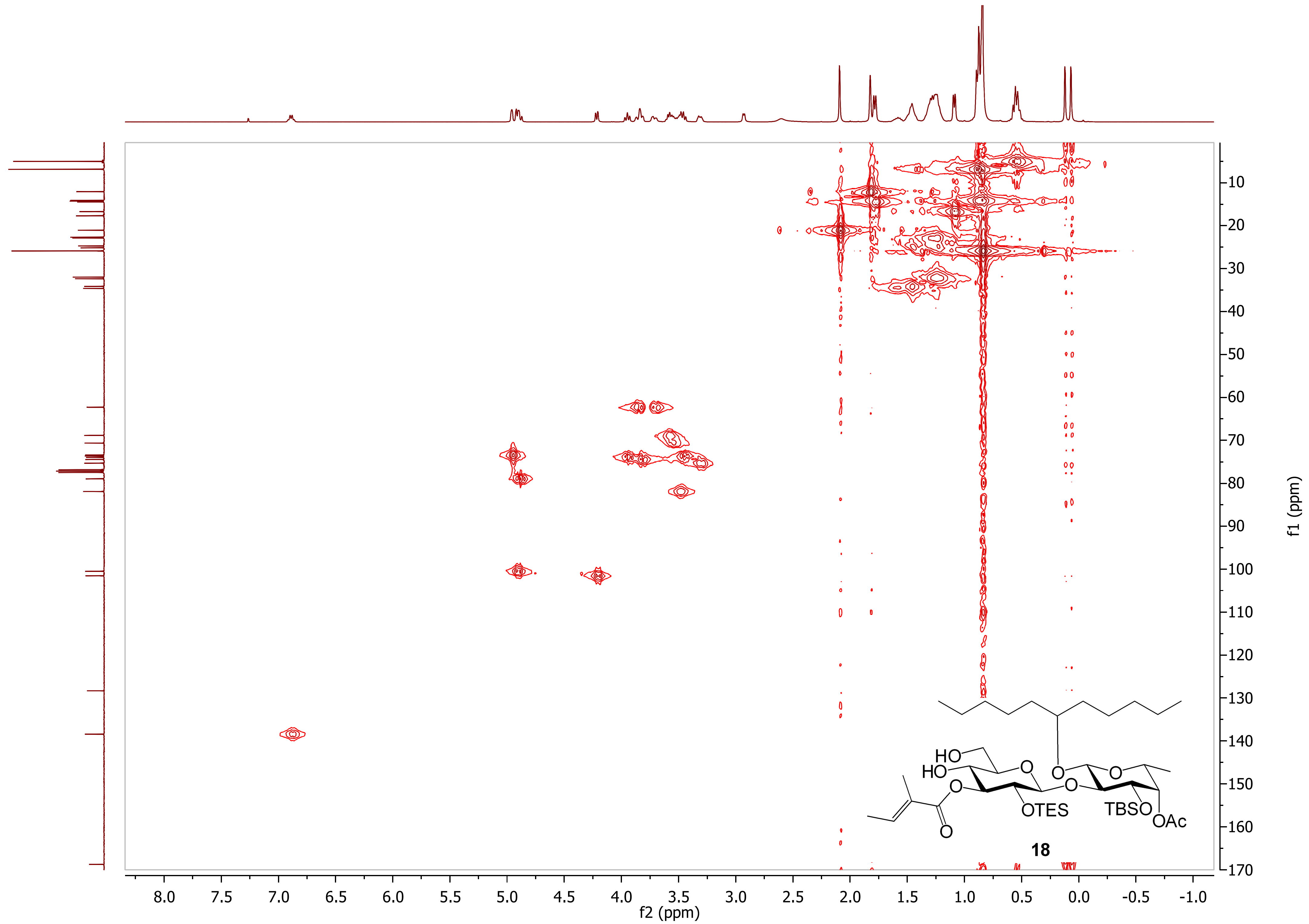
f1 (ppm)
f2 (ppm)
HO
HO
O
O
O
O
O
OTES
TBSO
OAc
O
18

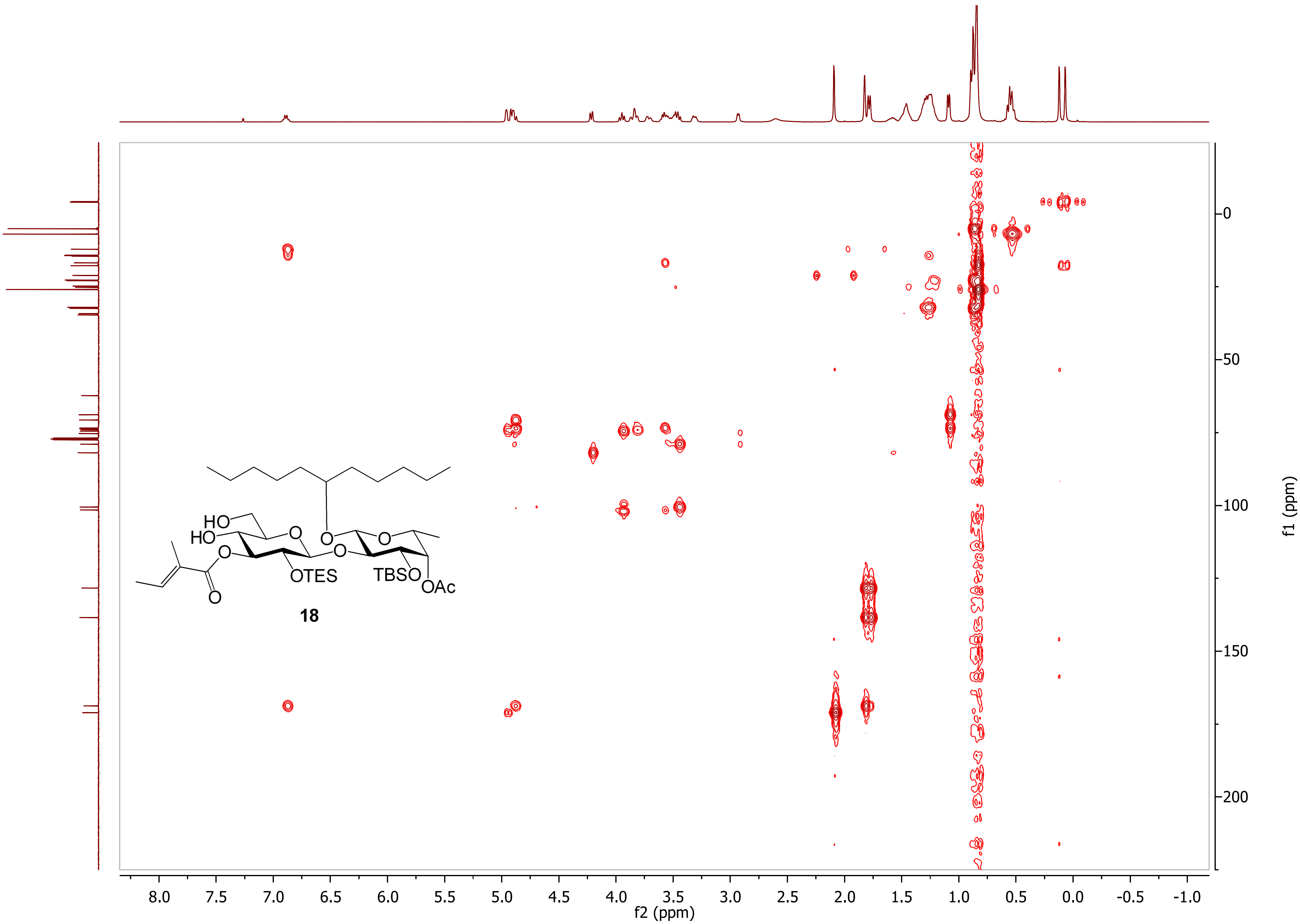
HO
HO
O
O
O
O
O
O
OTES
TBSO
OAc
18
8.0
7.5
7.0
6.5
6.0
5.5
5.0
4.5
4.0
3.5
3.0
2.5
2.0
1.5
1.0
0.5
0.0
-0.5
-1.0
f2 (ppm)
0
50
100
150
200
f1 (ppm)

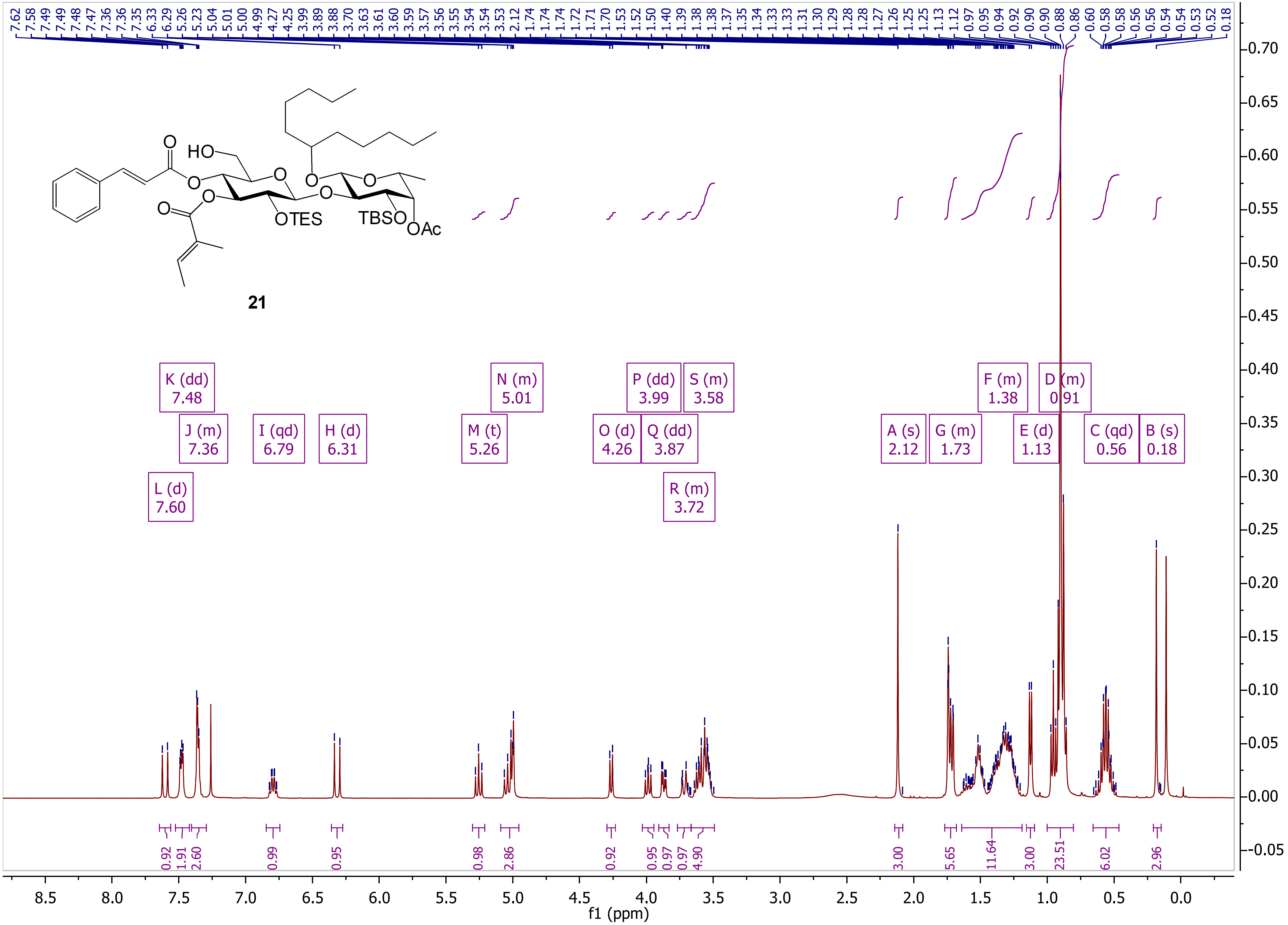
7.62 7.58 7.49 7.49 7.48 7.47 7.36 7.36 7.35 6.33 6.29 5.26 5.23 5.04 5.01 5.00 4.99 4.27 4.25 3.99 3.89 3.88 3.70 3.63 3.61 3.60 3.59 3.57 3.56 3.55 3.54 3.54 3.53 2.12 1.74 1.74 1.74 1.72 1.71 1.70 1.53 1.52 1.50 1.40 1.39 1.38 1.38 1.37 1.35 1.34 1.33 1.33 1.31 1.30 1.29 1.28 1.28 1.27 1.26 1.25 1.25 1.13 1.12 0.97 0.95 0.94 0.92 0.90 0.90 0.88 0.86 0.60 0.58 0.58 0.56 0.56 0.54 0.54 0.53 0.52 0.18
HO
O
OTES
TBSO
OAc
21
K (dd) 7.48
J (m) 7.36
L (d) 7.60
I (qd) 6.79
H (d) 6.31
N (m) 5.01
M (t) 5.26
O (d) 4.26
P (dd) 3.99
Q (dd) 3.87
S (m) 3.58
R (m) 3.72
A (s) 2.12
G (m) 1.73
F (m) 1.38
E (d) 1.13
D (m) 0.91
C (qd) 0.56
B (s) 0.18
0.92 1.91 2.60 0.99 0.95 0.98 2.86 0.92 0.95 0.97 0.97 4.90 3.00 5.65 11.64 3.00 23.51 6.02 2.96
f1 (ppm)

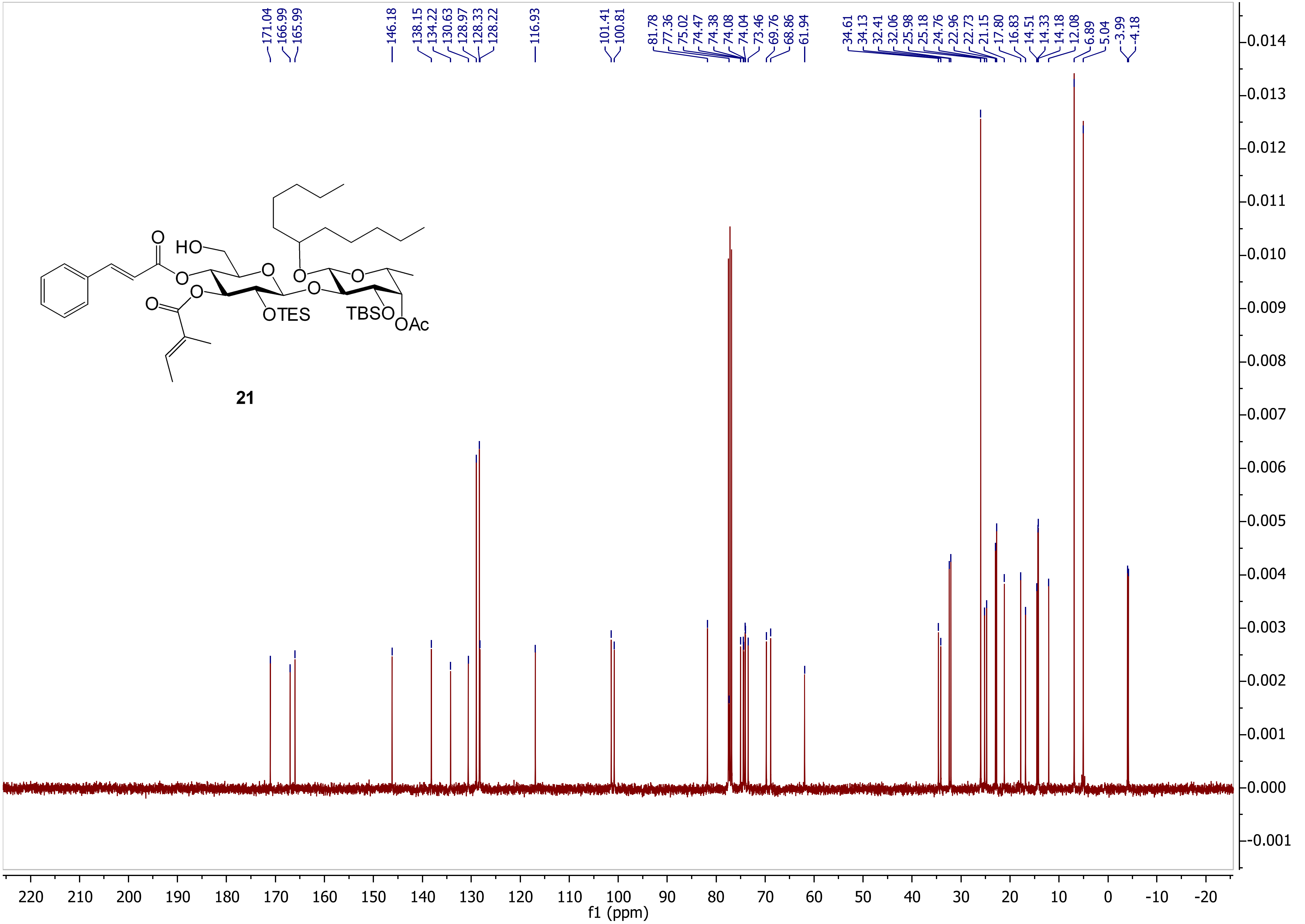
171.04
166.99
165.99
146.18
138.15
134.22
130.63
128.97
128.33
128.22
116.93
101.41
100.81
81.78
77.36
75.02
74.47
74.38
74.08
74.04
73.46
69.76
68.86
61.94
34.61
34.13
32.41
32.06
25.98
25.18
24.76
22.96
22.73
21.15
17.80
16.83
14.51
14.33
14.18
12.08
6.89
5.04
-3.99
-4.18
HO
OTES
TBSO
OAc
21
f1 (ppm)

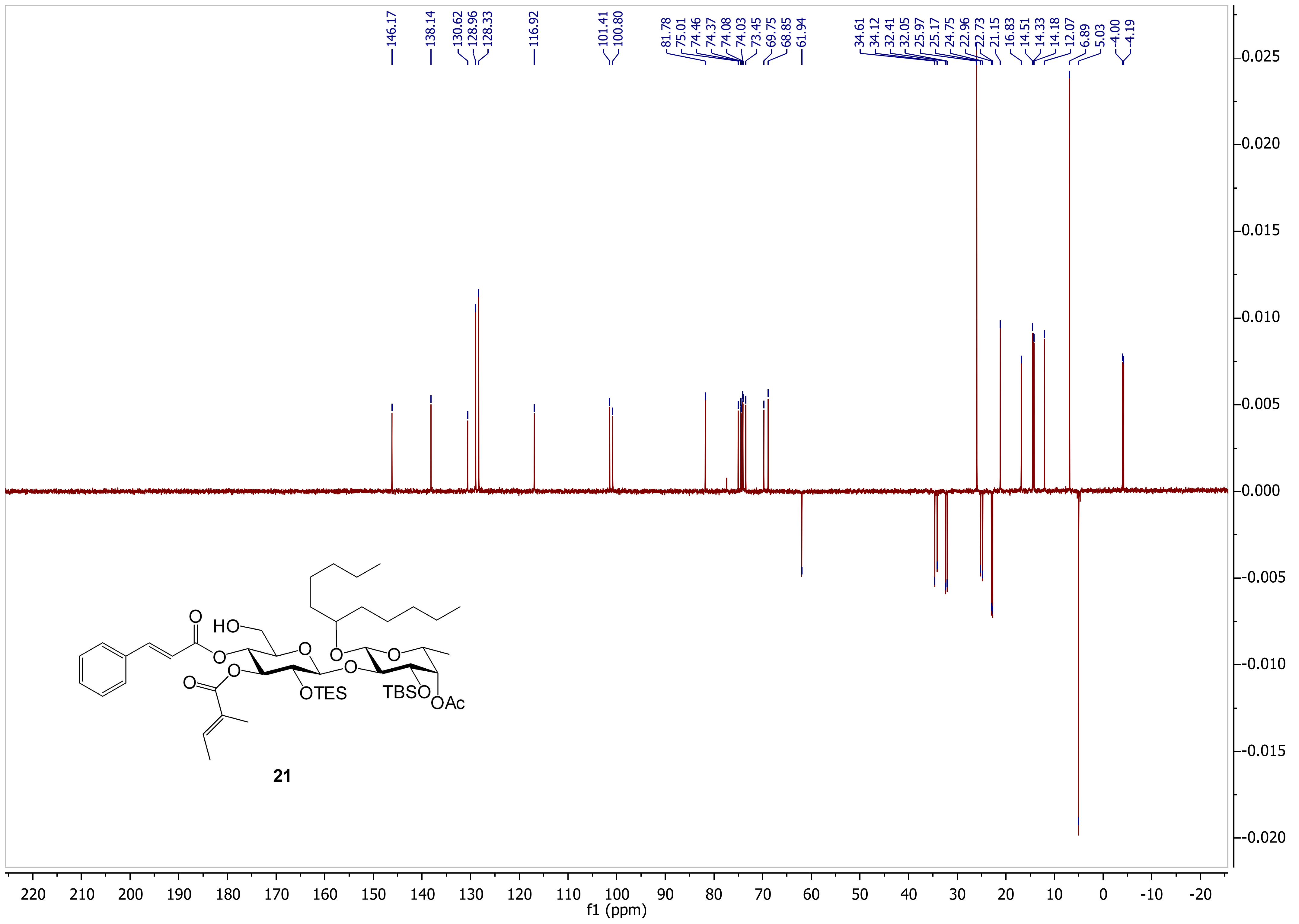
146.17
138.14
130.62
128.96
128.33
116.92
101.41
100.80
81.78
75.01
74.46
74.37
74.08
74.03
73.45
69.75
68.85
61.94
34.61
34.12
32.41
32.05
25.97
25.17
24.75
22.96
22.73
21.15
16.83
14.51
14.33
14.18
12.07
6.89
5.03
-4.00
-4.19
HO
OTES
TBSO
OAc
21
f1 (ppm)

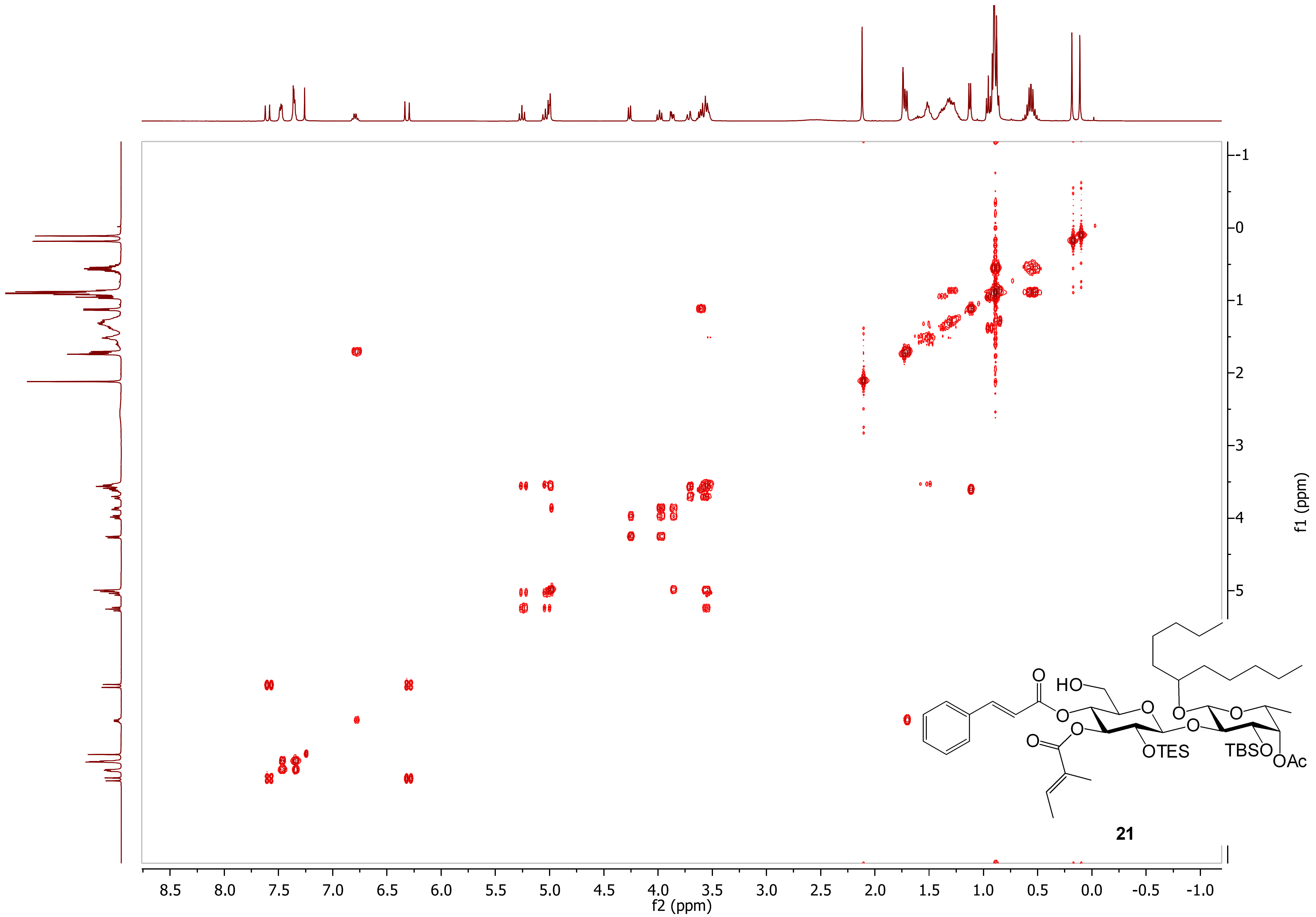

f2 (ppm)
f1 (ppm)
HO
O
OTES
TBSO
OAc
21

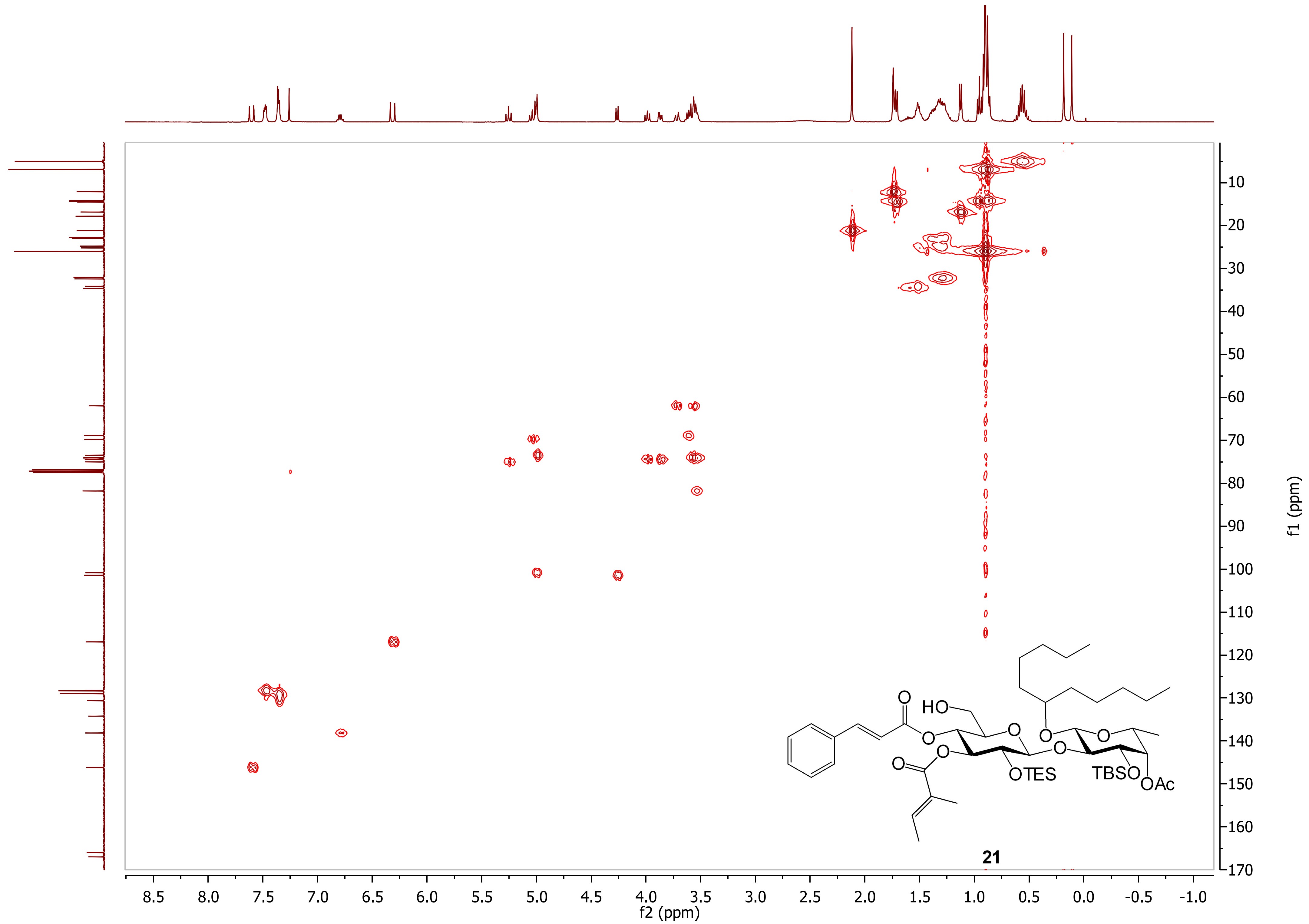

f1 (ppm)
f2 (ppm)
HO
O
OTES
TBSO
OAc
21

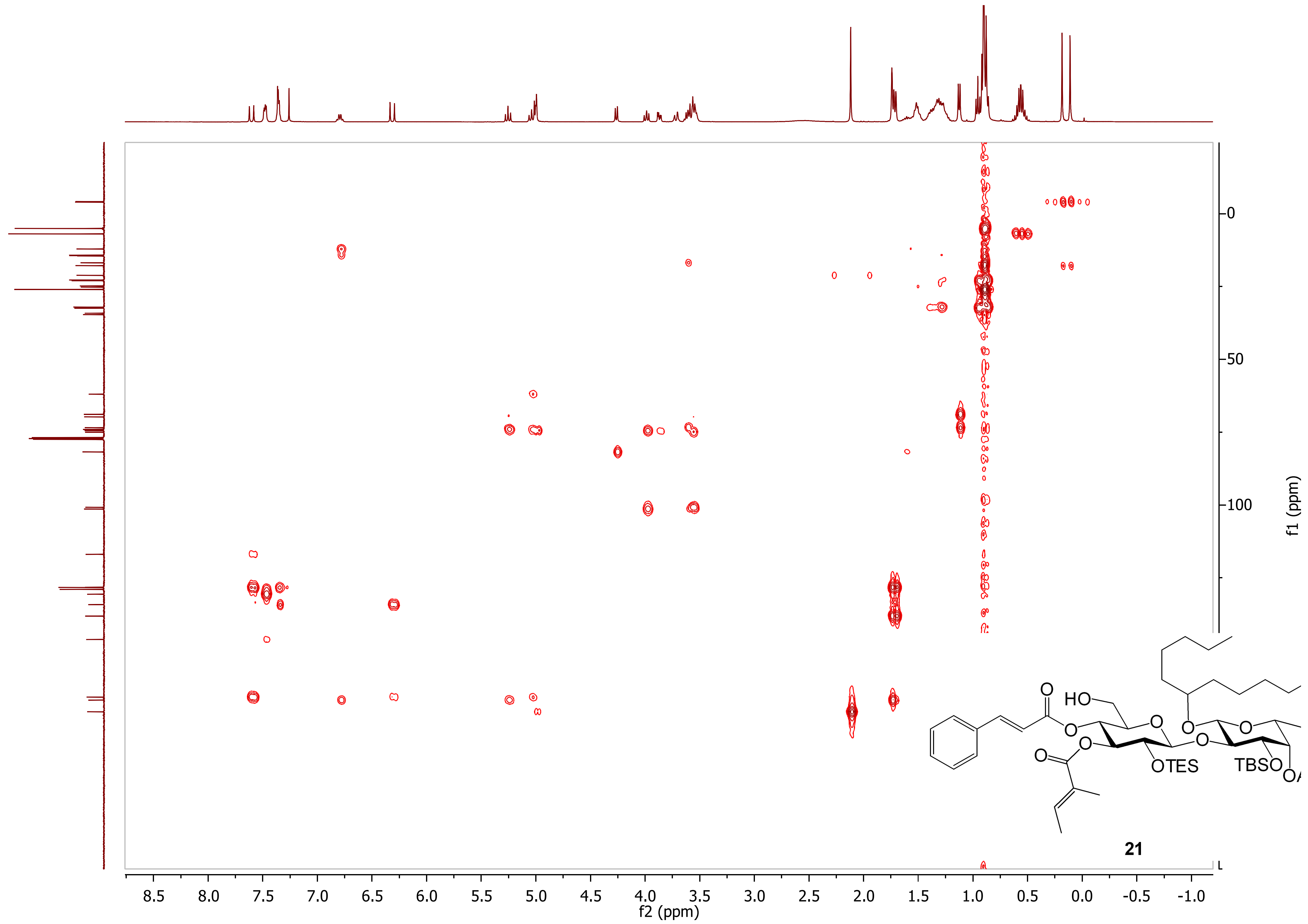
8.5
8.0
7.5
7.0
6.5
6.0
5.5
5.0
4.5
4.0
3.5
3.0
2.5
2.0
1.5
1.0
0.5
0.0
-0.5
-1.0
f2 (ppm)
0
50
100
f1 (ppm)
HO
O
O
O
O
O
O
O
OTES
TBSO
21

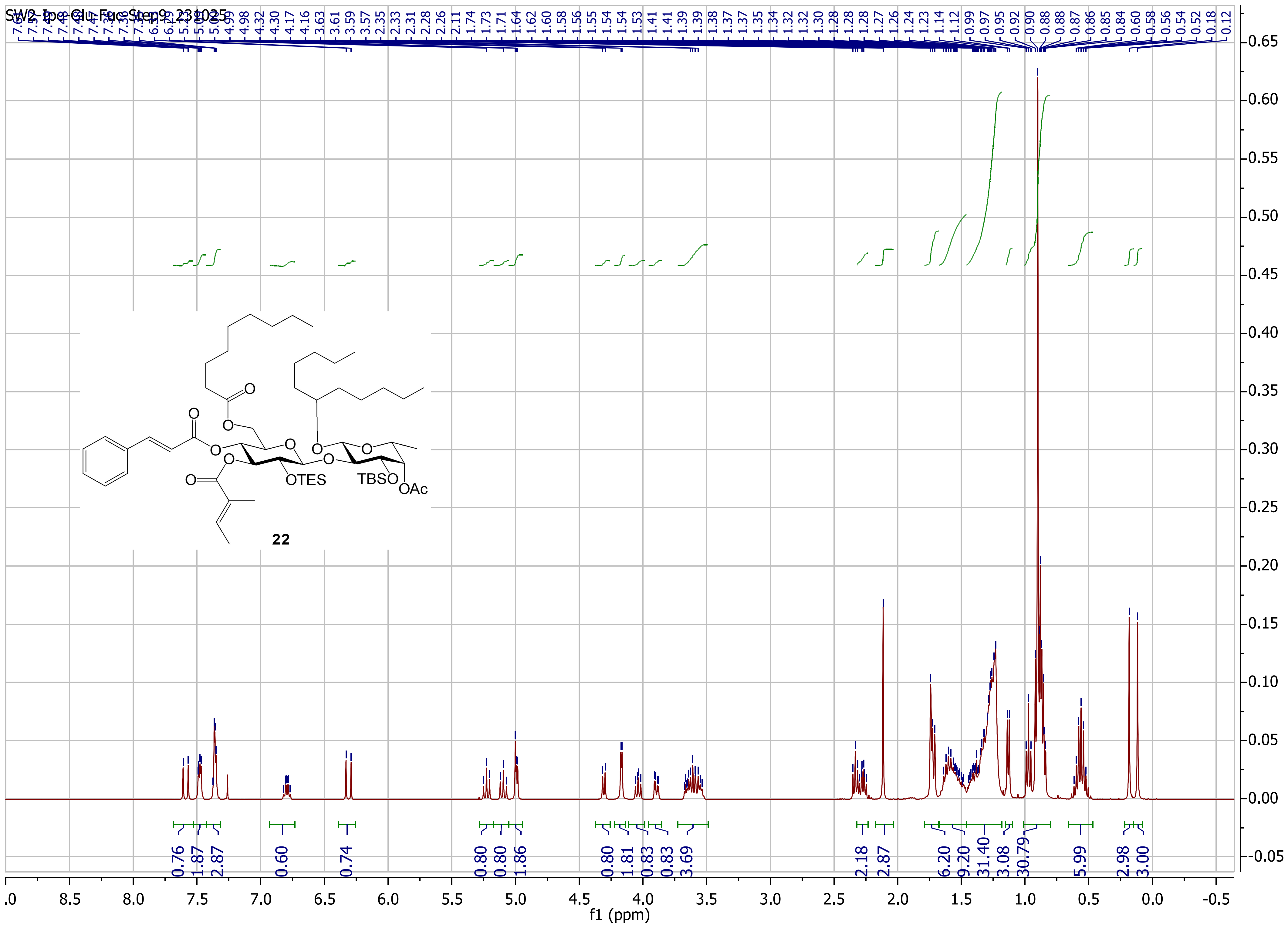

SW2-Ipe-Glu-Fuc-Step9-231025
4.98
4.32
4.30
4.17
4.16
3.63
3.61
3.59
3.57
2.35
2.33
2.31
2.28
2.26
2.11
1.74
1.73
1.71
1.64
1.62
1.60
1.58
1.56
1.55
1.54
1.54
1.53
1.41
1.41
1.39
1.39
1.38
1.37
1.37
1.35
1.34
1.32
1.32
1.30
1.28
1.28
1.28
1.27
1.26
1.24
1.23
1.14
1.12
0.99
0.97
0.95
0.92
0.90
0.88
0.88
0.87
0.86
0.85
0.84
0.60
0.58
0.56
0.54
0.52
0.18
0.12
O
O
O
O
O
O
O
O
OTES
TBSO
OAc
22
0.76
1.87
2.87
0.60
0.74
0.80
0.80
1.86
0.80
1.81
0.83
0.83
3.69
2.18
2.87
6.20
9.20
31.40
3.08
30.79
5.99
2.98
3.00
f1 (ppm)

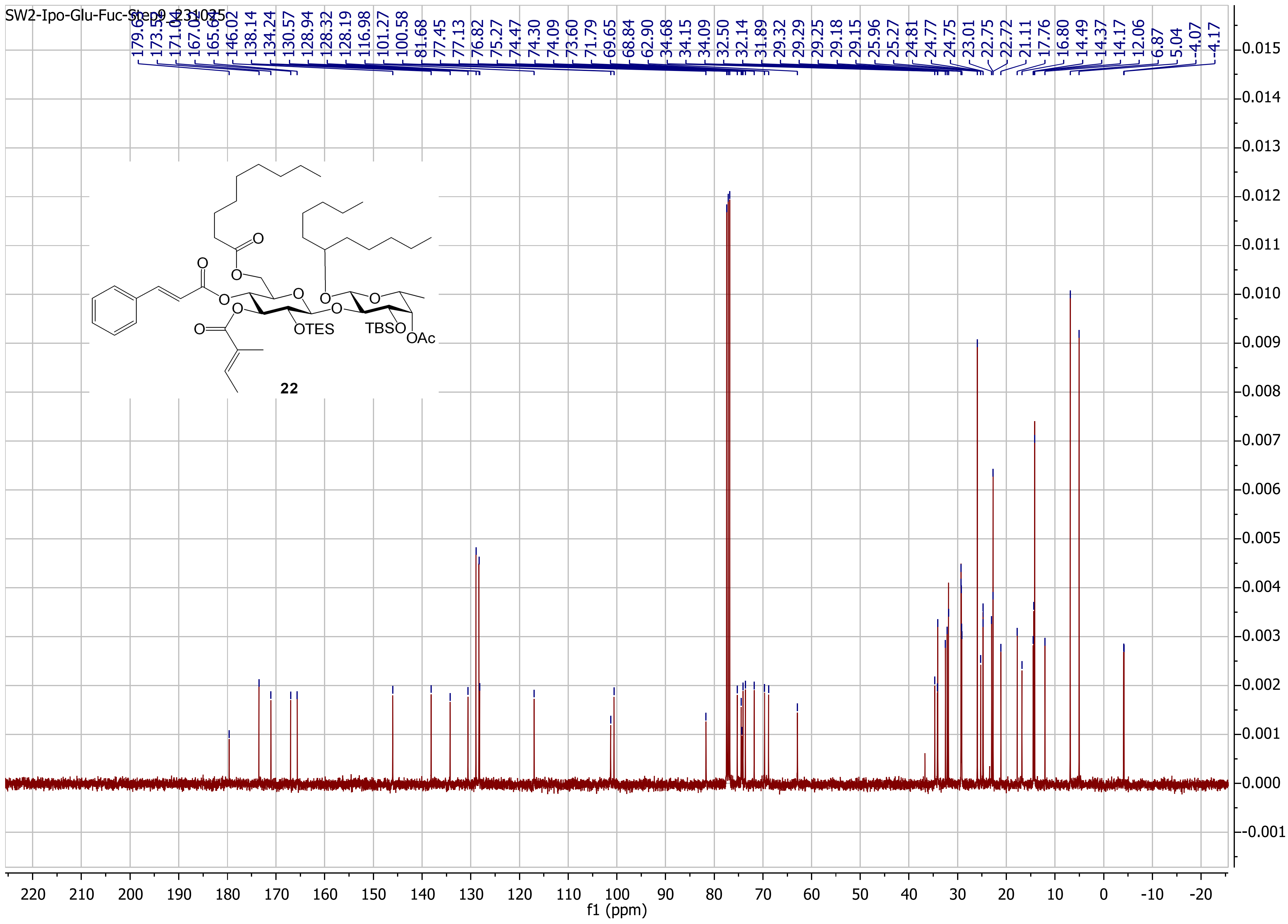

SW2-Ipo-Glu-Fuc-Step9_231025
179.6
173.5
171.0
167.0
165.6
146.02
138.14
134.24
130.57
128.94
128.32
128.19
116.98
101.27
100.58
81.68
77.45
77.13
76.82
75.27
74.47
74.30
74.09
73.60
71.79
69.65
68.84
62.90
34.68
34.15
34.09
32.50
32.14
31.89
29.32
29.29
29.25
29.18
29.15
25.96
25.27
24.81
24.77
24.75
23.01
22.75
22.72
21.11
17.76
16.80
14.49
14.37
14.17
12.06
6.87
5.04
-4.07
-4.17
O
OTES
TBSO
OAc
22
f1 (ppm)

SW2-Ipo-Glu-Fuc-Step9_231025

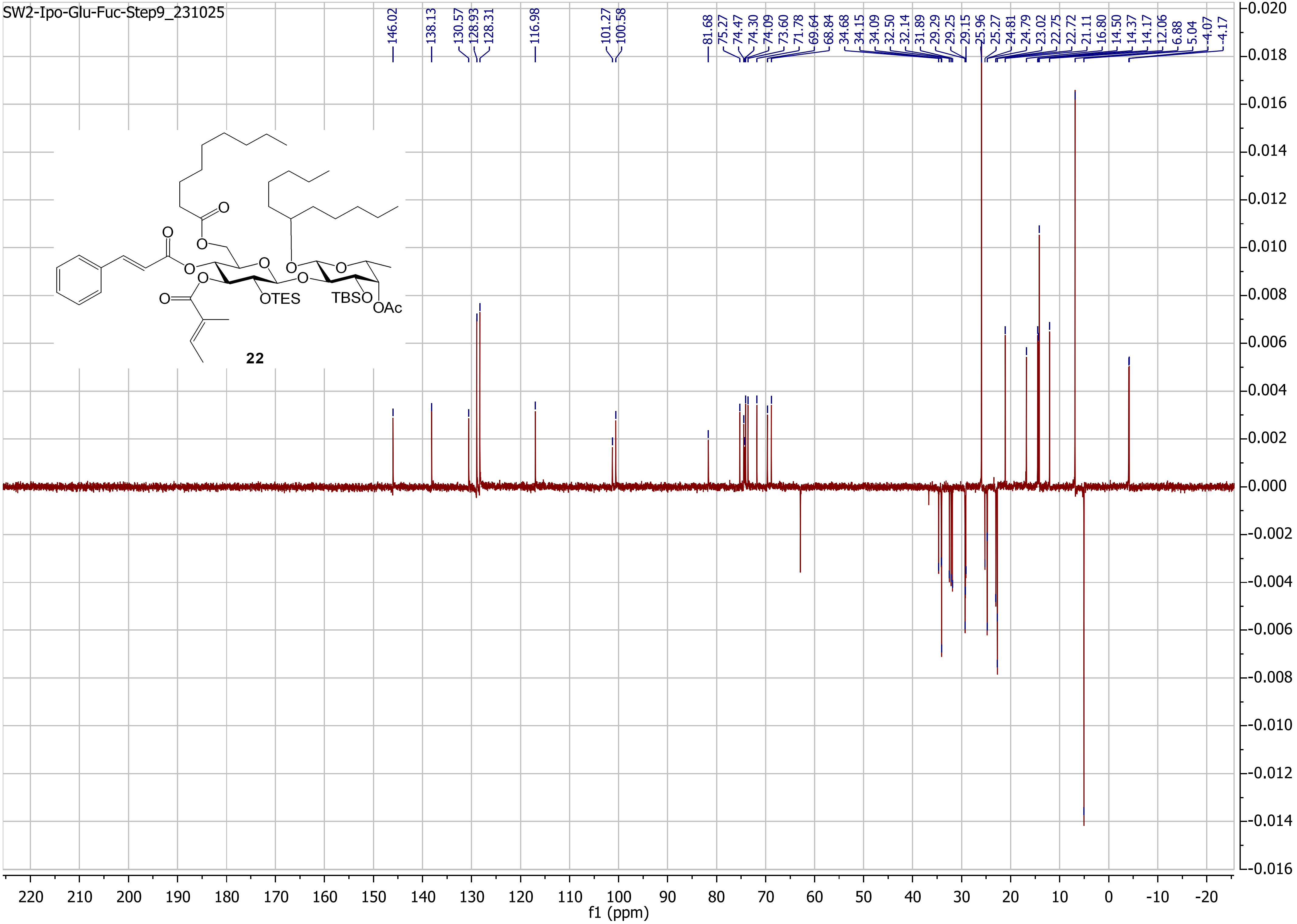

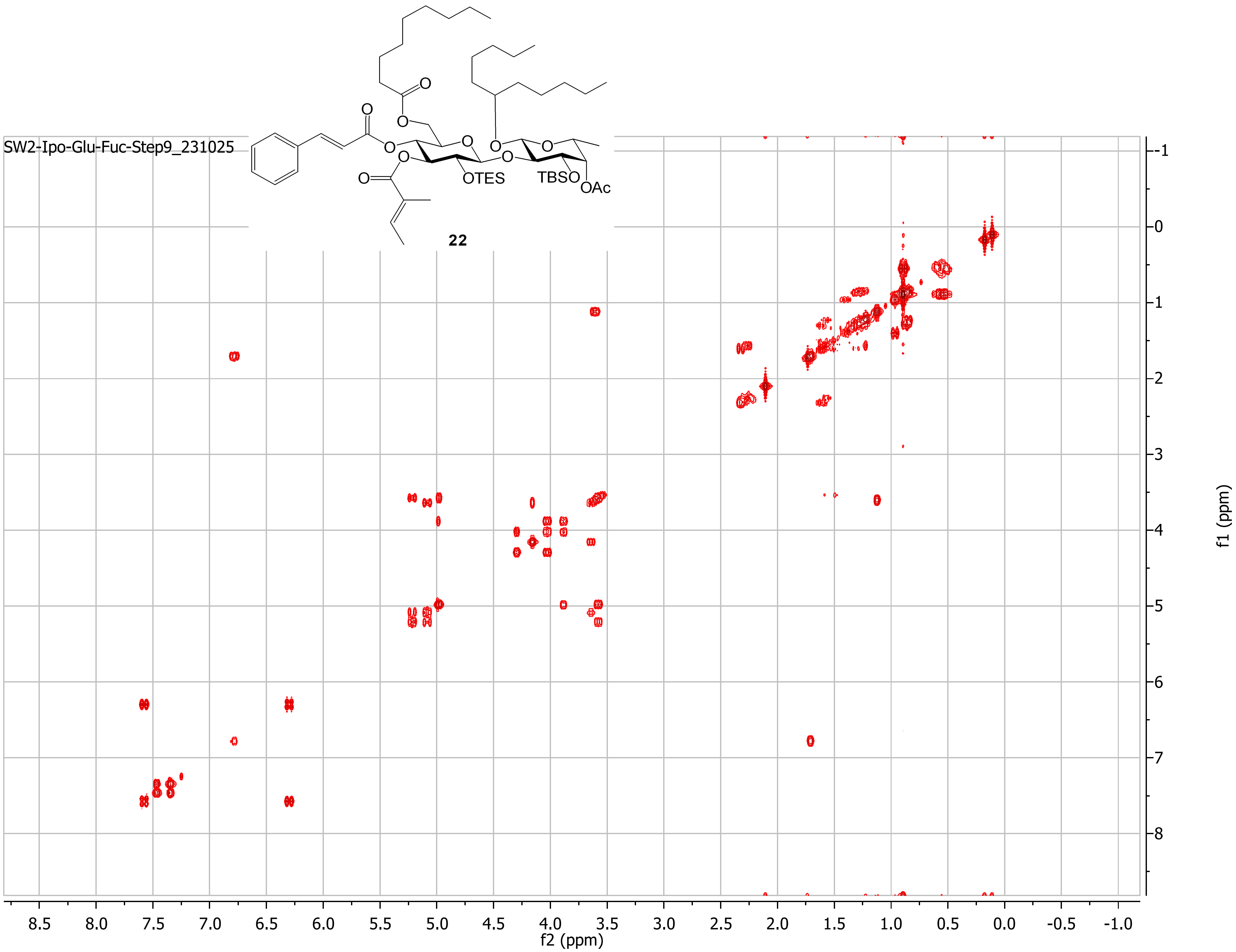
SW2-Ipo-Glu-Fuc-Step9_231025
O
O
O
O
O
O
O
O
OTES
TBSO
OAc
22
f2 (ppm)
f1 (ppm)
8.5
8.0
7.5
7.0
6.5
6.0
5.5
5.0
4.5
4.0
3.5
3.0
2.5
2.0
1.5
1.0
0.5
0.0
-0.5
-1.0
-1
0
1
2
3
4
5
6
7
8

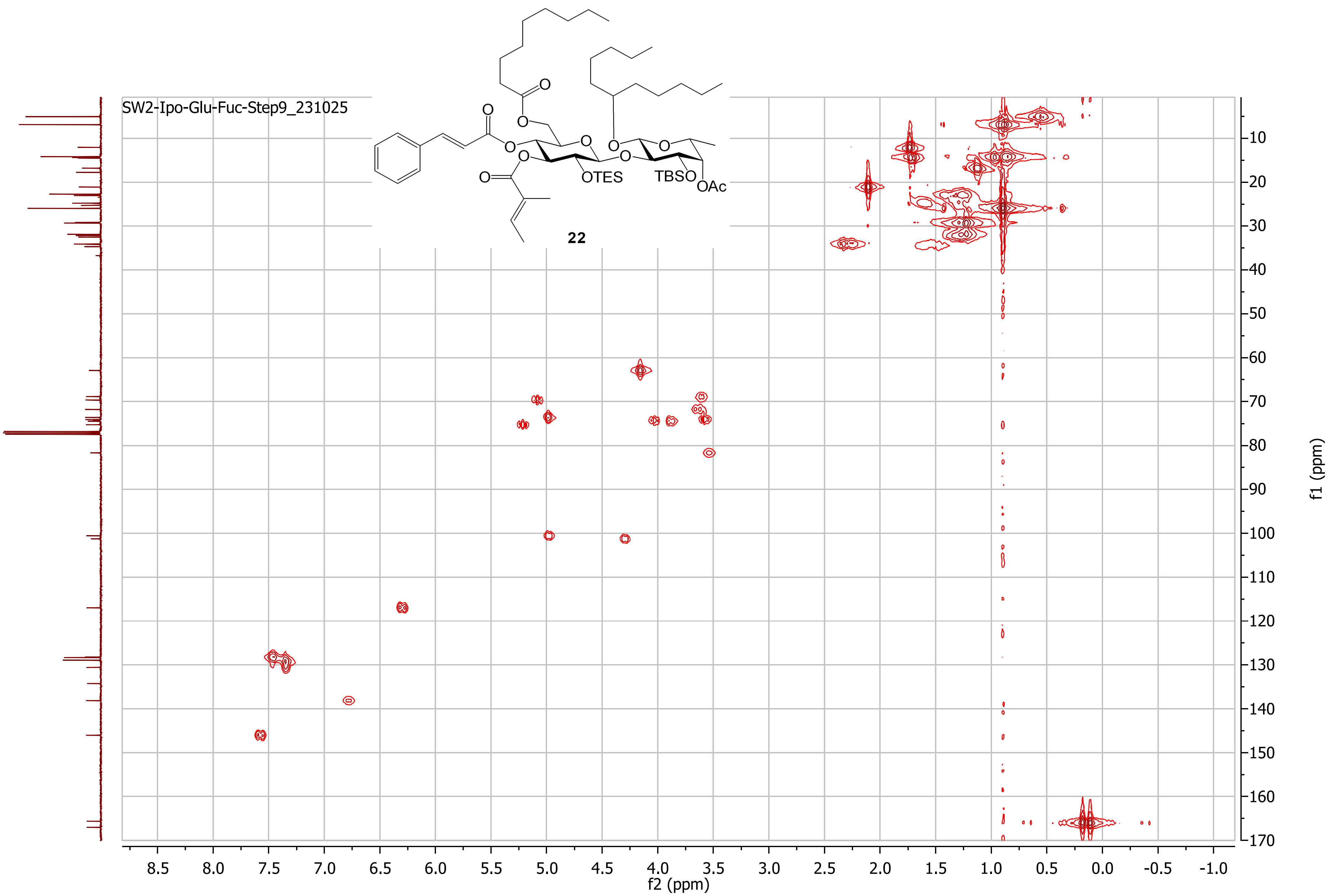
SW2-Ipo-Glu-Fuc-Step9_231025
OTES
TBSO
OAc
22
f2 (ppm)
f1 (ppm)

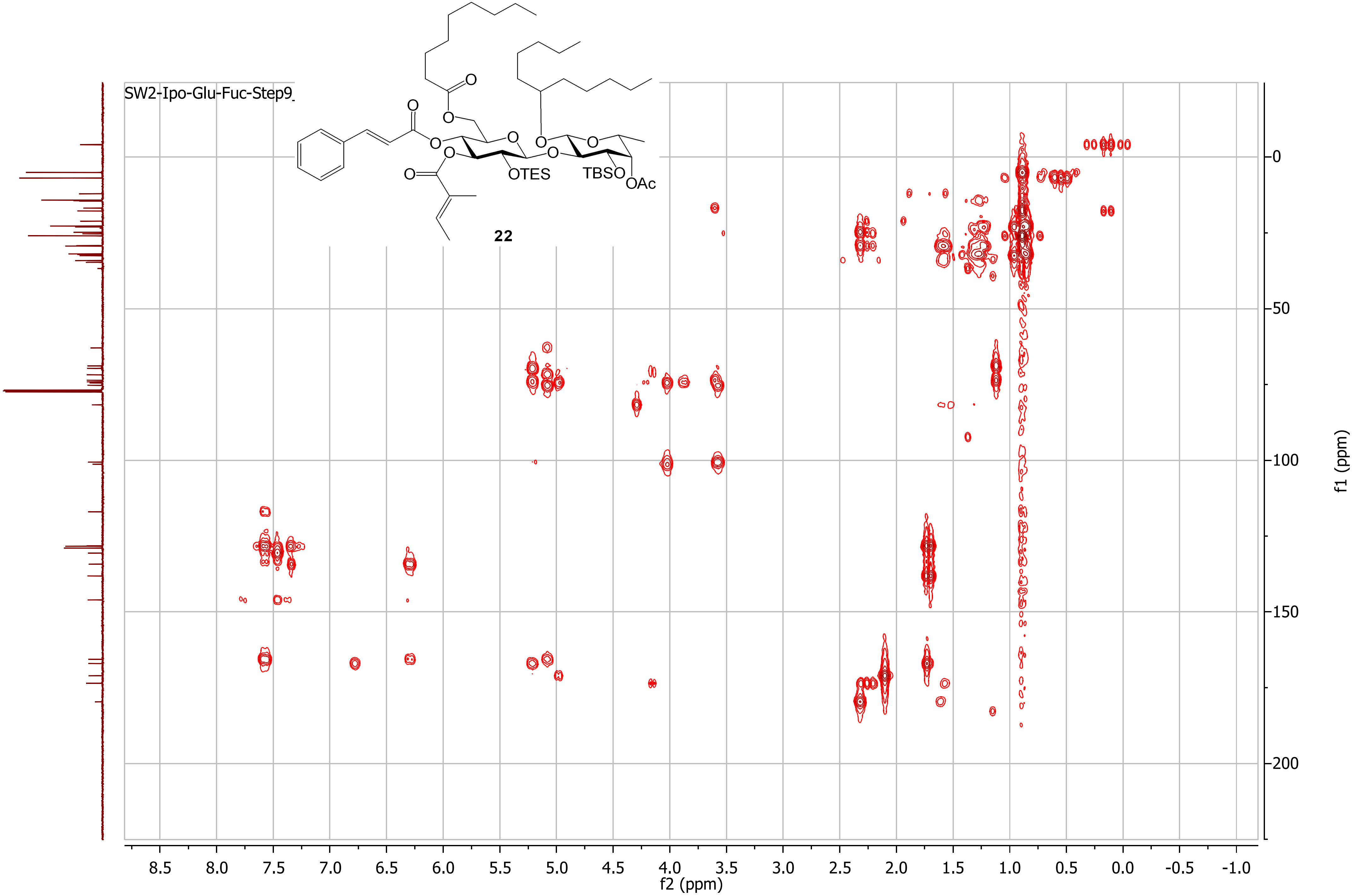
SW2-Ipo-Glu-Fuc-Step9
O
O
O
O
O
O
O
O
O
OTES
TBSO
OAc
22
f2 (ppm)
f1 (ppm)

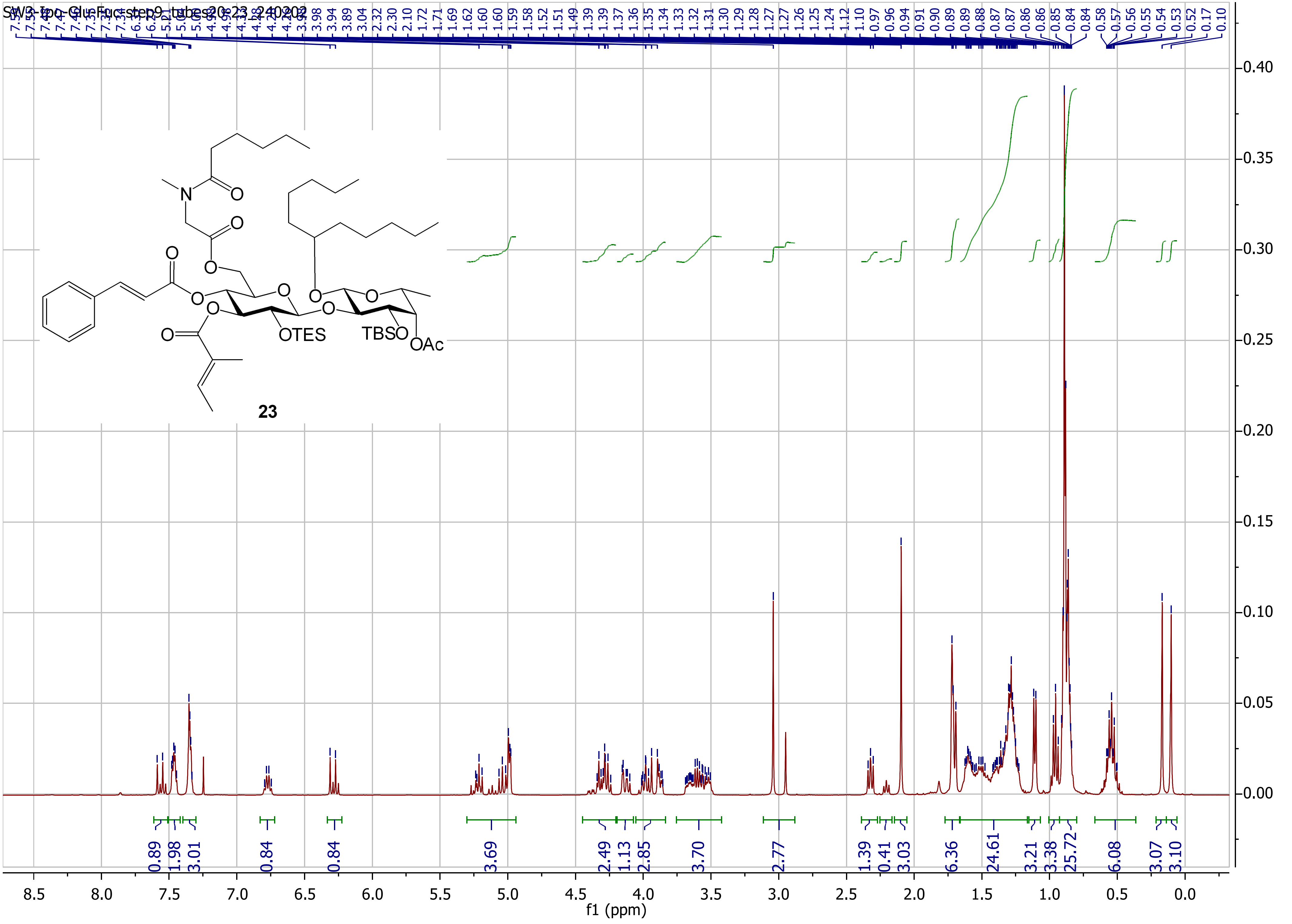
SW3-tpo-Glu-Fuc-step9_tubes20-23_240202
OTES
TBSO
OAc
23
0.89 1.98 3.01 0.84 0.84 3.69 2.49 1.13 2.85 3.70 2.77 1.39 0.41 3.03 6.36 24.61 3.21 3.38 25.72 6.08 3.07 3.10
f1 (ppm)

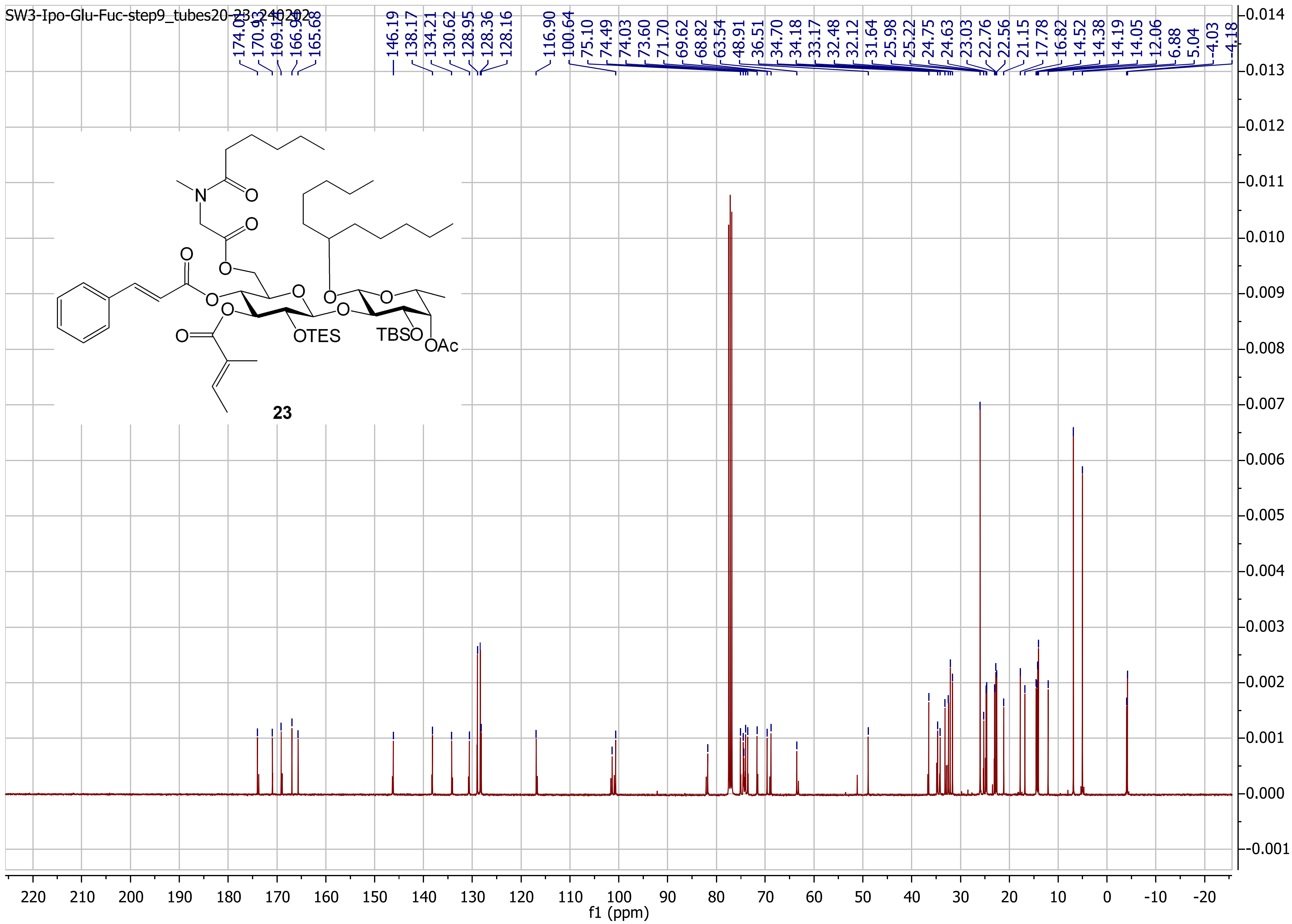

SW3-Ipo-Glu-Fuc-step9_tubes20-23_240202
174.01
170.93
169.14
166.94
165.68
146.19
138.17
134.21
130.62
128.95
128.36
128.16
116.90
100.64
75.10
74.49
74.03
73.60
71.70
69.62
68.82
63.54
48.91
36.51
34.70
34.18
33.17
32.48
32.12
31.64
25.98
25.22
24.75
24.63
23.03
22.76
22.56
21.15
17.78
16.82
14.52
14.38
14.19
14.05
12.06
6.88
5.04
-4.03
-4.18
O
N
OTES
TBSO
OAc
23
f1 (ppm)

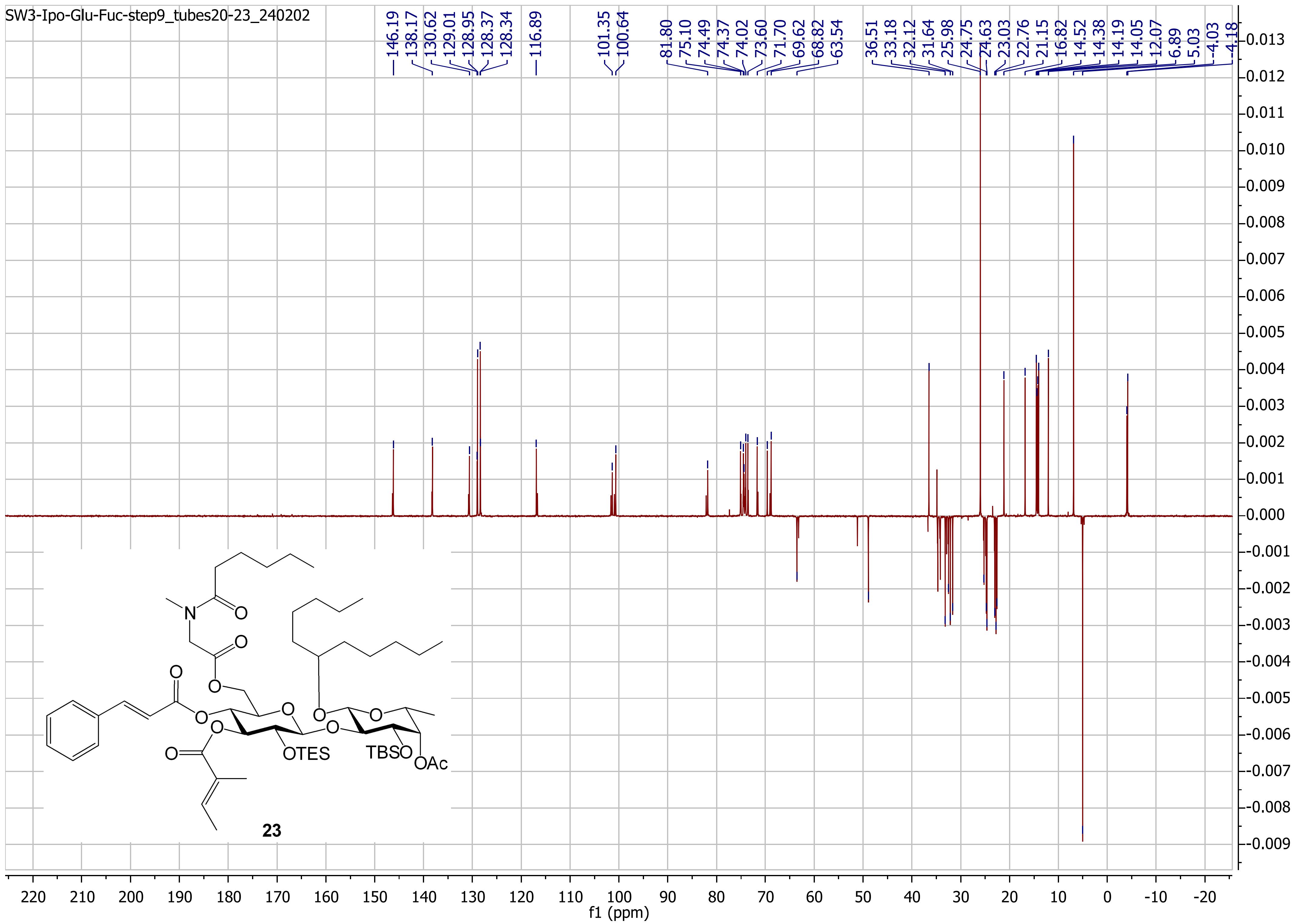
SW3-Ipo-Glu-Fuc-step9_tubes20-23_240202
146.19
138.17
130.62
129.01
128.95
128.37
128.34
116.89
101.35
100.64
81.80
75.10
74.49
74.37
74.02
73.60
71.70
69.62
68.82
63.54
36.51
33.18
32.12
31.64
25.98
24.75
24.63
23.03
22.76
21.15
16.82
14.52
14.38
14.19
14.05
12.07
6.89
5.03
-4.03
-4.18
f1 (ppm)
N
O
OTES
TBSO
OAc
23

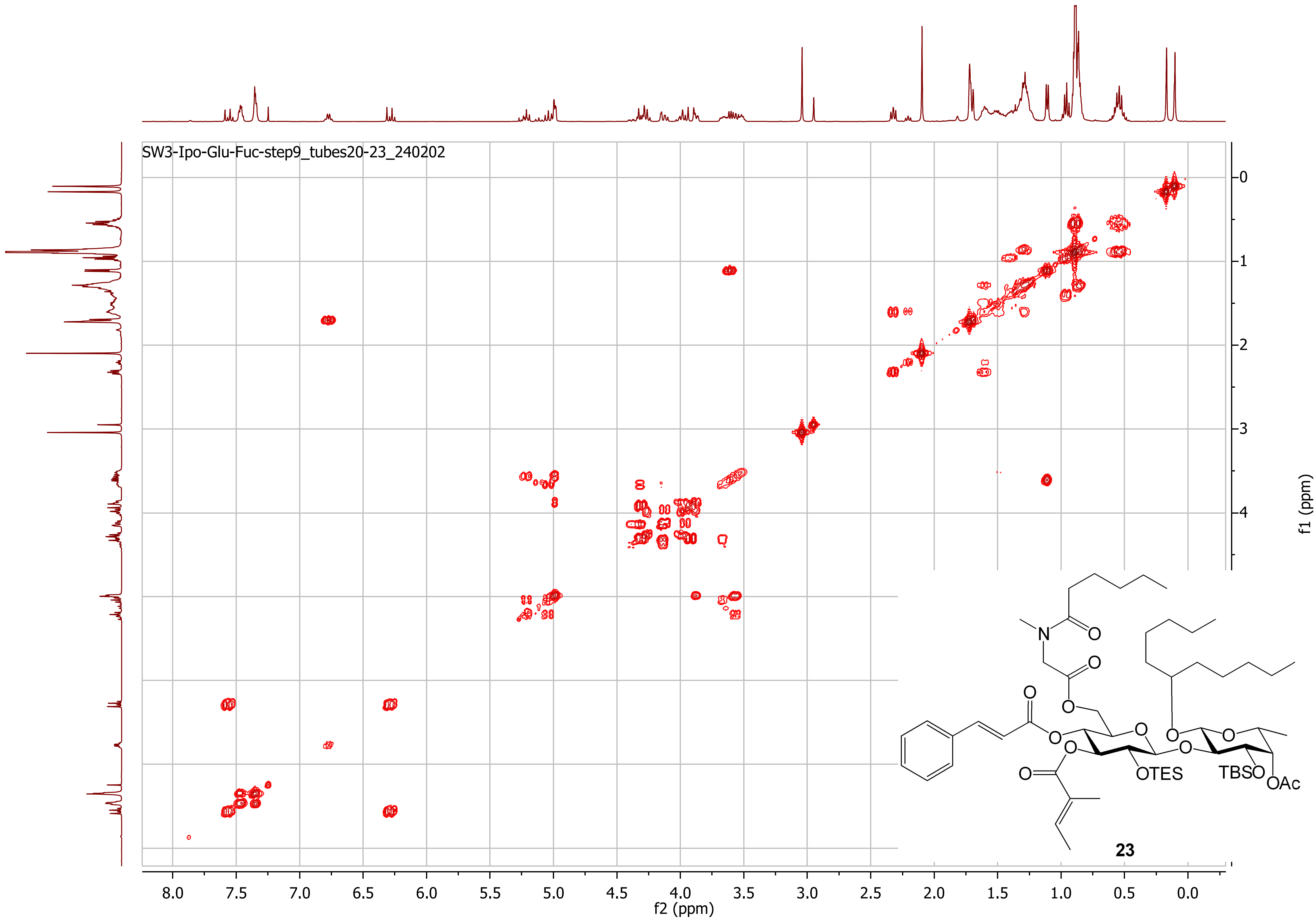
SW3-Ipo-Glu-Fuc-step9_tubes20-23_240202
f2 (ppm)
f1 (ppm)
OTES
TBSO
OAc

23

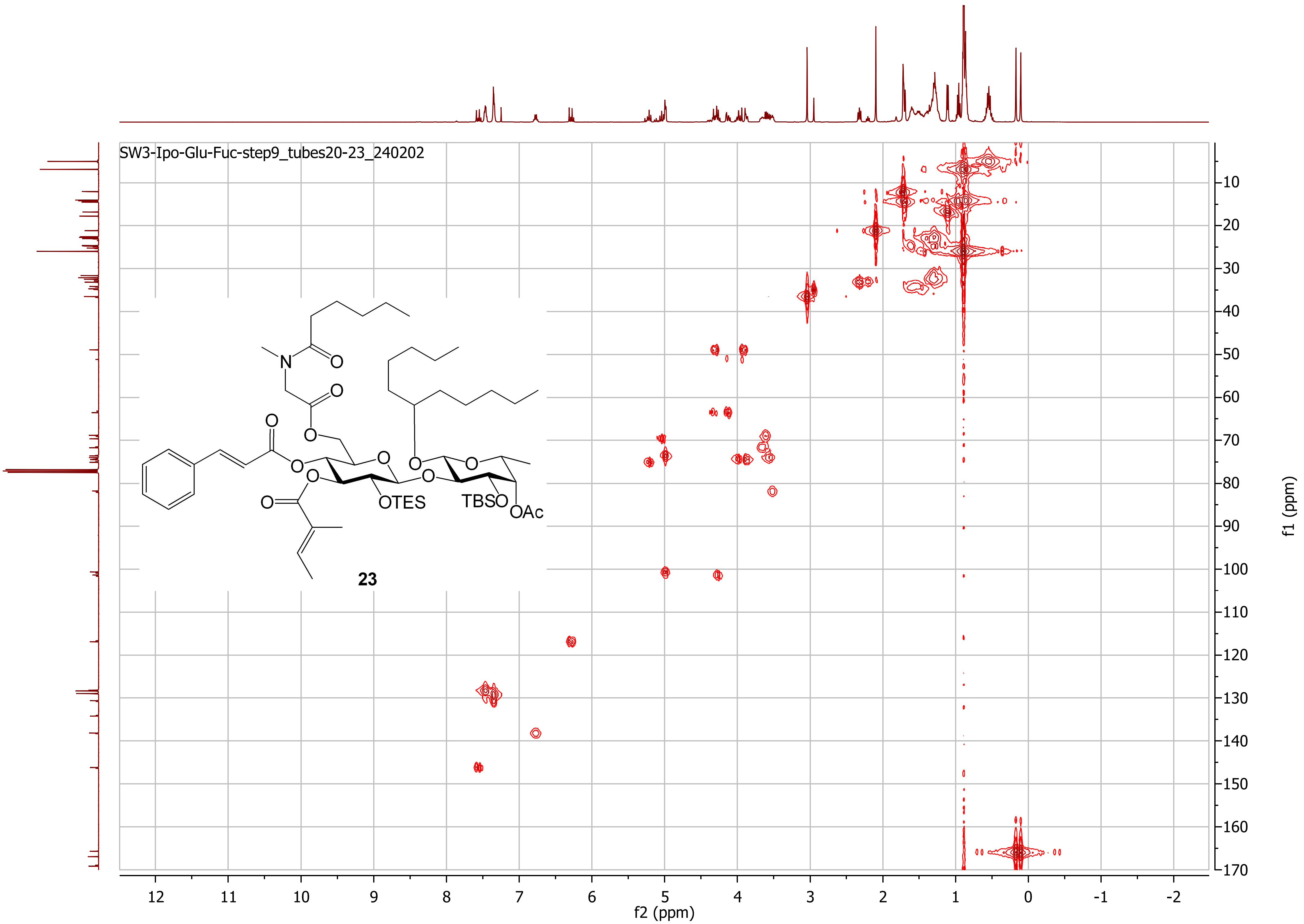
SW3-Ipo-Glu-Fuc-step9_tubes20-23_240202
O
N
O
O
O
O
O
O
O
O
OTES
TBSO
OAc
O
23
f2 (ppm)
f1 (ppm)

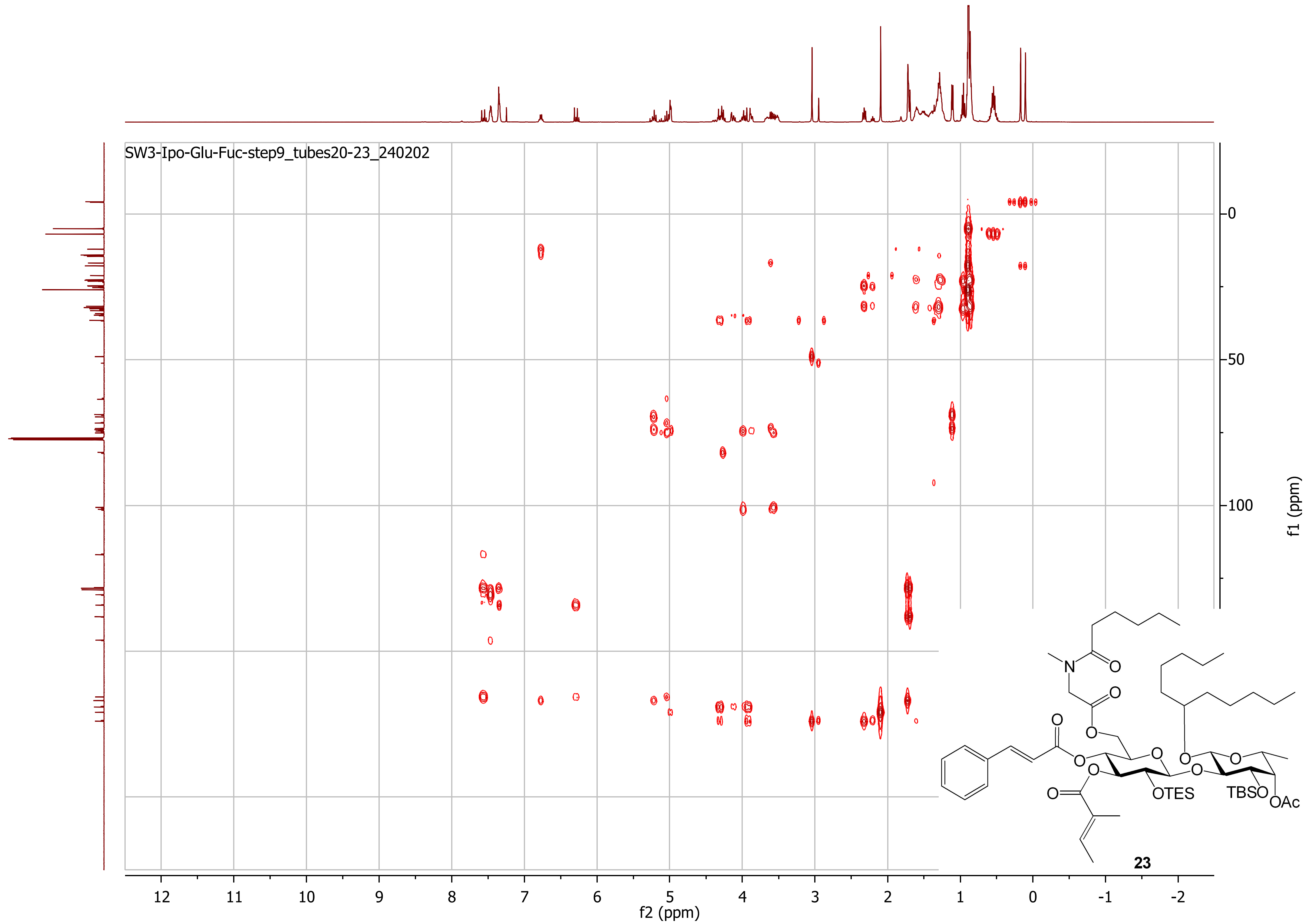
SW3-Ipo-Glu-Fuc-step9_tubes20-23_240202
f2 (ppm)
f1 (ppm)
23

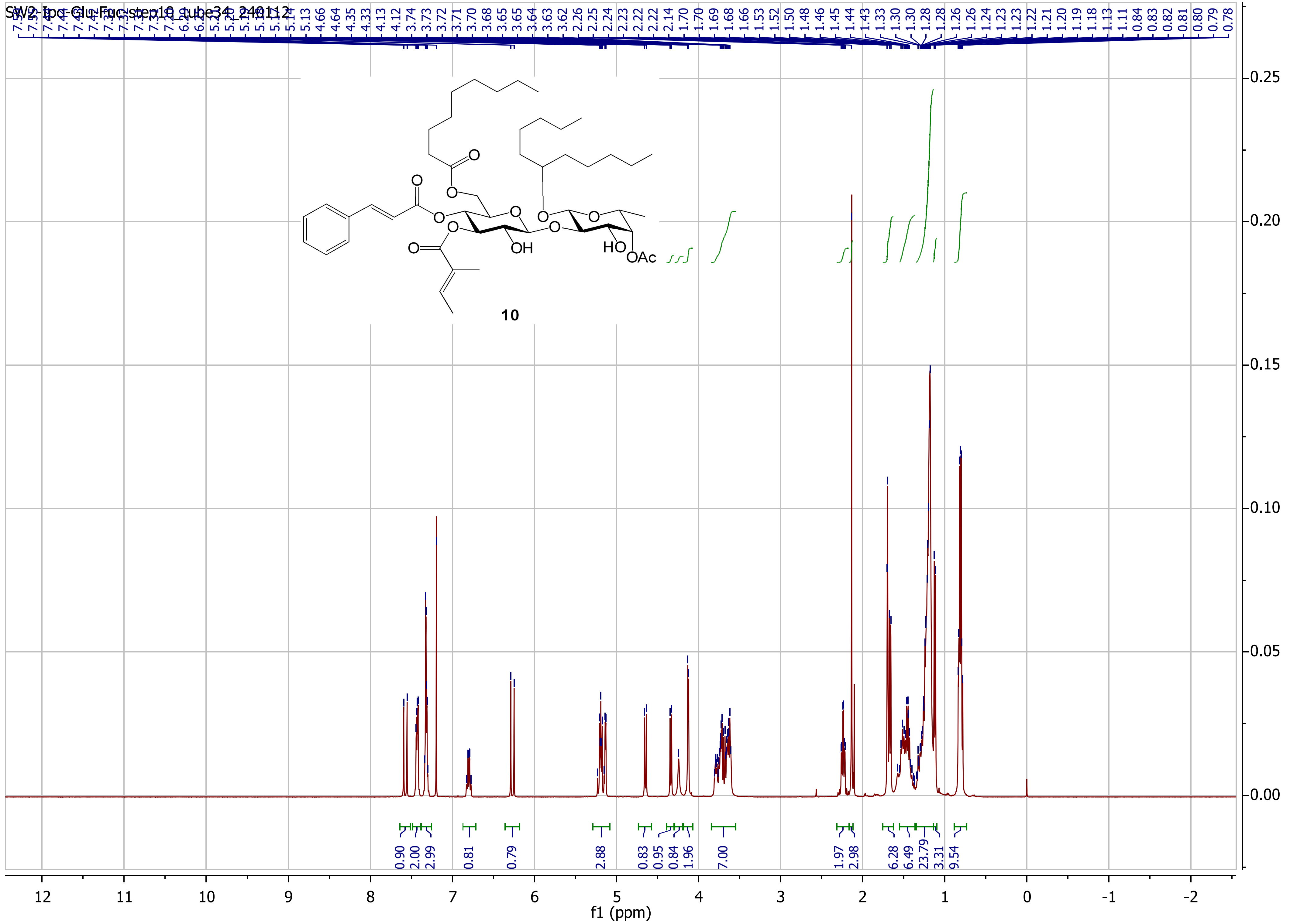
SW2-Ipq-Glu-Fuc-step10_tube34_240112
10
OH
HO
OAc
f1 (ppm)
0.90
2.00
2.99
0.81
0.79
2.88
0.83
0.95
0.84
1.96
7.00
1.97
2.98
6.28
6.49
23.79
3.31
9.54

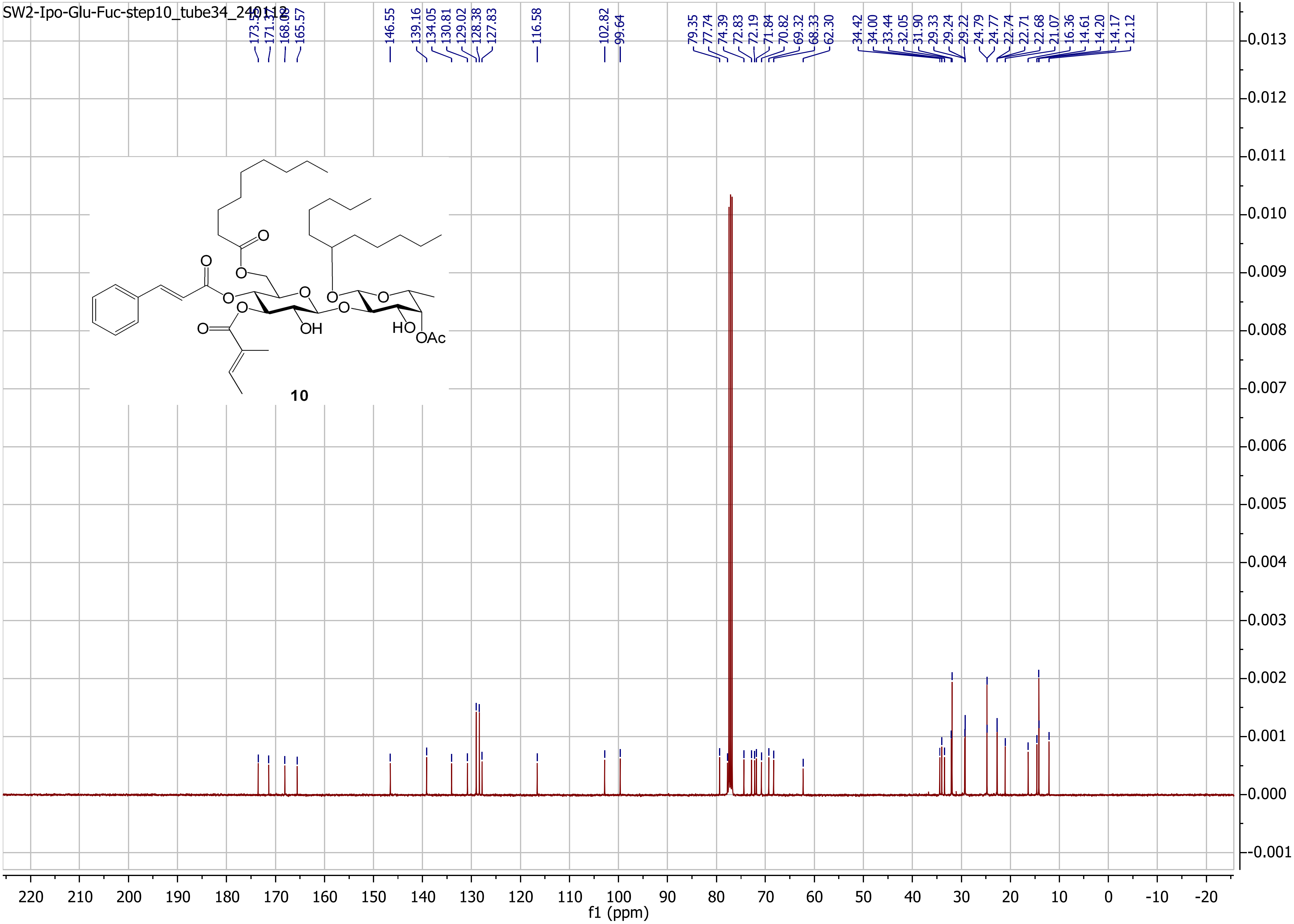

SW2-Ipo-Glu-Fuc-step10_tube34_240112
165.57
146.55
139.16
134.05
130.81
129.02
128.38
127.83
116.58
102.82
99.64
79.35
77.74
74.39
72.83
72.19
71.84
70.82
69.32
68.33
62.30
34.42
34.00
33.44
32.05
31.90
29.33
29.24
29.22
24.79
24.77
22.74
22.71
22.68
21.07
16.36
14.61
14.20
14.17
12.12
O
O
O
O
O
O
O
O
O
OH
HO
OAc
10
f1 (ppm)

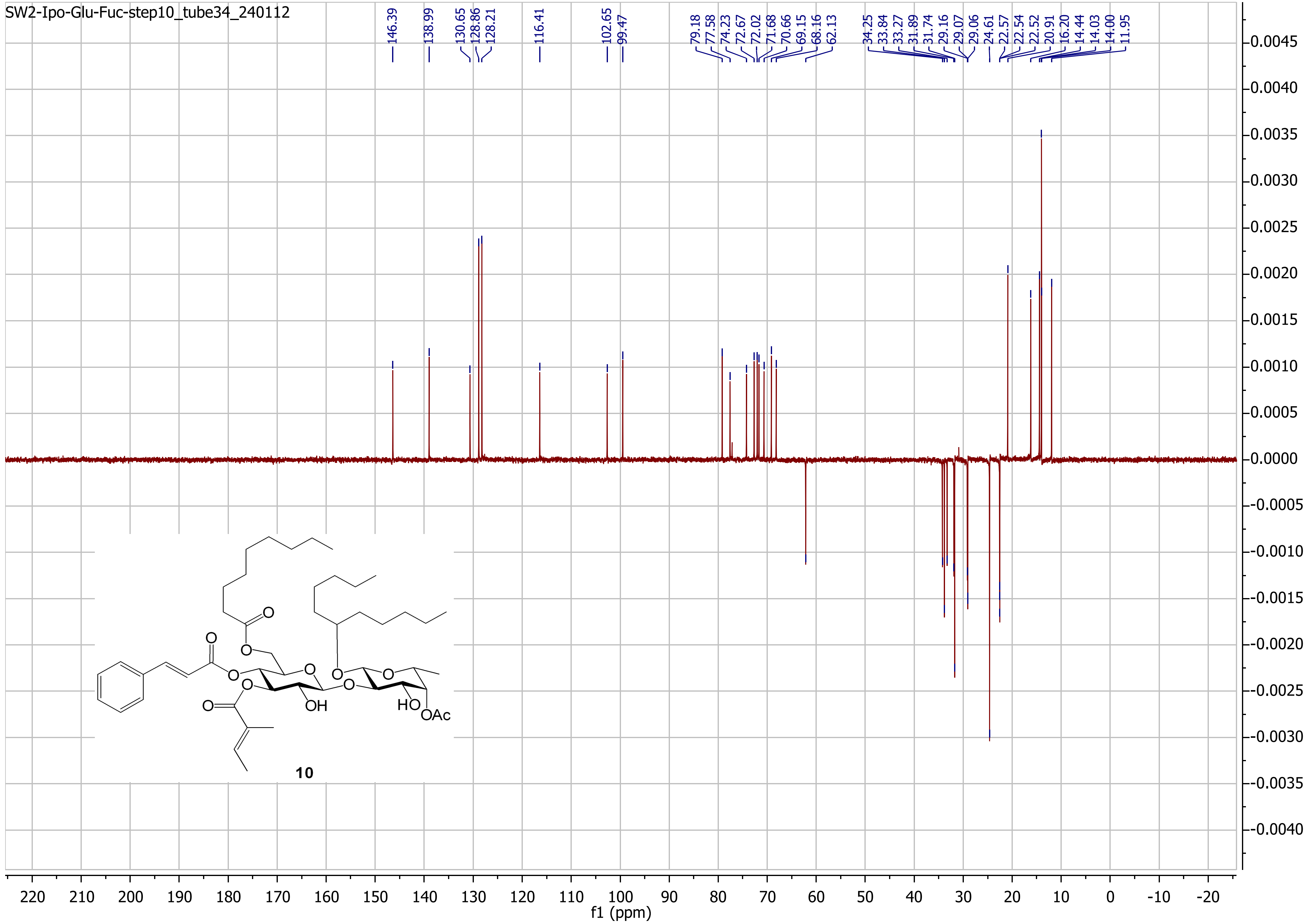
SW2-Ipo-Glu-Fuc-step10_tube34_240112
146.39
138.99
130.65
128.86
128.21
116.41
102.65
99.47
79.18
77.58
74.23
72.67
72.02
71.68
70.66
69.15
68.16
62.13
34.25
33.84
33.27
31.89
31.74
29.16
29.07
29.06
24.61
22.57
22.54
22.52
20.91
16.20
14.44
14.03
14.00
11.95
OH
HO
OAc
10
f1 (ppm)

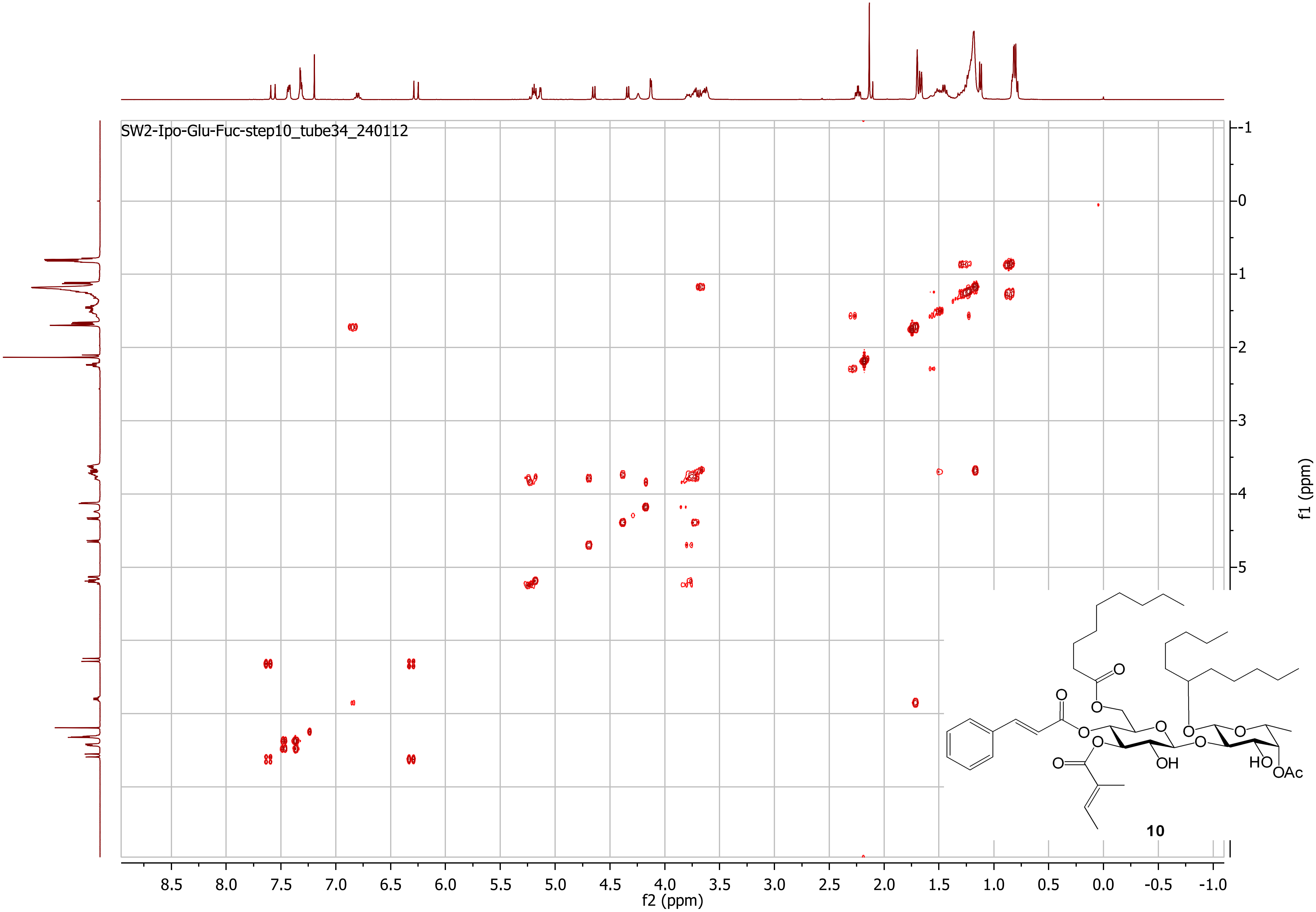

SW2-Ipo-Glu-Fuc-step10_tube34_240112
f2 (ppm)
f1 (ppm)
O
O
O
O
O
O
O
O
OH
O
O
HO
OAc
O
10

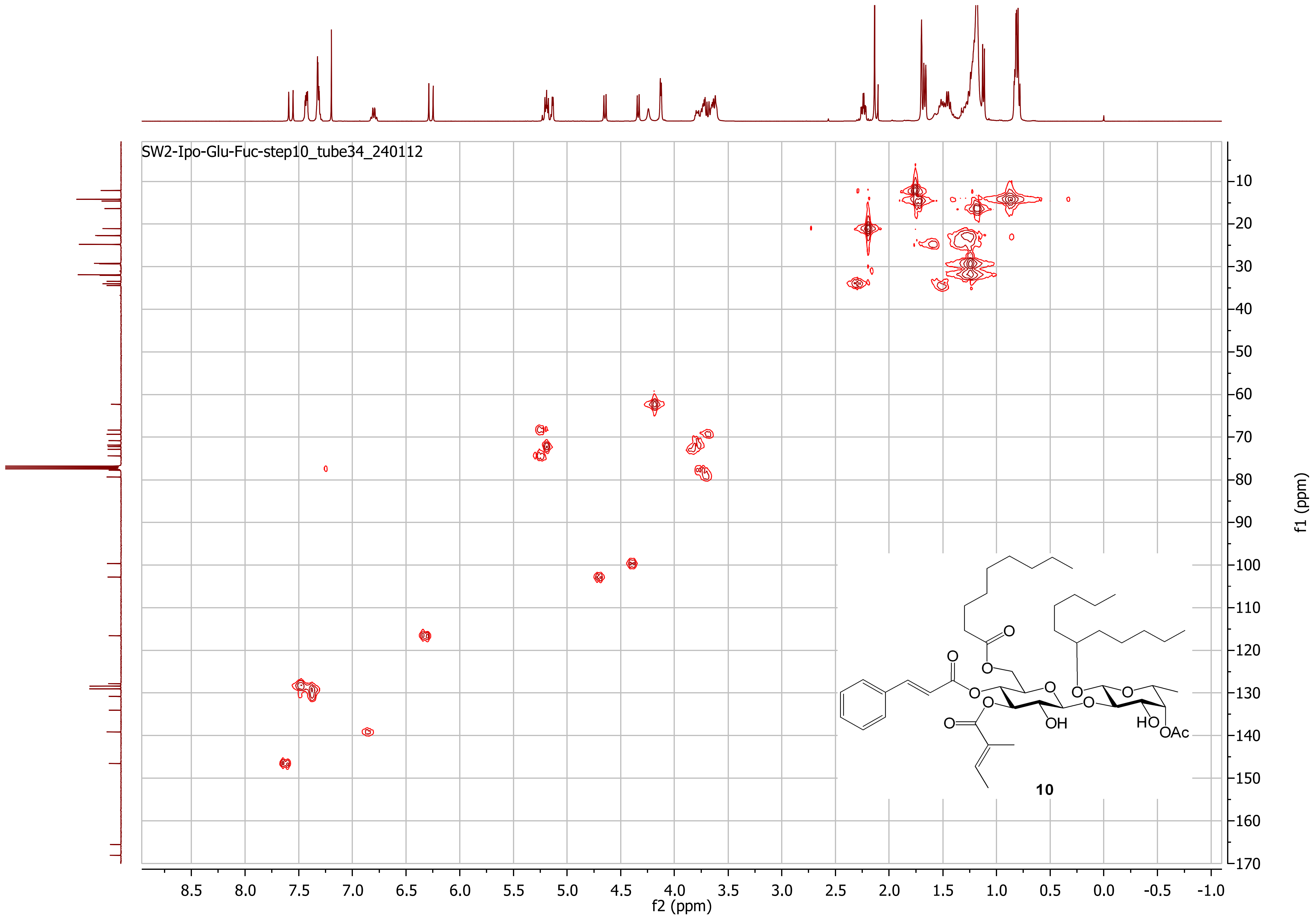

SW2-Ipo-Glu-Fuc-step10_tube34_240112
f2 (ppm)
f1 (ppm)
OH
HO
OAc
10

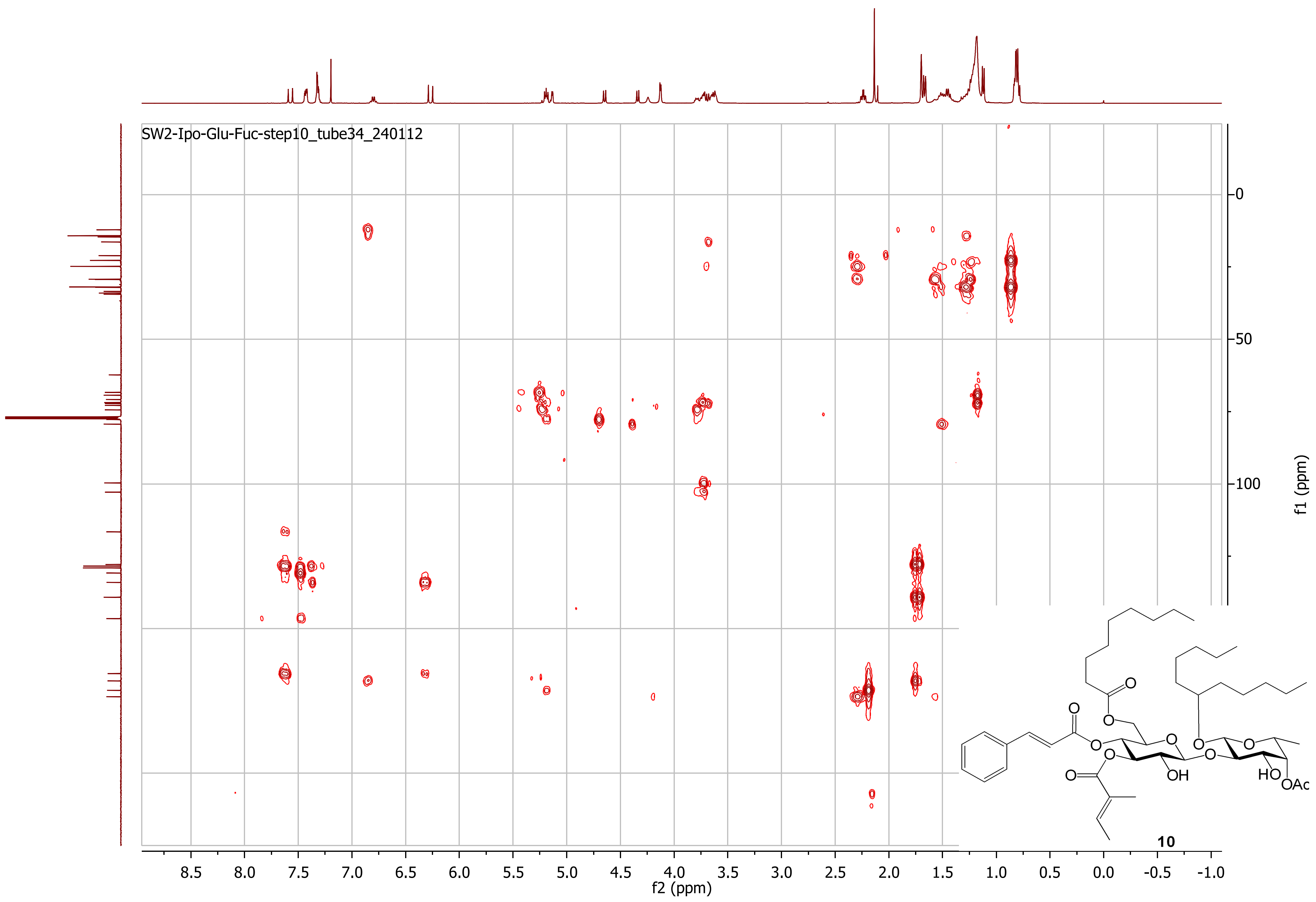
SW2-Ipo-Glu-Fuc-step10_tube34_240112
f2 (ppm)
f1 (ppm)
8.5
8.0
7.5
7.0
6.5
6.0
5.5
5.0
4.5
4.0
3.5
3.0
2.5
2.0
1.5
1.0
0.5
0.0
-0.5
-1.0
0
50
100
O
OH
HO
OAc
10

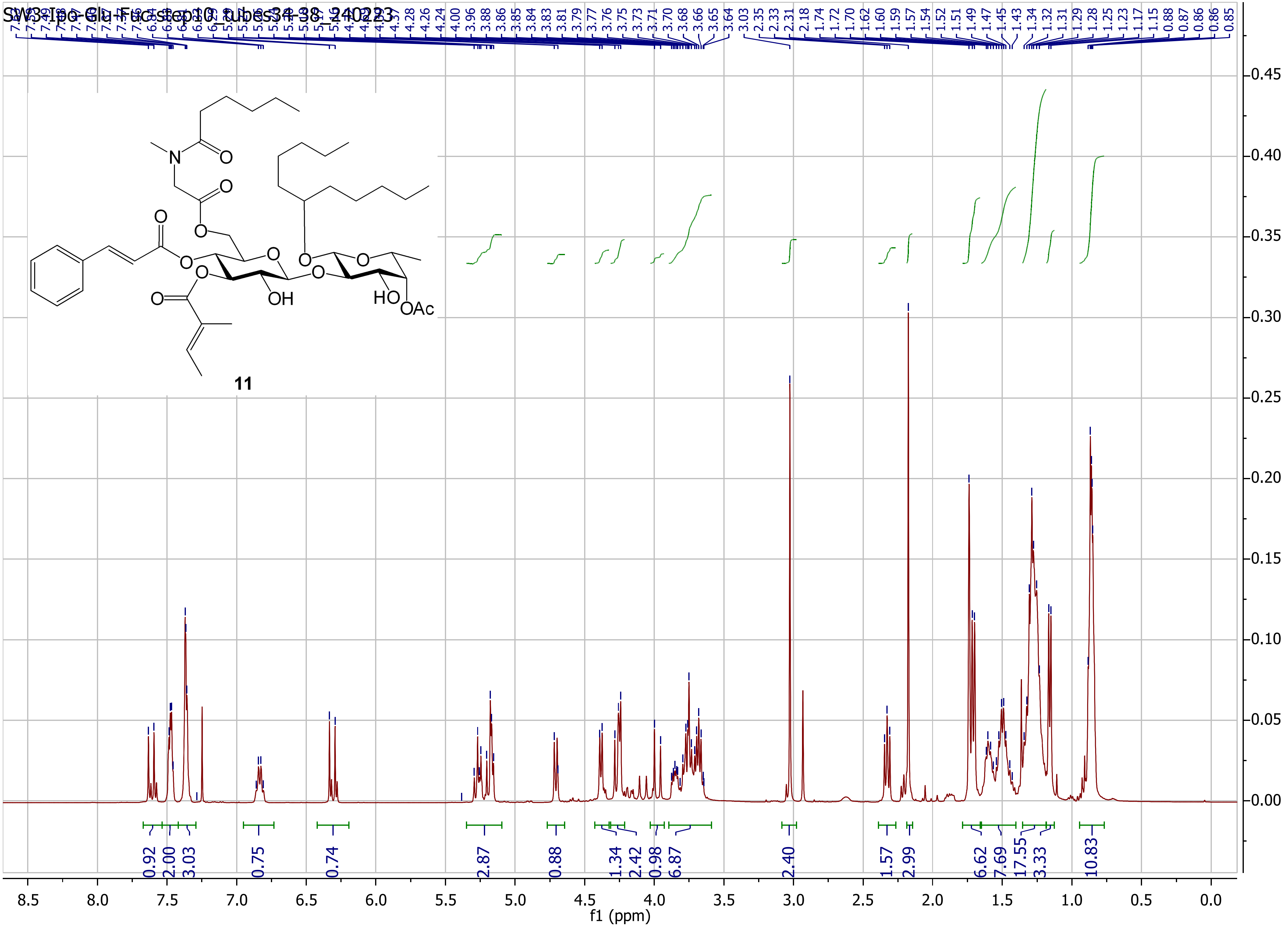
SW3-Ipo-Glu-Fuc step10_tubes34-38_240223
N
O
OH
HO
OAc
11
0.92
2.00
3.03
0.75
0.74
2.87
0.88
1.34
2.42
0.98
6.87
2.40
1.57
2.99
6.62
7.69
17.55
3.33
10.83
f1 (ppm)

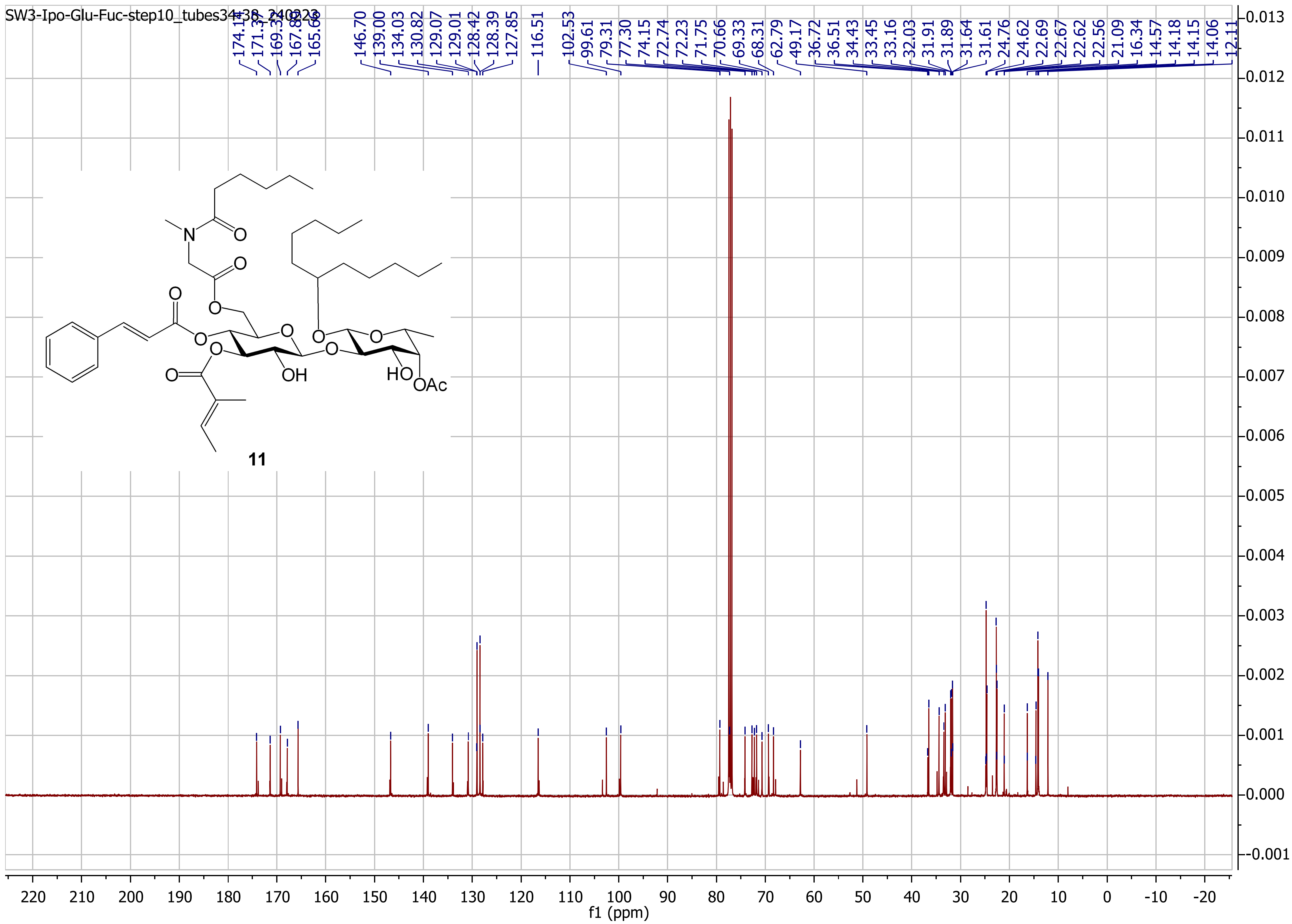
SW3-Ipo-Glu-Fuc-step10_tubes34-38_240223
174.1
171.3
169.3
167.8
165.6
146.70
139.00
134.03
130.82
129.07
129.01
128.42
128.39
127.85
116.51
102.53
99.61
79.31
77.30
74.15
72.74
72.23
71.75
70.66
69.33
68.31
62.79
49.17
36.72
36.51
34.43
33.45
33.16
32.03
31.91
31.89
31.64
31.61
24.76
24.62
22.69
22.67
22.62
22.56
21.09
16.34
14.57
14.18
14.15
14.06
12.11
N
O
OH
HO
OAc
11
f1 (ppm)

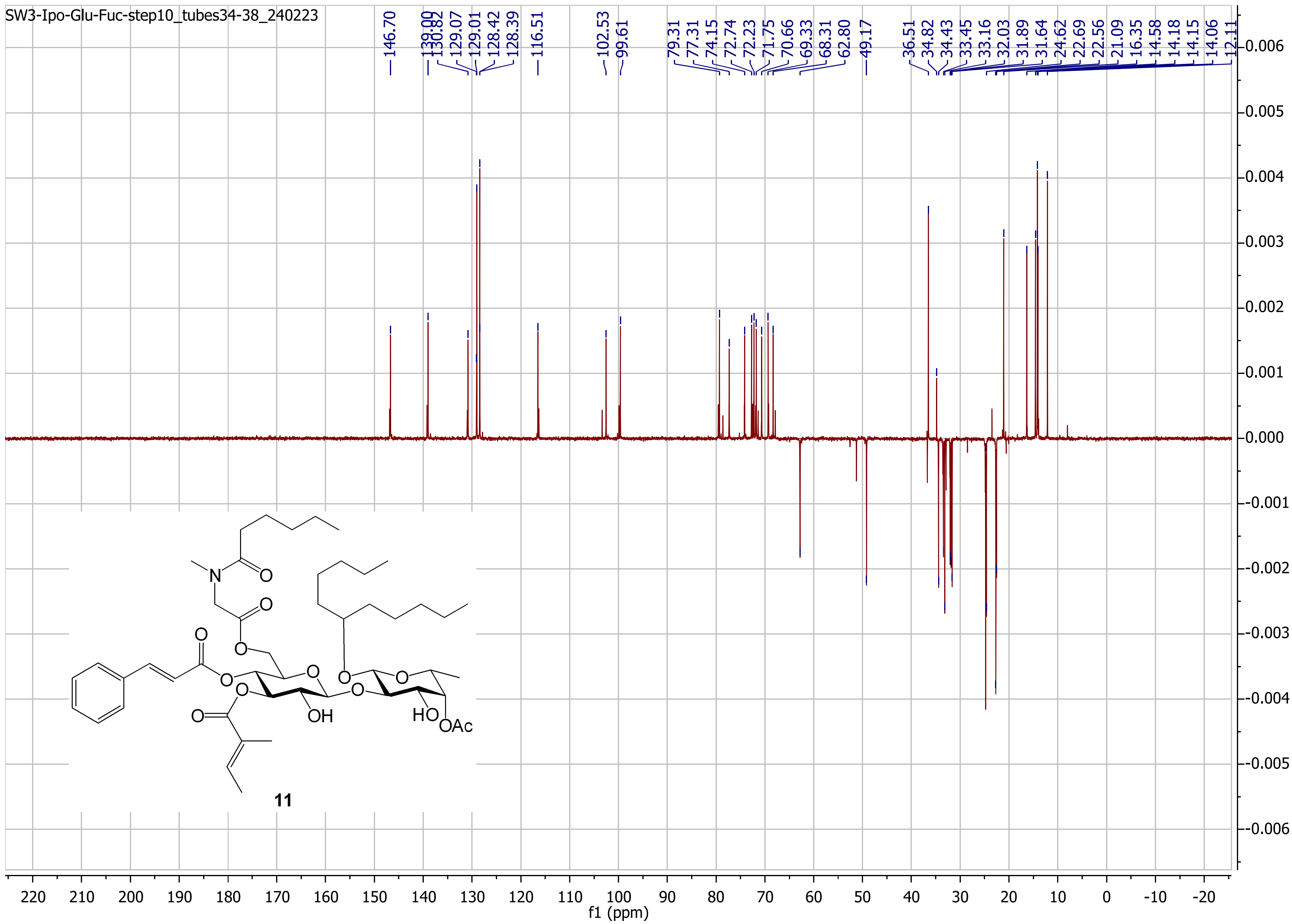
SW3-Ipo-Glu-Fuc-step10_tubes34-38_240223
146.70
139.00
130.82
129.07
129.01
128.42
128.39
116.51
102.53
99.61
79.31
77.31
74.15
72.74
72.23
71.75
70.66
69.33
68.31
62.80
49.17
36.51
34.82
34.43
33.45
33.16
32.03
31.89
31.64
24.62
22.69
22.56
21.09
16.35
14.58
14.18
14.15
14.06
12.11
0.006
0.005
0.004
0.003
0.002
0.001
0.000
-0.001
-0.002
-0.003
-0.004
-0.005
-0.006
220 210 200 190 180 170 160 150 140 130 120 110 100 90 80 70 60 50 40 30 20 10 0 -10 -20
f1 (ppm)
N
O
OH
HO
OAc
11

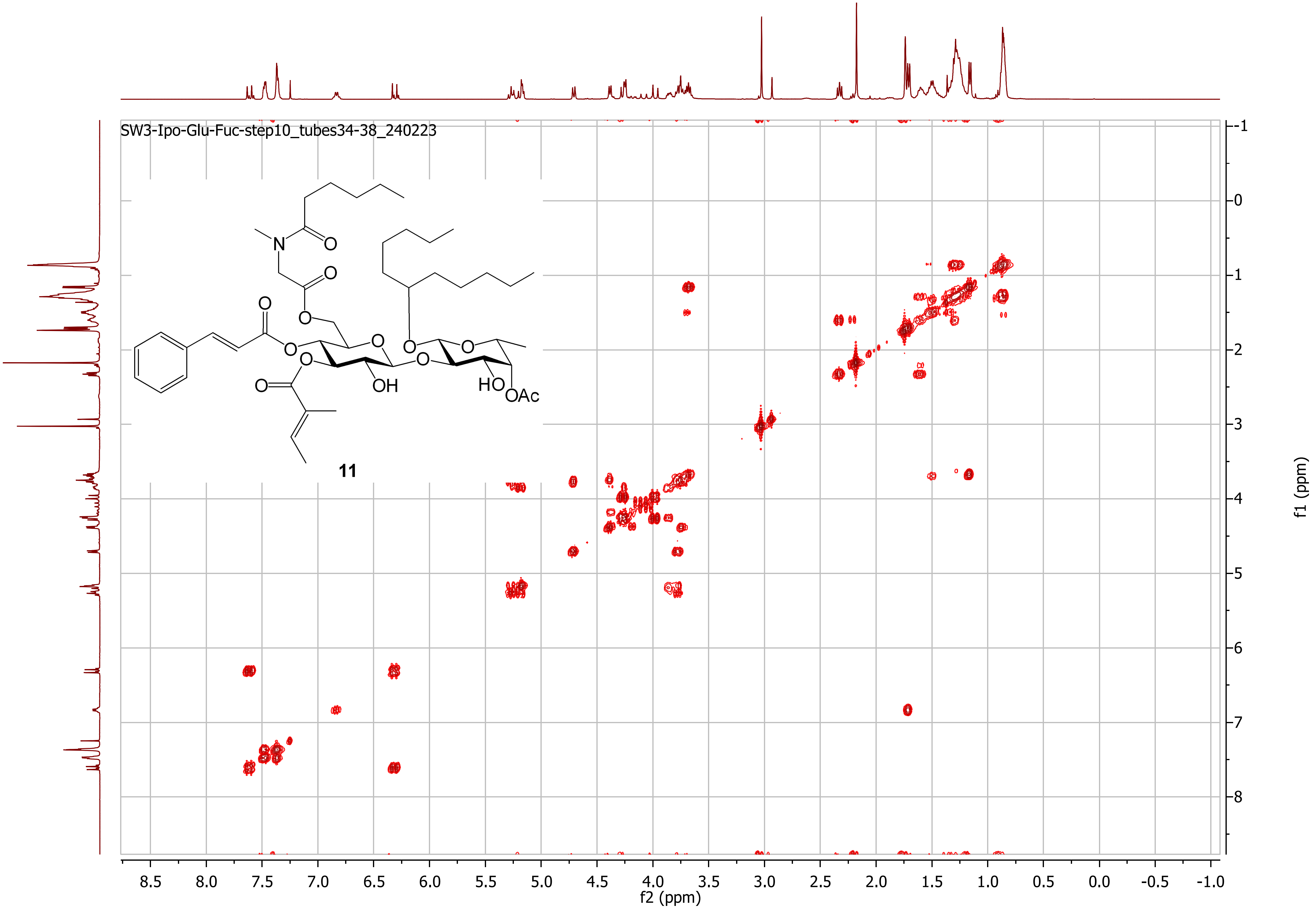
SW3-Ipo-Glu-Fuc-step10_tubes34-38_240223
11
f2 (ppm)
f1 (ppm)
8.5 8.0 7.5 7.0 6.5 6.0 5.5 5.0 4.5 4.0 3.5 3.0 2.5 2.0 1.5 1.0 0.5 0.0 -0.5 -1.0
-1 0 1 2 3 4 5 6 7 8

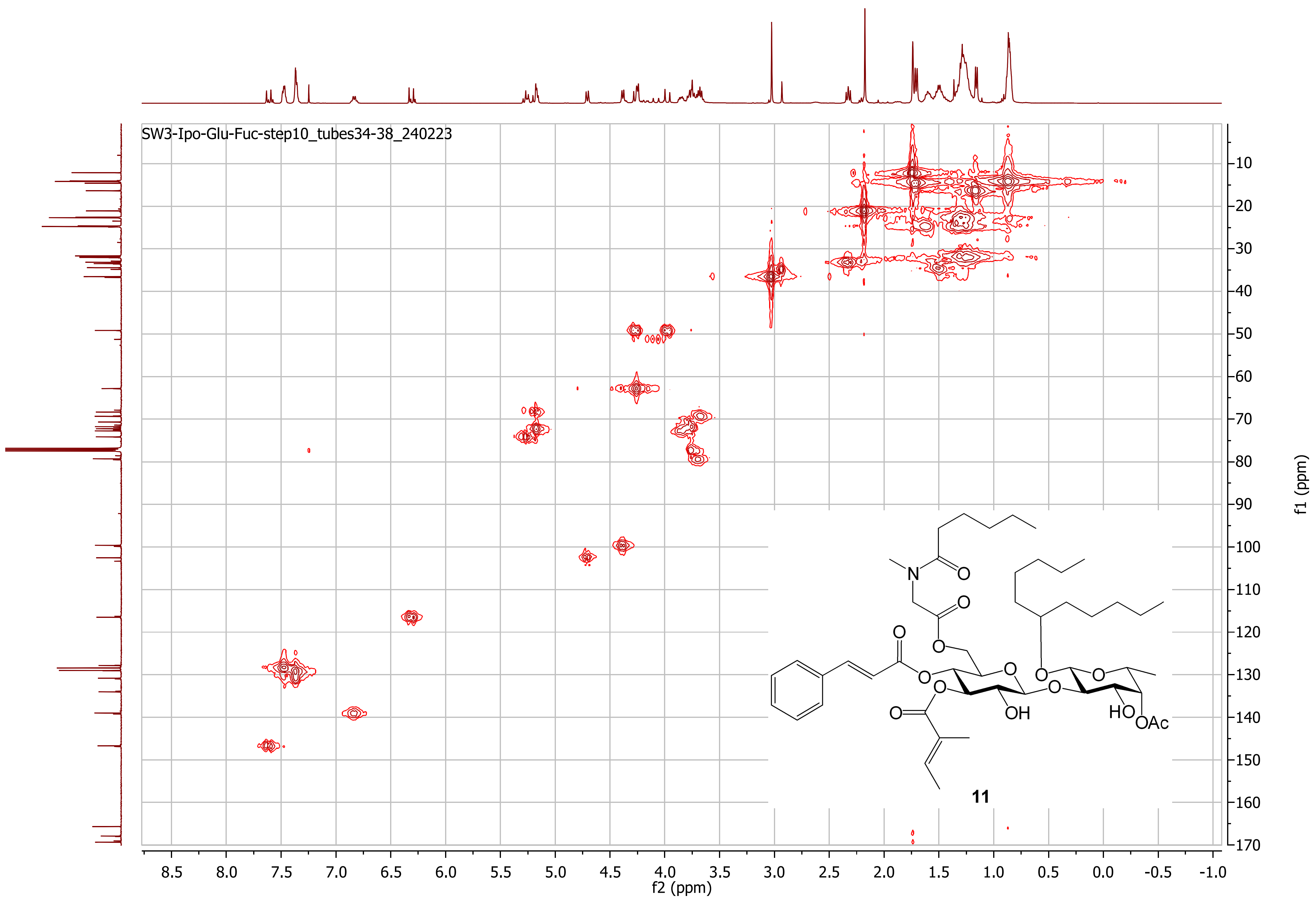
SW3-Ipo-Glu-Fuc-step10_tubes34-38_240223
f2 (ppm)
f1 (ppm)
8.5 8.0 7.5 7.0 6.5 6.0 5.5 5.0 4.5 4.0 3.5 3.0 2.5 2.0 1.5 1.0 0.5 0.0 -0.5 -1.0
10 20 30 40 50 60 70 80 90 100 110 120 130 140 150 160 170
N
O
OH
HO
OAc
11

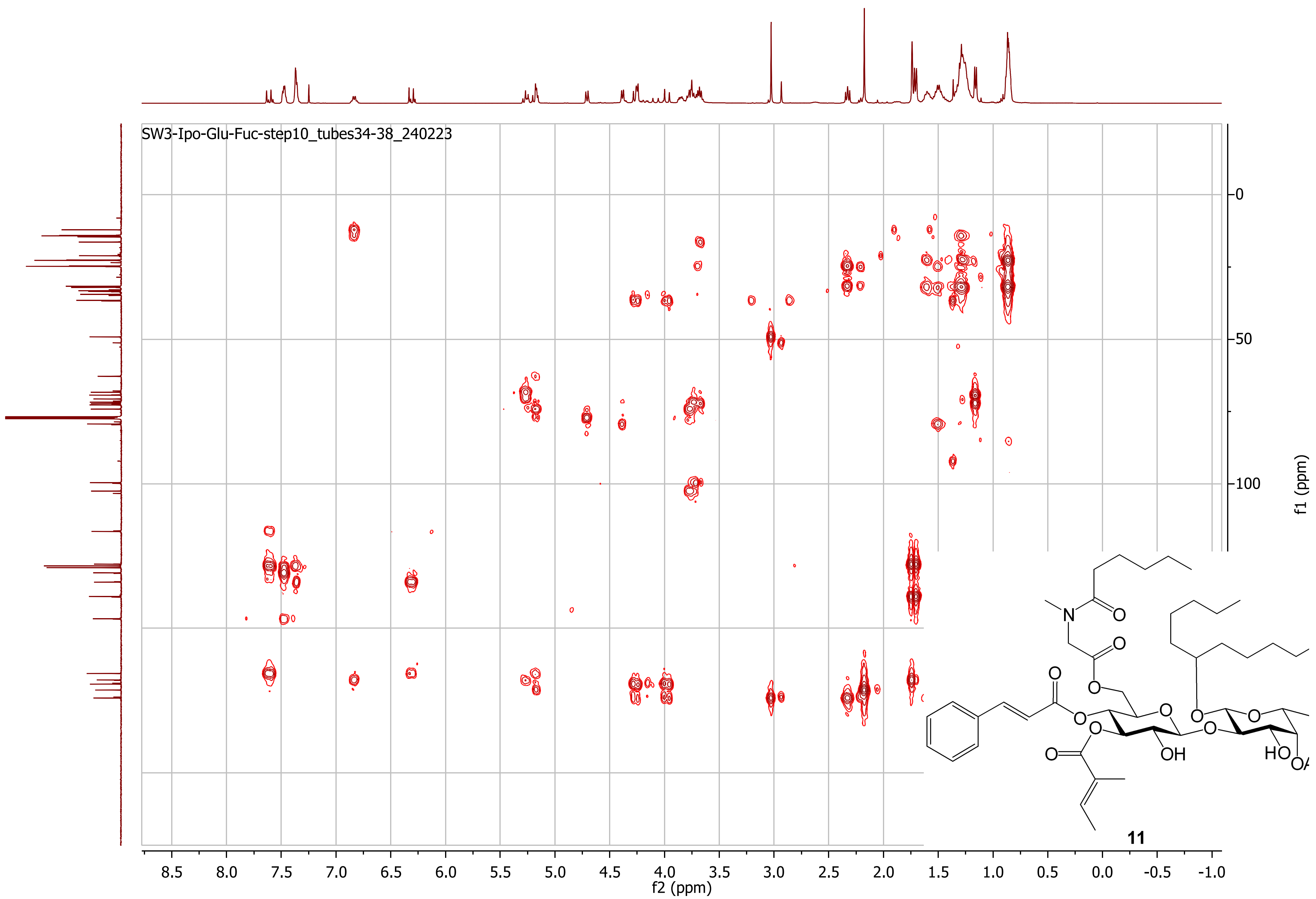
SW3-Ipo-Glu-Fuc-step10_tubes34-38_240223
f2 (ppm)
f1 (ppm)
N
O
O
O
O
O
O
O
O
O
O
OH
HO
11
